# Synthesis, Phase Engineering and Optoelectronic Applications of Binary Arsenic Sulphide Thin Films from Orpiment to Dimorphite

**DOI:** 10.3390/ma19194228

**Published:** 2026-10-05

**Authors:** Claudia Mihai, Dumitru Tsiulyanu, Alin Velea

**Affiliations:** 1 National Institute of Materials Physics, Atomistilor 405A, 077125 Magurele, Romania; claudia.mihai@infim.ro; 2 Department of Physics, Technical University of Moldova, bul. Dacia 41, MD-2060 Chisinau, Moldova; tsiudima@gmail.com

**Keywords:** arsenic sulphide, dimorphite, orpiment, realgar, chalcogenide thin films, phase engineering, photodarkening, magnetron sputtering, optoelectronic devices

## Abstract

**Highlights:**

- First review of the binary As-S system as a whole, covering orpiment, realgar and dimorphite thin films together.
- Deposition is phase-specific, As_4_S_3_ sublimes congruently, As_2_S_3_ evaporates incongruently and sputtering loses sulphur.
- Raman separates network, cage and polymorph below the phase fraction that grazing-incidence diffraction can detect.

**Abstract:**

Binary arsenic sulphide contains three thermodynamically stable compounds, orpiment (As_2_S_3_), realgar (As_4_S_4_) and dimorphite (As_4_S_3_), spanning a covalent layer network at the sulphur-rich end and discrete molecular cages at the arsenic-rich end. Thin-film research has concentrated almost entirely on As_2_S_3_, and no review has yet covered the binary system as a whole. This review brings together the phase diagram, the crystal structures of every stable phase and its polymorphs, the deposition methods, the structural and optical characterisation, the electrical and optoelectronic behaviour, and the device record. Deposition is compared across thermal evaporation, radio-frequency magnetron sputtering, pulsed laser deposition, plasma-enhanced chemical vapour deposition and solution routes, with attention to the two stoichiometry problems that govern film composition, incongruent evaporation of As_2_S_3_ and preferential sulphur sputtering from As-S targets. Phase engineering is treated as a distinct theme, since the arsenic-rich compositions crystallise as molecular cages whose polymorph ratio is set by growth rate, substrate temperature and annealing schedule. The review ends with the measurements that would bring the arsenic-rich compounds to the level of characterisation already available for As_2_S_3_, refractive index dispersion and non-linear coefficients, thin-film Raman assignments for each polymorph, and annealing schedules that identify which crystalline phase forms.

## 1. Introduction

Chalcogenide glasses and thin films combine wide infrared transparency, high refractive index, strong photosensitivity, and glass-forming ability across a wide range of compositions [1,2]. These properties have supported research on integrated photonics, holographic recording, infrared sensing, and, more recently, phase-change memory and neuromorphic computing [3,4]. Among chalcogenide systems, the binary arsenic–sulphur (As-S) system has three discrete stoichiometric compounds that are thermodynamically stable under ambient pressure, orpiment (As_2_S_3_), realgar (As_4_S_4_), and dimorphite (As_4_S_3_), ranging from a covalent layer network at the sulphur-rich end to discrete molecular cages at the arsenic-rich end, with different optical and electronic properties [5]. Their composition-tunable bandgap spans approximately 1.87 to 2.42 eV across the accessible stoichiometry range [6,7].

As_2_S_3_ has been the dominant subject of As-S thin-film research, owing to its transparency window extending to ~11.8 µm, its high non-linear refractive index (~100× silica), and its strong photosensitivity [3,8]. Thermally evaporated and sputtered As_2_S_3_ films are established platforms for mid-infrared waveguides, Bragg gratings, surface plasmon resonance sensors, and holographic recording media [9,10]. In contrast, As_4_S_4_ (and its polymorphs) has been studied primarily in single-crystal form for its photoinduced realgar-to-pararealgar structural transformation [11,12], with comparatively little work on thin-film deposition or device integration. As_4_S_3_, the arsenic-richest stable phase, known as a natural fumarolic mineral [13], has received almost no attention as a thin-film semiconductor. The first report of As_4_S_3_ thin films by thermal vacuum evaporation dates to 1996, when Tsiulyanu et al. [14] demonstrated photoconductivity with spectral maxima at 2.9 and 4.28 eV and established that the α/β phase ratio in the deposited films is controlled by evaporation rate. Subsequent work extended these observations to gas-sensing behaviour [15]. The imbalance in the thin-film literature therefore follows the structural change across the binary described in the preceding paragraph.

Despite the scientific and technological relevance of the full As-S compositional space, no comprehensive review of binary As-S thin films has been published. Existing reviews address either As_2_S_3_ alone in the context of specific applications [9,10,16] or treat the As-S system as a minor component within broad chalcogenide reviews [2,3]. The polymorphism of As_4_S_4_, the synthesis and properties of As_4_S_3_ thin films, the competition between crystalline phases during annealing, and the composition-dependence of optical and electrical properties across the full As-S binary have not been systematically reviewed. This review addresses these gaps by providing a unified description of the binary As-S system, from phase diagram and crystal structures, through deposition and characterisation methods, to optical, electrical, and optoelectronic properties and device applications.

The review is organised as follows. Section 2 describes the As-S binary phase diagram and the crystal structures and fundamental properties of each stable phase. Section 3 covers synthesis and thin-film deposition methods, comparing thermal evaporation, radio-frequency (RF) magnetron sputtering, pulsed laser deposition, plasma-enhanced chemical vapour deposition (PECVD), and solution processing in terms of achievable film composition, structure, and quality. Section 4 reviews structural characterisation of As-S thin films with emphasis on phase identification and the amorphous-to-crystalline transformation under annealing. Section 5 addresses optical properties and photoinduced phenomena across the full composition space. Section 6 covers electrical and optoelectronic properties. Section 7 reviews device applications from integrated photonics to sensors and memory devices. Section 8 provides conclusions and identifies open challenges.

## 2. The As-S Binary System, Phase Diagram, Crystal Structures and Properties

### 2.1. Phase Diagram and Stable Phases

The arsenic-sulphur binary system contains three thermodynamically stable intermediate compounds under ambient pressure: As_2_S_3_ (orpiment, 40 at. % As), As_4_S_4_ (realgar, 50 at. % As), and As_4_S_3_ (dimorphite, 57.1 at. % As). The most comprehensive thermodynamic assessment of the system, reported by Emelina et al. using CALculation of PHAse Diagrams (CALPHAD)-type modelling [5], established the full p–T–x phase diagram, confirming that all three intermediate phases melt congruently and identified two liquid–vapour azeotropes, one near the As_2_S_3_ composition and one near 4:3. The same system was later re-optimised by Prostakova et al., who critically reviewed the published thermochemical and phase-equilibrium data, found a significant discrepancy among the reported data sets, and derived a new set of model parameters for the liquid phase [17]. Several thermal properties are summarised in Table 1. As_2_S_3_ melts at 307 °C, As_4_S_4_ at 309 °C, and As_4_S_3_ at 211 °C. As_4_S_3_ additionally undergoes two solid-state transitions before melting, to paradimorphite at 149 °C and to a plastic crystal phase at 159 °C [18]. The As-S melt is strongly non-ideal, since arsenic disrupts the S_8_ ring structure of molten sulphur and promotes cross-linked As-S polymer chains, markedly increasing viscosity and strongly favouring glass formation over crystallisation [5]. This tendency is exploited in thin-film deposition, where as-deposited films of any composition in the As-S system are generally amorphous regardless of substrate temperature, with crystallisation requiring deliberate thermal annealing.

A notable feature confirmed by Emelina et al. is that As_4_S_3_ sublimes congruently [5], that is, the vapour phase has the same stoichiometry as the solid. Both orpiment and realgar also sublime congruently under equilibrium conditions [5]. This applies to equilibrium conditions only. In a practical deposition the source is molten and the evaporation rate is finite, and the behaviour of As_2_S_3_ under those conditions is described in Section 3.2. The practical significance for thermal evaporation is that films deposited from a stoichiometric As_4_S_3_ source retain the source composition without preferential loss of volatile species, an advantage that does not carry over to sputtering (Section 3).

A metastable pentasulphide As_2_S_5_ has been reported at elevated pressures and can be synthesised under specific conditions, but it is not thermodynamically stable at ambient pressure [5]. Similarly, As_4_S_5_ (uzonite, a natural mineral) has been detected as an intermediate phase during the photoinduced transformation of realgar [11] but is not a stable binary compound under ambient conditions.

**Figure 1:**
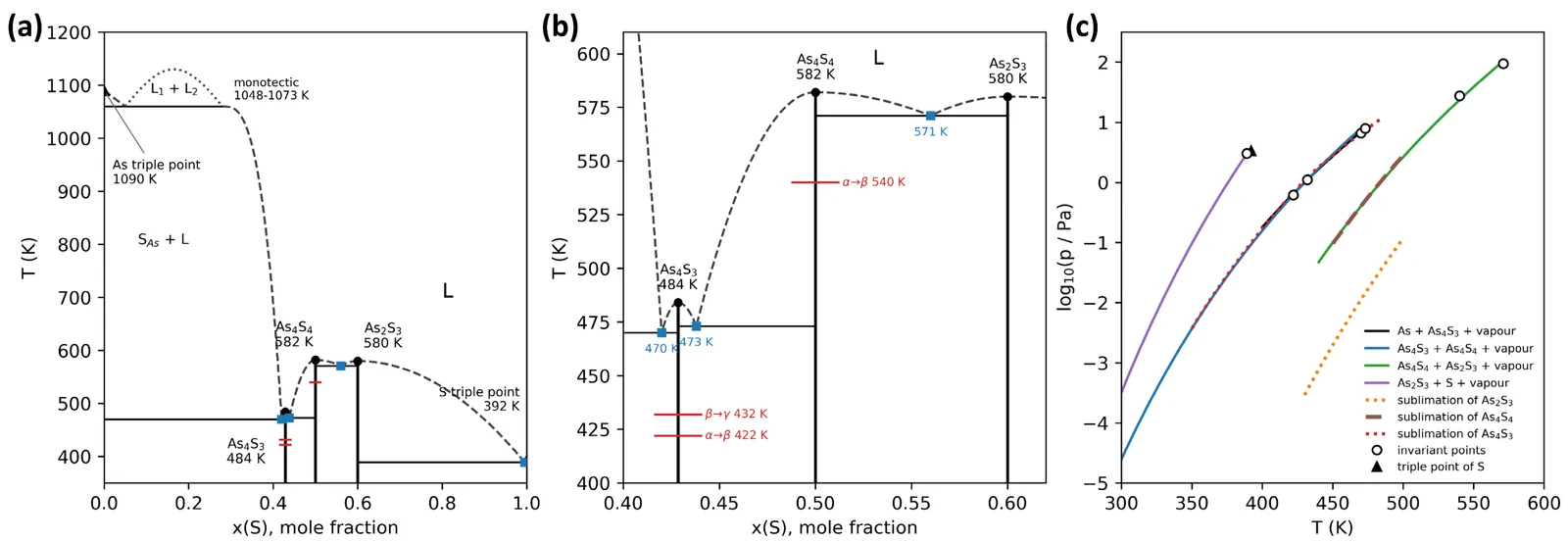
Phase diagram of the As-S binary system. (**a**) Temperature against composition over the full range. The congruent melting points of As_4_S_3_ (484 K), As_4_S_4_ (582 K) and As_2_S_3_ (580 K), the four eutectics (squares), the polymorphic transitions of As_4_S_3_ and As_4_S_4_ (red marks) and the triple points of arsenic and sulphur are placed at the values reported in [5], and the liquidus branches between them (dashed) are schematic. The liquid miscibility gap in the arsenic-rich region (L_1_ + L_2_, dotted) is drawn schematically at the monotectic temperature of 1048 to 1073 K reported in [17]. (**b**) The same projection over the composition range x(S) = 0.40 to 0.62. (**c**) Vapour pressure against temperature, plotted from the equations of [5] for the four equilibria between two solid phases and the vapour (solid lines) and for the sublimation of the three compounds (dotted and dashed lines), with the invariant points of [5] (open circles). The As_4_S_3_ sublimation curve is calculated from the sublimation enthalpies and entropies of its three polymorphs given in [5], with a standard pressure of 10^5^ Pa. The As_4_S_4_ sublimation curve coincides with the As_4_S_4_ + As_2_S_3_ + vapour curve within the line width. Redrawn from the data of [5,17].

Figure 1 shows the phase diagram redrawn from the data of [5,17]. The temperature-composition projection in Figure 1a spans the full binary system and locates the three congruently melting compounds and the four eutectics, and Figure 1b shows the same projection over the composition interval containing As_4_S_4_ and As_2_S_3_, whose melting points lie within 2 °C of one another. The pressure-temperature projection in Figure 1c gives the vapour pressure of the four three-phase equilibria and the sublimation curves of the three compounds.

### 2.2. Crystal Structures and Molecular Architecture

**Figure 2:**
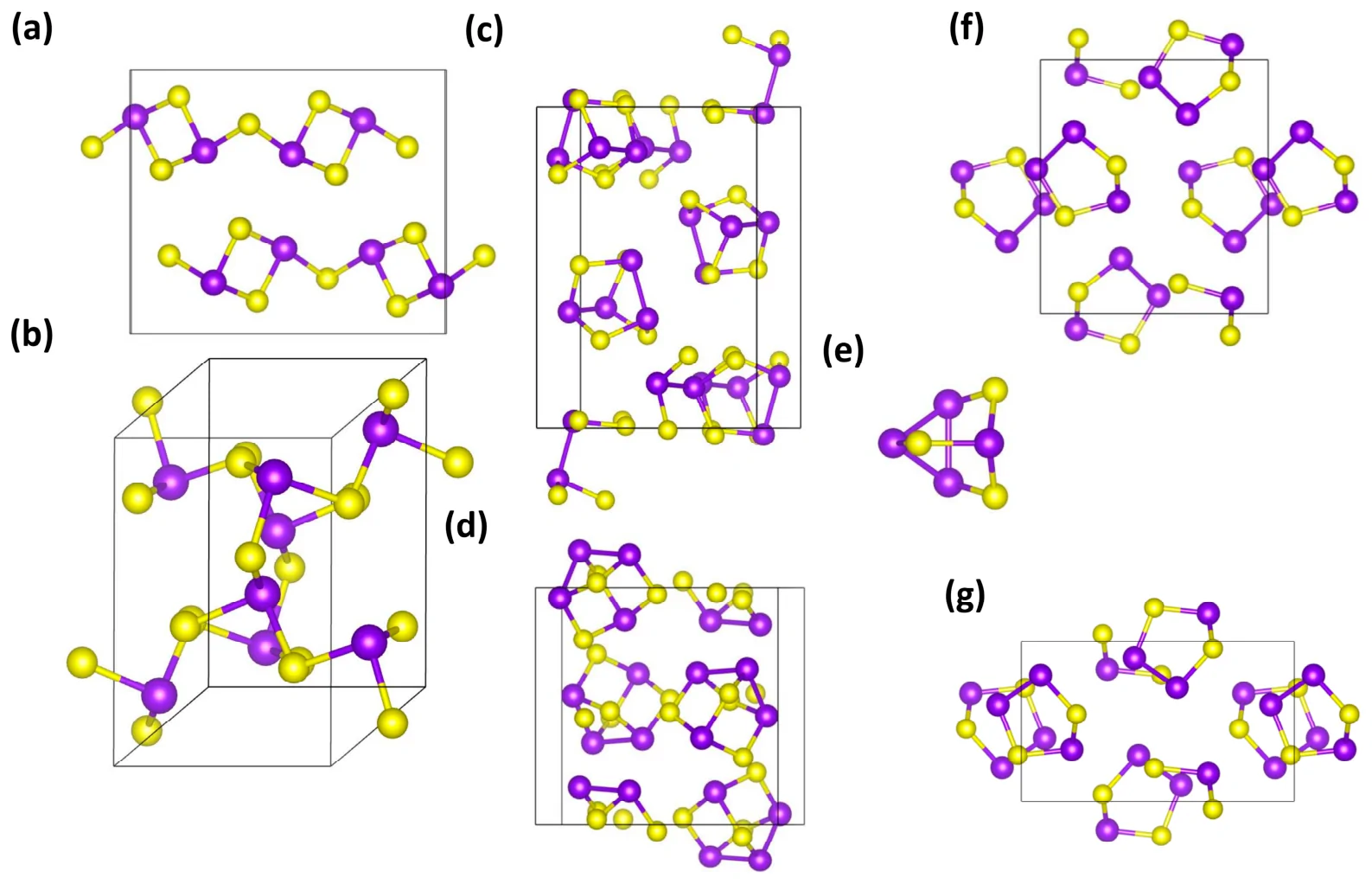
Crystal structures of the stable binary As-S phases and their polymorphs, rendered in VESTA [19] from published structural coordinates. (**a**) Orpiment [20] and (**b**) anorpiment [21], both As_2_S_3_, differing in the stacking of identical layers. (**c**) Realgar [20] and (**d**) pararealgar [12], both As_4_S_4_, differing by a linkage isomerisation of the cage. (**e**) The isolated As_4_S_3_ cage [22], with the triangular As_3_ base and the three As-As bonds of the cage, against two in realgar and none in orpiment. (**f**) Dimorphite [22] and (**g**) paradimorphite [13], both As_4_S_3_, drawn from a common viewpoint, with an identical cage and a different packing orientation.

All three stable binary As-S compounds, and their polymorphs, are built from covalently bonded units held together in the crystal by van der Waals forces, discrete molecular cages in realgar and dimorphite and infinite corrugated layers in orpiment. This distinguishes the As-S system from most inorganic semiconductors, in which the covalent bonding extends in all three dimensions, and has direct consequences for material properties, giving relatively low melting points, broad glass-forming tendency, low mechanical hardness, and photosensitivity driven by bond rearrangements within or between these units. All the structures discussed in this section are compared in Figure 2, which places the two As_2_S_3_ dimorphs, the two As_4_S_4_ isomers, the isolated As_4_S_3_ cage and the two As_4_S_3_ polymorphs on a common scale and viewpoint.

#### 2.2.1. As_2_S_3_, Orpiment

Orpiment crystallises in the monoclinic space group P2_1_/n (No. 14) with a = 11.475 Å, b = 9.577 Å, c = 4.256 Å, β = 90°41′, Z = 4 [20]. Unlike realgar and dimorphite, the orpiment structure is not built from isolated cages but from infinite corrugated (010) layers of AsS_3_ trigonal pyramidal units linked by bridging sulphur atoms. The layers are held together by van der Waals forces, producing perfect (010) cleavage. Each As atom is three-fold coordinated to three bridging S atoms and each S atom in turn bridges two As atoms, so the layer contains no singly-bonded sulphur and no dangling bonds [20], with As-S bond lengths of 2.243–2.308 Å (mean 2.283 Å) and S-As-S angles spanning 92.8–105.0° [20]. The sulphur atom bridging between adjacent spiral chains subtends an unusually compressed As-S-As angle of 87.9°, against 103.7° and 101.0° for the sulphurs bridging within a chain, which reflects the geometric strain of the linkage between chains [20]. The lone pair on each As atom points perpendicular to the layer. A triclinic polymorph, anorpiment (space group P$\bar{1}$), has been approved as a distinct mineral species by the International Mineralogical Association (IMA) under the number 2011-014 [21]. Anorpiment has unit cell parameters a = 5.7577 Å, b = 8.7169 Å, c = 10.2682 Å, α = 78.15°, β = 75.82°, γ = 89.86°, V = 488.37 Å^3^, Z = 4, and a calculated density of 3.346 g cm^−3^, lower than orpiment (3.494 g cm^−3^) [21]. The structure retains the same layered As_2_S_3_ macromolecular architecture (AsS_3_ pyramidal units forming six-membered rings, van der Waals interlayer bonding), but with a different stacking sequence: in orpiment, adjacent AsS_3_ pyramids have their As apices oriented in approximately the same direction, whereas in anorpiment each apex points approximately opposite to that of its neighbour. This difference precludes polytypism and makes anorpiment and orpiment true dimorphs and not polytypes [21]. Anorpiment was found as a low-temperature hydrothermal phase and is not expected to occupy a stability field on the equilibrium P-T phase diagram. Its formation is attributed to non-equilibrium (templating or biogenic) processes [21]. The elastic, vibrational and electronic properties calculated for this structure are given in Section 2.3 [23].

#### 2.2.2. As_4_S_4_, Realgar and Its Polymorphs

At least three distinct crystalline phases occur at the As:S = 1:1 stoichiometry. Realgar (α-As_4_S_4_) is the thermodynamically stable form in ambient conditions, crystallising in monoclinic P2_1_/n (a = 9.325 Å, b = 13.571 Å, c = 6.587 Å, β = 106°23′, Z = 4) [20]. Its structure consists of discrete As_4_S_4_ cage molecules with approximate C_2v_ symmetry (a cradle-like arrangement), where each As atom is three-fold coordinated to two S neighbours and one As neighbour (one As-As bond, two As-S bonds per As atom). The cage contains two As-As bonds (2.566–2.571 Å) and eight As-S bonds (2.228–2.247 Å, mean 2.237 Å), with each of the four S atoms bridging two As atoms in an alternating As-As-S-As-As-S arrangement [20]. The S-As-S angles span 94.5–95.1°, the As-As-S angles 98.7–100.0°, and the As-S-As angles 100.8–101.3°. Realgar is orange-red, consistent with the bandgap given in Section 2.3, and has been known since antiquity as a pigment and pyrotechnic material.

Above 540 K (267 °C) realgar converts to β-As_4_S_4_, the high-temperature polymorph, built from the same As_4_S_4_ cage in a different packing and crystallising in the monoclinic space group C2/c [18,24]. The back transition on cooling occurs near 395 K, a hysteresis of about 145 K, so the phase is readily retained at room temperature by quenching [18], and a natural analogue occurs as a mineral [25].

Pararealgar (p-As_4_S_4_) is the stable photoproduct of realgar under near-bandgap illumination. It crystallises in monoclinic P2_1_/c (a = 9.909 Å, b = 9.655 Å, c = 8.502 Å, β = 97.29°, Z = 4) [12]. The transition from realgar to pararealgar involves a photoinduced linkage isomerisation of the As_4_S_4_ cage: one As-As bond breaks and a new As-S bond forms, converting the cradle cage to a wing-like configuration. The linkage isomerisation has been confirmed directly by in situ diffraction, and the intermediate As_4_S_5_ species detected in the same experiment is described in Section 4.7 [11]. It is irreversible under ambient conditions and wavelength-selective, and the active spectral range is given in Section 4.7 [24,26]. Alacranite, a distinct IMA mineral species, has the confirmed chemical formula As_8_S_9_ [25]. Its crystal structure (space group P2/c, monoclinic; a = 9.942 Å, b = 9.601 Å, c = 9.178 Å, β = 101.94°, V = 857.1 Å^3^, Z = 2) consists of an ordered sequence of alternating As_4_S_4_ and As_4_S_5_ cage-like molecules, not a single molecular species, whose alternating arrangement reduces the lattice symmetry from C (as in β-As_4_S_4_) to P [25].

**Figure 3:**
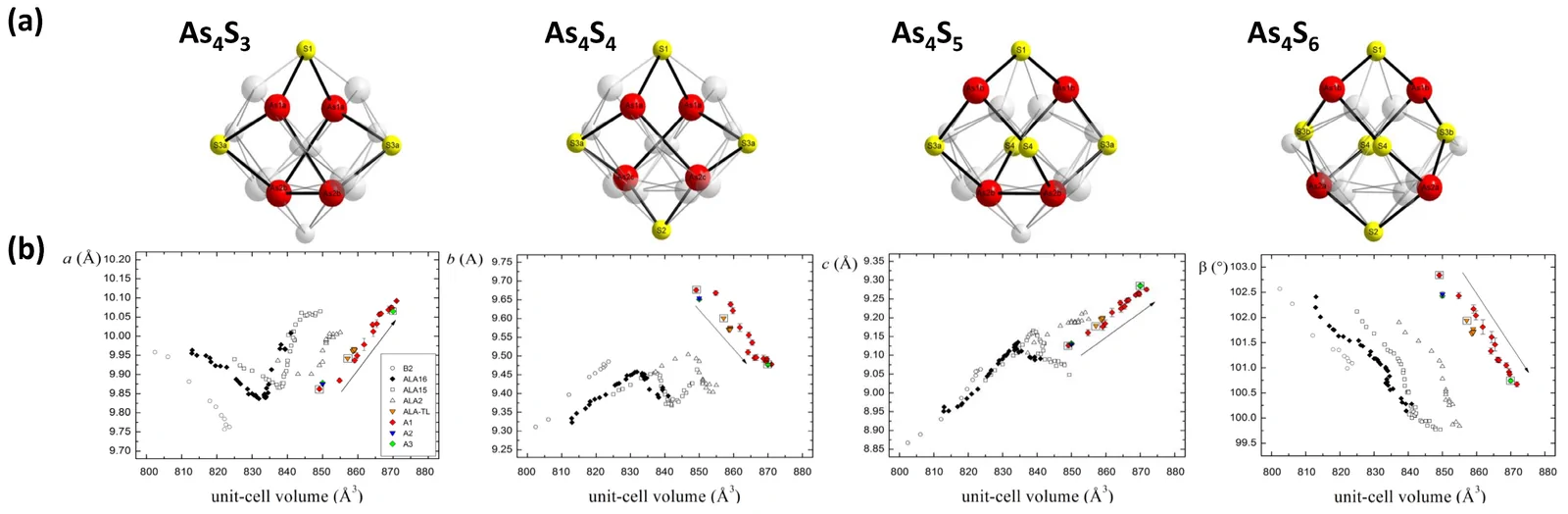
The As_4_S_n_ molecular series and its response to light. (**a**) The four molecular cages found in the disordered monoclinic C2/c structure, As_4_S_3_, realgar-type As_4_S_4_, uzonite-type As_4_S_5_ and As_4_S_6_. (**b**) Unit-cell parameters against unit-cell volume for alacranite crystals, for synthetic β-As_4_S_4_ and for non-stoichiometric As_8_S_9−x_ crystals, with arrows marking increasing exposure time. The crystals expand by 2.7% in volume under light passed through a 440 nm long-wavelength-pass filter, whereas a 550 nm filter produces no significant change. Reproduced from [27] under a Creative Commons Attribution 4.0 International Licence, © 2024 the authors.

The molecular units of the binary system are shown in Figure 3. Figure 3a shows the four cage molecules occurring in the disordered monoclinic C2/c structure, As_4_S_3_, the realgar-type As_4_S_4_, the uzonite-type As_4_S_5_ and As_4_S_6_, allowing the cage of every phase treated in this section to be compared on a common scale and distinguishing uzonite from the χ phase described in Section 4.3. Figure 3b reports the photoinduced response of alacranite, the unit-cell parameters being plotted against unit-cell volume for increasing exposure time. Under illumination transmitted by a 440 nm long-wavelength-pass filter the unit-cell volume increases by 2.7% after 1520 min, with a and c increasing and b and the β angle decreasing, and the crystal subsequently transforms to an orthorhombic phase of space group Pccn. Illumination transmitted by a 550 nm long-wavelength-pass filter produces no significant change in the unit cell, which localises the spectral range responsible for the transformation to between 440 and 550 nm [27].

#### 2.2.3. As_4_S_3_, Dimorphite and Paradimorphite

As_4_S_3_ has two polymorphs, dimorphite, the room-temperature stable form (the β-form [22]), and paradimorphite, the high-temperature polymorph (the α-form [28]), stable above 149 °C, IMA 2020-101 [13]. Reported temperatures for the dimorphite to paradimorphite conversion span 404 to 422 K (131 to 149 °C) [18]. Heat capacity measurements resolve three states below the melting point at 484 K (211 °C) [18]. Dimorphite converts to paradimorphite at 422 K (149 °C) with ΔH = 1.44 kJ/mol, and paradimorphite converts at 432 K (159 °C) with ΔH = 13.04 kJ/mol to a plastic phase in which the cages retain their positions but rotate freely, which is why this transition carries the larger enthalpy [18]. Paradimorphite is therefore the equilibrium phase over a window of only about 10 K, and it is recovered at room temperature by quenching [18]. The IMA explicitly abandoned the earlier α/β Greek-letter convention, introduced by Whitfield [22,28] with the inverse assignment to thermochemical convention (β = low-T, α = high-T), to eliminate persistent nomenclature confusion [13]. Both polymorphs are orthorhombic, space group Pnma (No. 62), but with substantially different lattice parameters reflecting different packing arrangements of the As_4_S_3_ cage (Table 2). The α and β descriptors are used here in the sense of Whitfield [22,28] and of the thin-film literature that follows it [14,15], in which α denotes the polymorph corresponding to IMA paradimorphite and β the polymorph corresponding to IMA dimorphite. Campostrini et al. [13] apply the two labels in the opposite sense, following the convention for temperature-dependent transitions. The IMA mineral names are given alongside the Greek labels throughout this review to remove the ambiguity.

The As_4_S_3_ cage has idealised C_3v_ symmetry: one apical As atom bonded to three bridging S atoms (apical As-S: 2.230–2.234 Å), and three basal As atoms arranged in a nearly equilateral triangle via As-As bonds (2.460–2.480 Å; As-As-As angles 59.7–60.5°), with each basal As connected to the apical As through one of the bridging S atoms (basal As-S: 2.218–2.221 Å) [22,28]. Each basal As therefore carries one As-S bond and two As-As bonds, which gives the As_4_S_3_ cage a higher As-As bond density than any other stable As-S phase, three As-As bonds against two in realgar and none in orpiment [22,28]. The S–As(apical)-S angles span 98.3–98.8°, and the As-S-As bridging angles (apical to basal through each sulphur) are 105.0–105.4° [22]. The α-form (paradimorphite) cage has essentially identical bond distances, from a precise refinement on natural crystals: As-As 2.467–2.486 Å, As-S 2.216–2.236 Å [13], and near-identical angles (S-As(apical)-S: 97.8–98.0°; As-S-As: 105.7–106.1°; basal As-As-As: 59.9–60.2°) [28]. The polymorphic distinction between dimorphite and paradimorphite arises from a subtle difference in cage packing: the cage pseudo-threefold axis orients along [102] in dimorphite versus [101] in paradimorphite [13], with the larger unit-cell volume of paradimorphite (V = 750.4 Å^3^ vs. 735.4 Å^3^ for dimorphite) consistent with its stability at higher temperatures. The three basal As atoms each carry a stereochemically active lone pair directed outward from the cage, exposed electron-rich sites proposed as the structural basis for the gas-sensing behaviour of dimorphite thin films [15] and for a conductivity mechanism distinct from both orpiment and realgar. Paradimorphite (the high-T form) crystallises from the fumarolic vapour phase and is found at room temperature as pseudomorphs progressively converting to dimorphite, consistent with the thermodynamic instability of paradimorphite below ~130–149 °C [13]. Both minerals have a measured density of approximately 3.51 g/cm^3^ [13].

Ab initio quantum chemical calculations for As_4_S_3_ molecular cages and related As_4_S_n_ diversity have been reported by Kyono [29], who showed that the cage conformations observed in crystalline As-S phases correspond to energy minima on the molecular potential energy surface, providing a theoretical framework for understanding the stability of each polymorph. The lattice parameters of all the stable phases are collected in Table 3.

### 2.3. Electronic and Optical Properties

All three binary As-S compounds are moderate-to-wide bandgap semiconductors with optical bandgaps spanning ~2.4 to ~2.7 eV in crystalline form, placing their optical absorption edges in the visible range and consistent with their characteristic yellow-to-orange-red colours (Table 4). This visible bandgap, combined with wide mid-infrared transparency (the As-S system is transparent beyond 8–12 µm depending on composition), makes the As-S family unusual among inorganic semiconductors in combining visible photosensitivity with infrared transparency.

For crystalline As_2_S_3_, hybrid-functional calculations by Ulian et al. [23] give an indirect bandgap of 2.44 eV along Γ-A and a direct Γ-Γ gap of 2.63 eV, in agreement with earlier optical absorption edge determinations. The bands on either side of the gap are only weakly dispersed, so direct optical transitions are accessible alongside the indirect threshold, and the orbital decomposition places the upper valence bands on S 3p states with smaller As 4p and As 4s contributions, while the lower conduction bands are hybridised S 3p and As 4p with arsenic dominating [23]. The high-frequency dielectric tensor of the crystal, ε_∞_ = 8.265, 5.300 and 6.505 along x, y and z, corresponds to principal refractive indices of 2.87, 2.30 and 2.55 in the transparent region and to static values of 13.24, 5.89 and 9.96 once the lattice contribution is included [23]. The optical anisotropy of the crystal is therefore large, roughly 0.57 between the extreme principal indices, with the smallest value along the direction normal to the layers. The elastic tensor follows the same anisotropy, with C_11_ much larger than C_22_ and C_33_, and the Voigt–Reuss–Hill averages are modest, a bulk modulus of 28.5 GPa, a shear modulus of 19.2 GPa and a Young’s modulus of 46.9 GPa with a Poisson ratio of 0.225, values consistent with van der Waals stacking of covalent layers [23].

For realgar, single-crystal photoconductivity measurements by Street et al. [30] yielded an optical bandgap of 2.40 ± 0.05 eV. The same measurements resolved two independent transport channels within the one crystal, and they are described in Section 6.2 [30]. DFT calculations on realgar elastic and phonon properties under pressure, reported by Ulian et al. [31], predict a pressure-induced bandgap narrowing consistent with the experimentally observed colour change from orange-red to black under compression.

**Figure 4:**
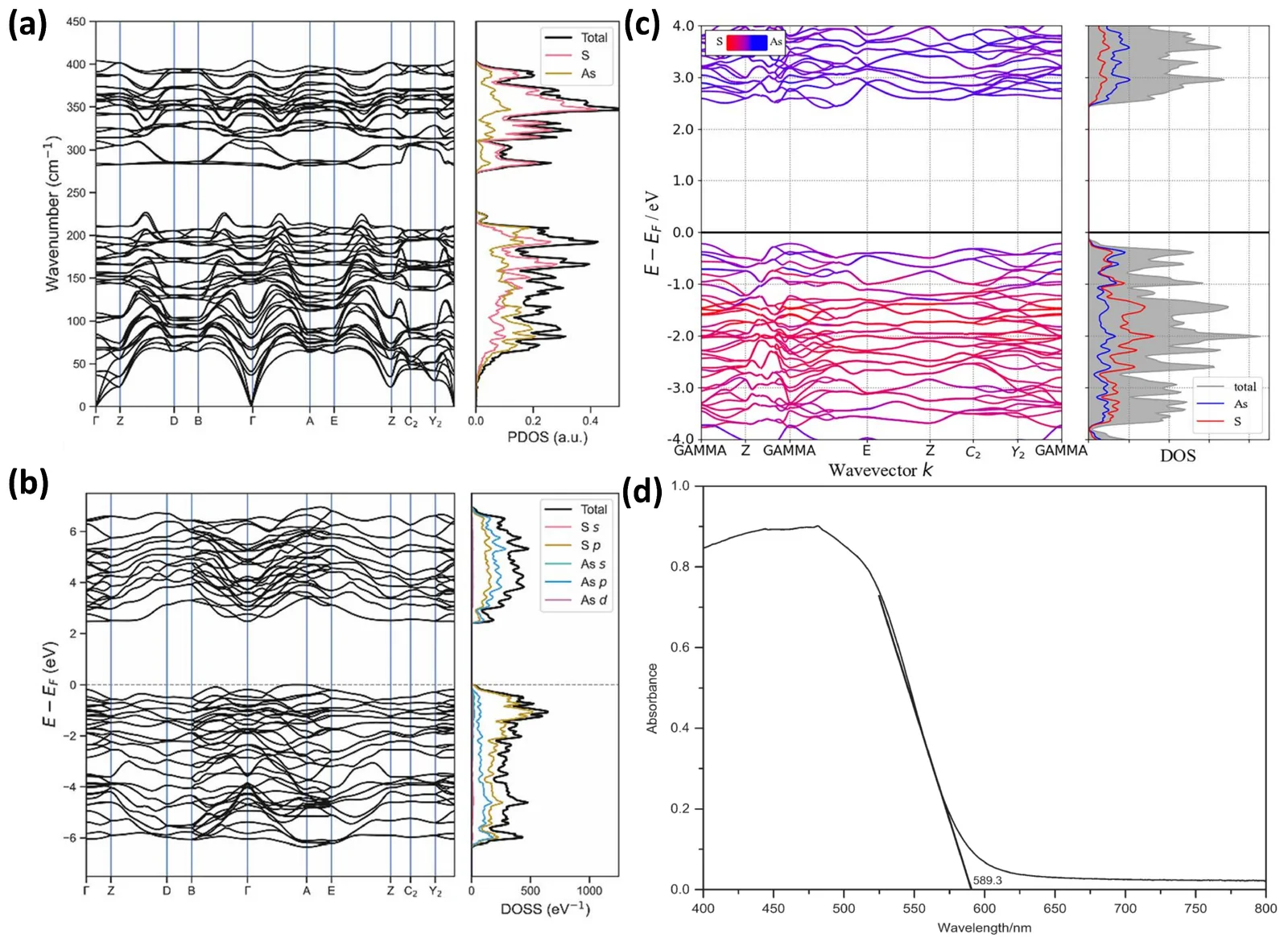
Computed and measured electronic structure of the crystalline As-S phases. (**a**) Phonon band structure and phonon density of states of bulk orpiment As_2_S_3_. (**b**) Electronic band structure and orbital-projected density of states of the same phase, giving an indirect gap of 2.44 eV along Γ-A and a direct Γ-Γ transition at 2.63 eV. (**c**) Electronic band structure and density of states of pararealgar, with a computed indirect gap of 2.62 eV corresponding to 473 nm. (**d**) Ultraviolet and visible absorption spectrum of realgar. Panels (**a**,**b**) reproduced from [23] with permission from the Royal Society of Chemistry, © 2025. Panels (**c**,**d**) reproduced from [32] with permission from John Wiley and Sons, © 2022.

Figure 4 gives the computed and measured electronic structure of the crystalline phases. The phonon dispersion and phonon density of states of orpiment in Figure 4a show no imaginary frequencies across the Brillouin zone, which confirms that the calculated structure is dynamically stable. The electronic band structure in Figure 4b shows the 2.44 eV indirect gap along Γ-A and the 2.63 eV direct transition at Γ, and the projected density of states assigns the upper valence bands to S 3p states with a smaller As 4p and As 4s contribution. Figure 4c gives the corresponding band structure of pararealgar, with a computed indirect gap of 2.62 eV, equivalent to 473 nm, and Figure 4d the measured ultraviolet and visible absorption spectrum of realgar. Panel d shows the absorption onset of realgar at 589.3 nm, equivalent to a band gap of 2.10 eV, which determines the photon energy required for the realgar to pararealgar conversion. Panel c gives the larger indirect gap of pararealgar, 2.62 eV or 473 nm, above which pararealgar converts further to uzonite under blue illumination [32].

For dimorphite, direct measurements of the crystalline bandgap are absent from the literature, representing one of the most significant open gaps in the characterisation of this phase. Optical data from composition-dependent thin-film studies across the As-S binary system [6] show that, as As content increases from As_35_S_65_ (~orpiment composition) to As_55_S_45_ (~dimorphite composition), the optical bandgap of amorphous films decreases from ~2.42 eV to ~1.87 eV, reflecting the progressive replacement of AsS_3/2_ pyramidal structural units by As_4_S_4_ and As_4_S_3_ cage-type units [6]. The bandgap of crystalline dimorphite (as opposed to amorphous As_4_S_3_ films) has not been reported.

For amorphous As_2_S_3_, by far the most studied phase in the thin-film literature, the transparency window is given in Section 5.3. The reference dispersion of the bulk glass and the index deficit of thin films against it are given in Section 5.2, and the thermo-optic coefficient in Section 5.4 [33]. The Tauc gap of the amorphous phase, its temperature dependence and its spread across the literature are given in Section 5.1 [34,35]. The non-linear refractive index n_2_ is approximately 3 × 10^−18^ m^2^W^−1^ (~100× silica), enabling efficient non-linear optical interactions in waveguides at modest power levels [3]. Amorphous As_2_S_3_ also exhibits pronounced photodarkening, treated in Section 4.7 and Section 5.6, which is the basis for holographic recording, waveguide writing and Bragg grating inscription in As_2_S_3_ films.

**Table 4:** Optical and electronic properties of the stable As-S binary phases.

Phase	Form	Bandgap (eV)	*n* (633 nm)	Transparency	Ref.
Orpiment As_2_S_3_	Crystalline	2.44 (indirect), 2.63 (Γ-Γ)	~2.7 (anisotropic)	Vis-MIR	[23]
Amorphous As_2_S_3_	Glassy	~2.3–2.4, Section 5.1	~2.4–2.55 (thin films)	0.57–11.8 µm	[34,35]
Realgar As_4_S_4_	Crystalline	2.40 ± 0.05	n.a.	Vis-NIR	[30]
Dimorphite As_4_S_3_	Crystalline	Not reported	n.a.	n.a.	n.a.
Amorphous As-S (~As_4_S_3_)	Glassy	~1.87–2.0	n.a.	n.a.	[6]

## 3. Synthesis and Deposition of As-S Thin Films

### 3.1. Bulk Synthesis and Target Preparation

Bulk synthesis of binary As-S compounds is performed by direct combination of elemental arsenic and sulphur in stoichiometric ratios, sealed under vacuum in fused quartz ampoules and heated to temperatures above the melting or sublimation point of the target compound. As_2_S_3_ bulk glass is produced by melt quenching from ~750–850 °C, yielding an amorphous solid with T_g_ ≈ 211 °C and T_x_ ≈ 220–250 °C suitable for optical fibre drawing [1]. Crystalline As_4_S_4_ is grown by chemical vapour transport using iodine as the transport agent (source zone ~350 °C, growth zone ~280 °C), yielding large transparent single crystals for optical and transport measurements [30]. Because dimorphite converts to paradimorphite between about 130 and 150 °C (Section 2.2.3), the cooling rate through that interval decides which polymorph is recovered, since the high-temperature form is kinetically trapped on rapid cooling. The congruent sublimation of As_4_S_3_ additionally allows vapour-phase crystal growth on cooler substrates directly from stoichiometric vapour, which is the laboratory analogue of natural fumarolic dimorphite formation [13].

For thin-film deposition, bulk-synthesised As-S compounds serve as sputtering targets or evaporation sources. Under ion bombardment a multicomponent target loses the component with the lower mass and the weaker surface binding preferentially, so the target surface enriches in the remaining component until an altered layer forms and the emitted flux returns to the bulk target composition [36]. Sulphur is nevertheless lost from sputtered As-S films, and for compounds carrying a volatile component the loss occurs at the growing film and in the gas phase and not only at the target [36]. No calibration of the effect specific to As-S targets has been published, so the target stoichiometry needed to reach a given film composition has to be established empirically. Table 5 in Section 3.7 summarises where each deposition route loses the source stoichiometry.

### 3.2. Thermal Evaporation

Thermal evaporation is the most widely used deposition technique for As-S thin films, particularly for As_2_S_3_. The method is straightforward. A resistively heated boat or crucible containing the As-S source material is heated under high vacuum (typically 10^−5^–10^−6^ Torr), generating a molecular beam that condenses on a substrate placed above the source. Although As_2_S_3_ sublimes congruently at equilibrium (Section 2.1), it evaporates incongruently in a practical thermal deposition, with preferential loss of sulphur from the molten source relative to the stoichiometric vapour, so the deposited film composition may differ from the source, particularly at high or unstable evaporation rates. Kondrat et al. measured this directly and found the film composition to depend on the vapour composition during evaporation and not on the source alone. Evaporation of As_40_S_60_ glass gave a film with an As to S ratio of 0.75 against 0.67 in the source, and As_45_S_55_ gave 0.96 against 0.82, both arsenic-enriched, while As_50_S_50_ gave 0.83 against 1.0 in the source, so the direction of the deviation is not the same at every composition [37]. The use of a pre-synthesised bulk As_2_S_3_ glass source and stable boat temperature minimises but does not eliminate stoichiometry drift. As-deposited films at room temperature are usually amorphous for any source composition. Crystallisation requires post-deposition annealing at temperatures appropriate to the target phase (Section 4). The optical bandgap and Urbach energy of thermally evaporated As_2_S_3_ films are given in Section 5.1 and the refractive index in Section 5.2 [2,8]. The film thickness is easily controlled by deposition rate (monitored by quartz crystal microbalance) and deposition time.

An important distinction applies for As_4_S_3_ evaporation because As_4_S_3_ sublimes congruently, evaporating as intact As_4_S_3_ molecular units without decomposition, and the stoichiometry transfer from source to film is substantially more reliable than for As_2_S_3_ [5]. This makes thermal evaporation of dimorphite relatively straightforward in terms of composition control. The first thin-film As_4_S_3_ deposition by thermal vacuum evaporation was reported by Tsiulyanu et al. in 1996 [14], who showed that films evaporated from an As_4_S_3_ source exhibit an optical absorption edge near 2.5 eV and photoconductivity with spectral maxima at 2.9 and 4.28 eV. XRD analysis revealed coexistence of α- and β-dimorphite microcrystalline inclusions even in nominally room-temperature depositions, and the I_β_/I_α_ intensity ratio increased with increasing evaporation rate, establishing that phase composition in thermally evaporated As_4_S_3_ films is controlled by growth velocity [14]. Substrate temperature influences in the same direction as growth velocity. Deposition at 20 °C shifts the balance towards dimorphite and deposition at 60 °C towards paradimorphite, well below the bulk inversion temperature near 149 °C, so the high-temperature polymorph is kinetically accessible whenever the arriving cages are given time to order [14]. Both polymorphs are therefore reachable from the same source material, and the deposition recipe has to control which polymorph nucleates as well as whether the transition is crossed during growth or during cooling. Growth velocity also determines film morphology. Films deposited at 0.2 µm/h are fibrillar and porous, with fibrils about 2 µm long and 0.5 µm in diameter, while films deposited at 5.0 µm/h are smooth and compact [15]. The consequences of that morphology for charge transport and for gas sensing are treated in Section 6.3 and Section 6.4.

### 3.3. RF Magnetron Sputtering

RF magnetron sputtering offers better step coverage, substrate adhesion and process reproducibility than thermal evaporation, and it is compatible with industrial deposition platforms. For As-S films, deposition proceeds from pressed powder or sintered targets in an argon atmosphere [38]. The preferential loss of sulphur from the target and from the growing film and the absence of any calibration specific to As-S targets are described in Section 3.1 [36]. The magnitude of the loss depends on argon pressure, discharge power and target to substrate distance, so the film composition has to be verified for every parameter set (Section 4.1). Reactive deposition in a sulphur-bearing atmosphere has not been reported for As-S.

The Urbach absorption edge of As_2_S_3_ films widens with the structural disorder frozen in during growth, and the sputtered network is the more disordered of the two, which is attributed to the higher kinetic energy of the arriving sputtered species relative to the thermal evaporation flux [39]. Sputtered films are not optically inferior for that reason. Magnetron-sputtered a-As_2_S_3_ resists ambient oxidation better than thermally evaporated films of the same composition, showing no detectable As_2_O_3_ crystallites after air exposure, and its waveguide losses are given in Section 5.3 [38]. Both methods yield amorphous films on room-temperature substrates.

For As_4_S_3_, no radio-frequency magnetron sputtering study has been published. The congruent sublimation that makes thermal evaporation straightforward for this phase [5] gives no equivalent advantage in sputtering, where material is removed by momentum transfer and the sulphur loss described in Section 3.1 applies. Two questions therefore remain open, which target composition and deposition conditions place the film at As:S near 57:43, and whether a sputtered layer arrives amorphous or, as for thermal evaporation [14], already microcrystalline, since that decides whether the processing problem is crystallisation or polymorph selection.

### 3.4. Pulsed Laser Deposition

Pulsed laser deposition (PLD) transfers material from a target to a substrate by ablation with high-fluence laser pulses, producing a stoichiometric plasma plume that condenses on the substrate. PLD offers essentially stoichiometric transfer of the target composition to the film, avoiding the preferential sputtering/evaporation problem. The technique is relevant to As-S because sulphur is lost during thermal evaporation.

The most thorough PLD study of As_2_S_3_ films for waveguide applications used ultrafast PLD with picosecond-range Nd:YAG pulses at 532 nm [40]. Zakery et al. compared two laser regimes, Q-switched mode-locked (QSML, ~10-kHz trains) and pure mode-locked (ML, 76-MHz repetition rate, 70–80 nJ/pulse). In the ML regime, particulates were completely eliminated, yielding atomically smooth films with roughness of 0.42 nm over 15 × 15 µm^2^. The QSML regime still produced some particulates. Compositional analysis showed films are slightly S-deficient (up to 1.5 at. % vs. target), consistent with residual thermal evaporation component at 532 nm where the As_2_S_3_ absorption depth (~2 µm) slightly exceeds the ablation front penetration. Films up to 5 µm thick were deposited at 2 nm/s on fused-silica or oxidised-Si substrates in a 3 × 10^−7^ Torr vacuum.

The refractive index of these films is given in Section 5.2, the photoinduced index change in Section 5.6 and the optical bandgap in Section 5.1 [40]. Annealing at 150 °C for 2 h has the opposite effect (Δn ≈ −0.02), and rapid thermal annealing of 5 µm thick films invariably causes cracking, a consequence of the large thermal expansion mismatch with fused-silica substrates. As-deposited ML films are therefore used directly for waveguide fabrication without annealing.

Waveguides were written by scanning a focused 514/532 nm beam across the film surface (photodarkening-based direct writing), producing channel structures 3.5 µm × 2.5 µm that are single-mode at 1300 and 1550 nm. A surface photodecomposition problem (growth of As and S crystals upon daylight exposure) was mitigated by a PMMA overcoat (~0.8 µm), which simultaneously increased the damage threshold for laser writing by two orders of magnitude. The propagation losses of these waveguides are given in Section 5.3 and their non-linear coefficients in Section 5.5 [40].

Mihai et al. deposited 2 µm thick amorphous As_2_S_3_ films by PLD (KrF, 248 nm, 1.5 J/cm^2^, 10 Hz, 11,000 pulses) on glass substrates and used them for near-field femtosecond laser structuring, which produced photoexpansion below the modification threshold and photoevaporation above it at the submicrometre scale [41].

### 3.5. Plasma-Enhanced Chemical Vapour Deposition

PECVD deposits As-S films with continuously variable composition across the full binary range by adjusting the plasma power and precursor ratios. Mochalov et al. [7] and Nezhdanov et al. [6] developed a PECVD approach using elemental As and S precursors in low-temperature Ar plasma (40 MHz RF), producing amorphous As-S films with As content from 35 to 60 at. % (all films amorphous as-deposited, regardless of composition or thickness [7]). The wider IR transparency window of PECVD films (to ~18 µm vs. ~12 µm for thermally evaporated As_2_S_3_) reflects the absence of As-O functional groups and reduced network disorder and was measured on a 5 µm PECVD film against a 0.5 µm evaporated film, so the wider window was obtained on the thicker of the two [7]. As the composition shifts from As_35_S_65_ towards As_55_S_45_, the dominant structural units transition from AsS_3/2_ trigonal pyramids to As_4_S_4_ and As_4_S_3_ cage-like configurations, a structural evolution tracked by Raman spectroscopy and by ab initio quantum chemical calculations, with the band assignments given in Section 4.2 [6,7]. PECVD provides access to the full As-S composition space in a single deposition system without changing targets, enabling systematic studies of structure–property relationships as a function of stoichiometry. The optical bandgap varies continuously from ~2.42 eV (near orpiment composition) to ~1.87 eV (near dimorphite composition) [6], covering the widest composition-tunable bandgap range reported for any As-S deposition method. Ex situ laser irradiation (473 nm) of PECVD-grown films produces micro- and nanocrystalline inclusions in the amorphous matrix, and the photoluminescence of those inclusions is treated in Section 6.5 [6].

PECVD synthesis at compositions near As_55_S_45_ produces amorphous films containing As_4_S_3_-type cage fragments as structural units (confirmed by mass spectroscopy analysis of the plasma species [7] and by Raman spectroscopy [6]) but does not produce crystalline dimorphite films without additional post-deposition processing. The PECVD-derived material is therefore best described as a dimorphite-stoichiometry amorphous chalcogenide, not a phase-pure dimorphite film, and crystallisation would require post-deposition annealing, which has not been reported for this composition.

### 3.6. Solution Processing

Solution-phase deposition of As-S films has been explored as an alternative low-cost route that eliminates the need for vacuum equipment. Two main approaches have been reported: diamine-based spin-coating, in which As_2_S_3_ (and As_2_Se_3_) is dissolved in an organic diamine solvent, spin-coated, and thermally decomposed to yield amorphous chalcogenide films [42], and chemical bath deposition (CBD), in which As_2_S_3_ films are deposited from aqueous acidic solutions of As_2_O_3_ and a sulphur source (typically thioacetamide) at room temperature [43]. Solution processing by dimensional reduction, in which the extended framework is broken into soluble molecular units and reassembled on heating, places the diamine route alongside hydrazine as a second solubilising medium, and amorphous As_2_S_3_ and As_2_Se_3_ films have been deposited in this way [44]. For CBD, the thioacetamide-bath approach of Mane et al. (As_2_O_3_ + Na_2_EDTA complexing agent + thioacetamide, pH 2.5, 6 °C) produced monoclinic nanocrystalline As_2_S_3_ films [43]. Deposition times of 4 to 20 h give thicknesses of 185 to 520 nm and mean grain sizes of 11 to 38 nm [45]. The optical bandgaps of this film series are given in Section 5.1 and the resistivities and activation energies in Section 6.1. The direct optical bandgap was 2.20 eV, and electrical resistivity was ~0.51 × 10^6^ Ω·cm at 310 K with an activation energy of 0.15 eV. The resistivity–temperature curve showed two distinct slopes, attributed to the α→β As_2_S_3_ phase transition [43]. CBD films tend to be nanocrystalline and porous with higher defect density compared to vacuum-deposited films, resulting in reduced photosensitivity. A fundamental limitation of solution-processed chalcogenide films is incomplete solvent removal. Infrared spectroscopy detects residual N-H and C-H absorption even after baking at 100–200 °C, and the waveguide propagation losses that follow from it are given in Section 5.3 [3]. Solution-processed films are therefore of limited interest for high-performance photonic applications but may be suitable for sensor applications where large areas and low cost are priorities. To date, solution deposition of As_4_S_4_ or As_4_S_3_ compositions has not been reported.

**Figure 5:**
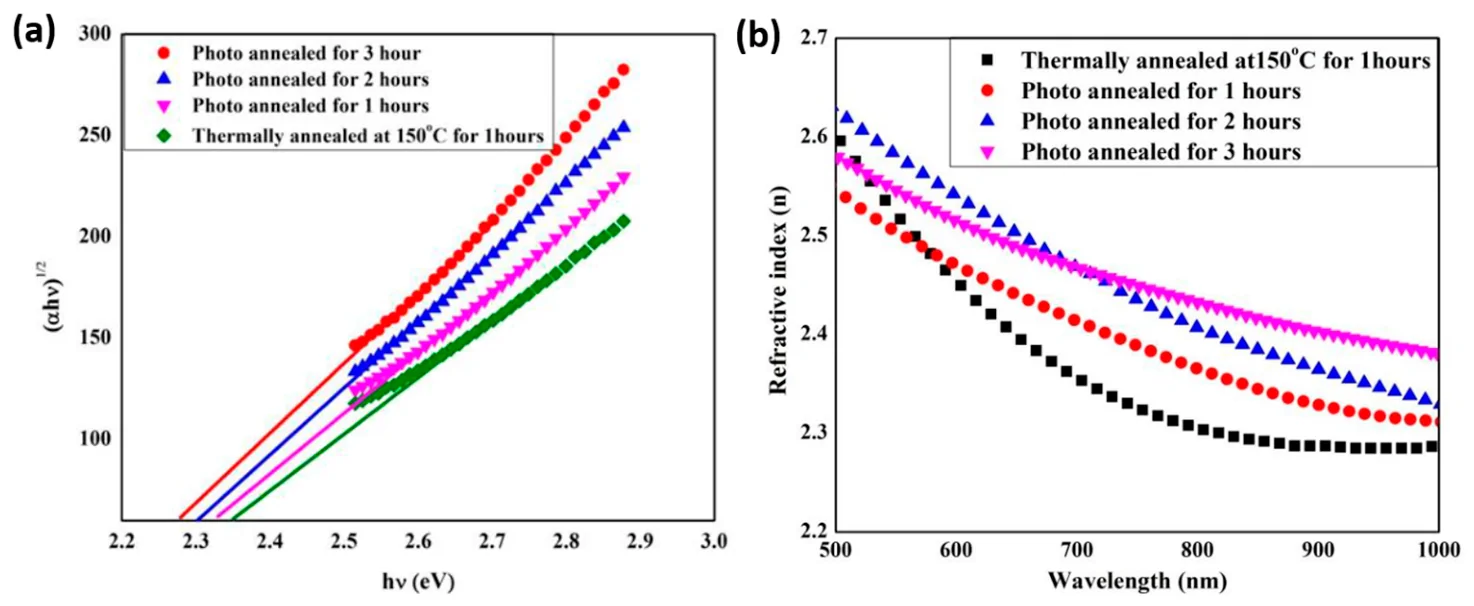
Optical properties of As_2_S_3_ films by deposition route. (**a**) Tauc plots, (αhν)^1/2^ against photon energy, for a thermally deposited film annealed at 150 °C for 1 h and for solution-derived films photoannealed for 1, 2 and 3 h. (**b**) Refractive index against wavelength for the same films, obtained by the Swanepoel method. Reproduced from [46] under a Creative Commons Attribution 3.0 Licence, © 2022 the authors.

Figure 5 compares the two routes directly. Figure 5a gives Tauc plots for a thermally deposited film annealed at 150 °C for 1 h and for solution-derived films photoannealed for 1, 2 and 3 h, from which the optical bandgap is 2.35 eV for the thermally deposited film and falls from 2.32 to 2.27 eV as the photoannealing of the solution-derived films is extended. Figure 5b shows the corresponding refractive index dispersion obtained by the Swanepoel method, giving 2.30 for the thermally deposited film against 2.32 to 2.40 for the solution-derived films, an increase attributed to densification as residual solvent is removed. Both sets of films follow normal dispersion across the measured range [46].

### 3.7. Comparison of Deposition Methods

Table 5 summarises the main characteristics of As-S thin-film deposition methods. The choice of method involves trade-offs between composition control, structural quality, surface roughness, scalability, and cost. Among the methods compared in Table 5, thermal evaporation and PLD give the highest surface quality for As_2_S_3_. For As_4_S_3_, thermal evaporation exploits the congruent sublimation advantage for stoichiometry transfer. RF sputtering has not been applied to this composition (Section 3.3), so its scalability advantage for As-S remains untested. PECVD is the only method that varies composition continuously across the binary, and it requires more complex gas handling. Solution processing is lowest cost but gives the poorest film quality.

**Table 5:** Comparison of As-S thin-film deposition methods.

Method	Stoichiometry Control	Film Quality (n, Roughness)	Scalability	Refs.
Thermal evaporation	Moderate, incongruent for As_2_S_3_ and congruent for As_4_S_3_	Excellent	Low to medium	[2,14]
RF magnetron sputtering	Sulphur lost from the film, no published calibration for As-S targets	Good, slightly more disordered than evaporated	High	[6,36,38]
PLD	Excellent, stoichiometric transfer with minor S deficiency of about 1.5 at. %	Excellent in the ultrafast ML regime, rms 0.42 nm, conventional PLD produces particulates	Low	[40,41]
PECVD	Excellent, continuous composition tuning	Good	Medium	[6,7]
Solution (CBD)	Limited, films nanocrystalline and monoclinic, grain size 11 to 38 nm	Moderate, high porosity and high defect density	Very high	[43,45]

**Figure 6:**
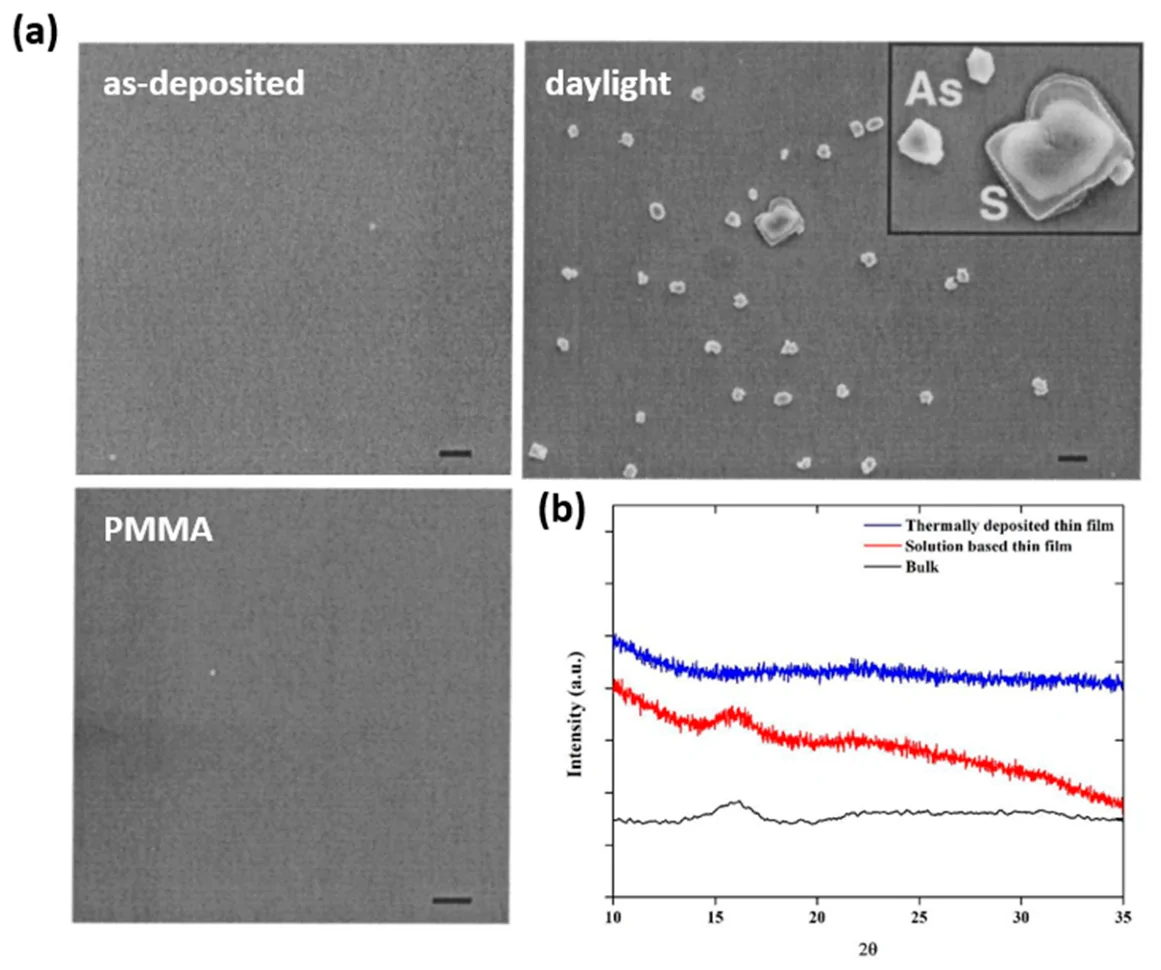
The deposition route determines the structure and the surface of the film. (**a**) Scanning electron micrographs of ultrafast-laser-deposited As_2_S_3_, as deposited, after several days under daylight showing arsenic and sulphur crystal growth, and after coating with PMMA, which mitigated the crystal growth. (**b**) X-ray diffraction patterns of bulk As_2_S_3_, of a thermally deposited film and of a solution-derived film. Panel **a** reprinted from [40] with permission from the Optical Society of America, © 2003. Panel **b** reproduced from [46] under a Creative Commons Attribution 3.0 Licence, © 2022 the authors.

Representative structural and morphological data are compared in Figure 6. Figure 6a shows the surface of ultrafast-laser-deposited As_2_S_3_ as deposited, after several days of daylight exposure and after encapsulation in PMMA, and documents the arsenic and sulphur surface crystallisation that limits the lifetime of unprotected films. Figure 6b gives the X-ray diffraction patterns of bulk As_2_S_3_, of a thermally deposited film and of a solution-derived film, none of which shows sharp reflections, so the solution route yields the same amorphous short-range order as the vacuum route and as the bulk glass [46].

## 4. Structural Characterisation of As-S Thin Films

Reliable structural characterisation of As-S thin films requires a multitechnique approach because the three phases, amorphous network, cage-molecule crystallites, and layered crystalline polymorphs, present challenges that no single technique resolves completely. Raman spectroscopy identifies molecular structural units and discriminates phases non-destructively, X-ray diffraction in grazing-incidence geometry (GIXRD) confirms long-range crystalline order and quantifies phase fractions, atomic force microscopy (AFM) and scanning electron microscopy (SEM) characterise surface morphology, Rutherford backscattering spectrometry (RBS) and energy-dispersive X-ray spectroscopy (EDX) establish the film stoichiometry, differential scanning calorimetry (DSC) maps the thermal stability, and extended X-ray absorption fine structure (EXAFS) probes medium-range order in the amorphous state. The following subsections review the application of each technique to As-S thin films, with particular attention to the phase-specific signatures that distinguish As_2_S_3_, As_4_S_4_, and As_4_S_3_ phases and to the photoinduced structural transformations that underlie the photonic applications of these materials.

### 4.1. Composition Verification by RBS and EDX

Accurate knowledge of film composition is a prerequisite for interpreting any structural or optical measurement, because the three As-S phases differ by only 7–17 at. % in arsenic content (As_2_S_3_: 40 at. %, As_4_S_4_: 50 at. %, As_4_S_3_: 57.1 at. %) and can coexist in off-stoichiometry films. Rutherford backscattering spectrometry is the primary quantitative technique for As-S thin films. It provides absolute atomic areal densities without reference standards, and the As (Z = 33) and S (Z = 16) signals are well-resolved in energy on light substrates such as Si, SiO_2_ and glass. Zakery et al. used it together with energy-dispersive X-ray analysis on pulsed-laser-deposited As_2_S_3_, reaching an accuracy of order 0.1 at. % against a target accurate to within 1 at. %, which is how the sulphur deficiency reported in Section 3.4 was established [40]. It has not been applied to the arsenic-rich compositions.

Energy-dispersive X-ray spectroscopy coupled to scanning electron microscopy is more accessible but inherently semiquantitative. The As L_α_ (1.282 keV) and S K_α_ (2.307 keV) lines are well-separated at standard beam energies, but the excitation volume at 20 kV extends 1–2 µm into the sample, so films thinner than ~500 nm are contaminated by substrate signal and require thin-film correction factors. For thicker films, EDX determines As:S ratios to approximately ±2–3 at. % relative to appropriate standards. Mane et al. used EDX to characterise chemical-bath-deposited As_2_S_3_ films and detected oxygen incorporation from the aqueous deposition medium, a systematic contamination absent in vacuum-deposited films [43]. A combined RBS+EDX approach is recommended in practice. RBS delivers the quantitative ground truth for stoichiometry, while EDX enables rapid spatial mapping of compositional uniformity across the substrate.

### 4.2. Raman Spectroscopy, Structural Unit Identification and Phase Discrimination

Raman spectroscopy is the most widely used structural characterisation tool for As-S thin films because it is non-destructive, sensitive to short-range order in amorphous materials, requires no sample preparation, and unambiguously distinguishes all As-S phases, amorphous, orpiment, realgar, pararealgar, and dimorphite, from one another by their molecular vibrational fingerprints.

**Figure 7:**
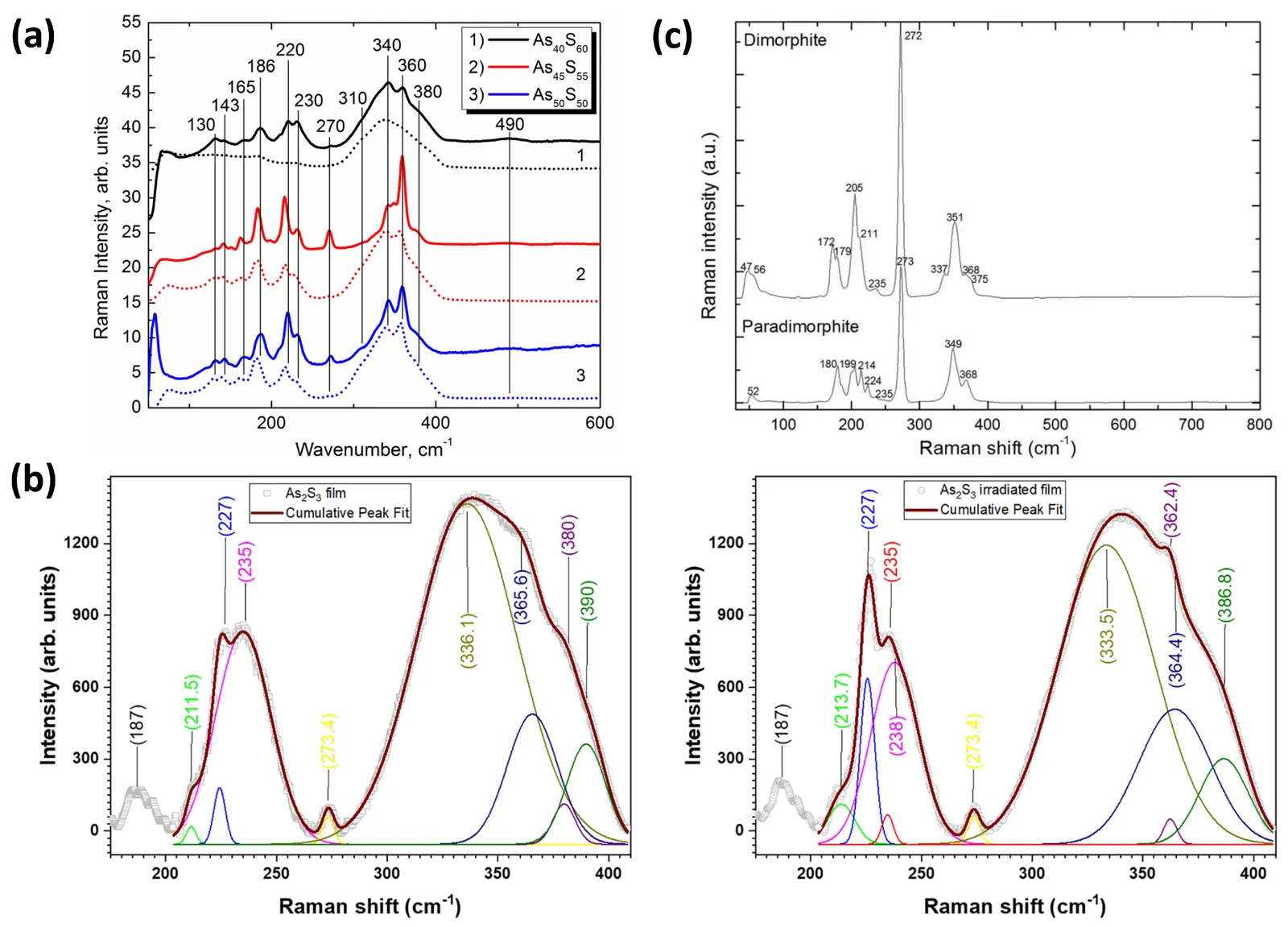
Raman of the As-S phases, across composition, under irradiation and against the crystalline references. (**a**) As_40_S_60_, As_45_S_55_ and As_50_S_50_ target glasses, dotted lines, and the corresponding thermally evaporated films, solid lines. (**b**) An As_2_S_3_ film before and after single-pulse femtosecond near-field patterning, recorded at 632.8 nm over 175 to 410 cm^−1^, each spectrum deconvoluted with eight Gaussians. (**c**) Reference spectra of paradimorphite and dimorphite recorded on bulk crystals, where the 337 cm^−1^ band is characteristic of dimorphite and the 224 cm^−1^ band of paradimorphite. Band positions and assignments are collected in Table 6. Panel (**a**) reproduced from [37] under a Creative Commons Attribution 4.0 International Licence, © 2017 the authors. Panel (**b**) reproduced from [61] with permission from Elsevier, © 2026. Panel (**c**) reproduced from [13] with permission from Cambridge University Press on behalf of the Mineralogical Society of Great Britain and Ireland, © 2022.

The Raman and infrared spectra of a-As_2_S_3_ carry six features, collected in Table 6 and shown for thermally evaporated films in Figure 7a. A broad asymmetric band near 340 cm^−1^ dominates the polarised Raman spectrum. The strongest infrared mode lies at 308 cm^−1^, with a shoulder at 345 cm^−1^. Weaker polarised features appear at 231 cm^−1^ and between 380 and 395 cm^−1^, and a band at 491 cm^−1^ appears when sulphur is in excess.

The 340 cm^−1^ band is the symmetric stretching vibration of the AsS_3/2_ trigonal pyramid, the building block of the disordered chalcogenide network [47,48]. Lucovsky et al. placed the remaining assignments on a structural basis by comparing amorphous As_2_O_3_ with its two crystalline polymorphs, claudetite, a layer crystal similar to orpiment, and arsenolite, a molecular crystal built from As_4_O_6_, and extending the comparison to amorphous As_2_S_3_ and As_2_Se_3_ [49]. In that framework the 340 cm^−1^ polarised mode is a layer-type sulphur breathing mode of B2 symmetry belonging to the claudetite and orpiment topology, and the 308 cm^−1^ infrared mode is a combined sulphur and arsenic displacement. The 231 cm^−1^ and 380 to 395 cm^−1^ features are B3+B2 and B2−B3 modes of regions carrying an As_4_S_6_-like molecular topology [49].

Amorphous As_2_S_3_ is therefore predominantly layer-like, with a dihedral angle distribution peaked at the angles characteristic of the layer structure and not randomly distributed. The molecular As_4_S_6_-type regions contribute weakly, and less than the analogous As_4_O_6_ regions in the isoelectronic oxide, where arsenolite is a stable compound [49]. The 491 cm^−1^ band is the S-S homopolar stretch, and its diagnostic value for sulphur excess is not in dispute, which makes it a sensitive probe of departure from stoichiometry [47,50].

The assignment of the 231 cm^−1^ band is the one point in dispute. It is commonly attributed to As-As homopolar bonds, and Lucovsky et al. argued against that attribution for stoichiometric glass on two grounds, that the band appears in sulphur-rich glasses where As-As bonds are absent, and that arsenic-rich glasses which do contain As-As bonds show additional features not present in the stoichiometric glass [49]. Against that, the band strengthens on photodarkening (Section 4.7 and Section 5) and in arsenic-rich films, both consistent with the As-As assignment under those conditions. The same enhancement follows a single femtosecond pulse. Jipa et al. deconvoluted the Raman spectrum of an As_2_S_3_ film before and after near-field patterning with one 775 nm pulse and found the band, at 227 cm^−1^ in their films, to rise about fourfold to the intensity of the neighbouring 235 cm^−1^ feature, while the 340 cm^−1^ network band remained dominant, so the irradiation reorders the short-range structure without crystallising the film [51]. They assign the enhanced band to As_4_S_4_-like units and not to network As-As bonds, which places the same feature on the molecular side of the argument in a film that has been driven far from equilibrium. Georgiev et al. resolved the 180 to 230 cm^−1^ region further, assigning modes near 183 and 221 cm^−1^ to a discrete As_4_S_4_ monomer with the realgar motif and modes near 195, 206 and 227 cm^−1^ to a polymerised arsenic-rich configuration, and following their appearance with composition, the 232 cm^−1^ feature from about x = 0.35 and the 221 cm^−1^ feature from about x = 0.4 in As_x_S_1−x_ [52]. The stoichiometric glass is intrinsically phase separated into small arsenic-rich and large sulphur-rich clusters, which places the band on neither side of the earlier argument but assigns it to a distinct nanophase. The dispute is therefore not only about the origin of the weak feature near 231 cm^−1^ in undisturbed stoichiometric glass but about whether that glass is homogeneous at the nanometre scale at all.

A particularly important observation for as-deposited films is that freshly thermally evaporated As-S films contain substantial As_4_S_4_ molecular fragments, significantly more than equilibrium bulk glasses of the same composition. FT-Raman measurements at 1064 nm (chosen to minimise photodarkening during measurement) by Frumar et al. [50] quantified As_4_S_4_ content in As_42_S_58_ films and glasses. Freshly evaporated films contain ≥20 mol. % As_4_S_4_, while bulk As_42_S_58_ glass contains only ~10 mol. %. The excess As_4_S_4_ in evaporated films arises from thermal dissociation during sublimation: 2As_2_S_3_ → As_4_S_4_ + S_2_. This molecular fraction is reduced by both visible-light exposure and thermal annealing. Illumination simultaneously increases the 235 cm^−1^ band assigned to As-As bonds incorporated in the amorphous network (distinct from the cage As-As modes at 185–194 cm^−1^ in crystalline As_4_S_4_). The photoinduced redshift of the absorption edge is connected to this increase in network As-As bond density and not to As_4_S_4_ dissolution, consistent with the bond-statistics equilibrium 2|As-S| ⇌ |As-As| + |S-S| shifting rightward upon illumination and leftward upon annealing [50]. This model provides a microscopic basis for photodarkening in as-evaporated As-S films.

The Raman spectrum of a-As_2_S_3_ probes the bulk of the film and not only its surface, and it is consistent between thermally evaporated and RF-sputtered films, with slightly broader linewidths in sputtered films that reflect the higher structural disorder also seen in the Urbach edge [39]. In the low-frequency region the spectra of As_x_S_100−x_ glasses carry a boson peak whose intensity has been assigned to a nanocluster contribution to the medium-range structure [53].

Crystallisation of amorphous As_2_S_3_, by thermal annealing above T_x_ ≈ 220 °C or under certain illumination conditions, produces orpiment, whose Raman spectrum displays sharp, resolved peaks near 290, 310, and 356 cm^−1^. The line-narrowing and the ~16 cm^−1^ blueshift of the main AsS_3_ peak (from the amorphous 340 to the crystalline 356 cm^−1^) constitute an unambiguous crystallisation marker detectable even at crystallised volume fractions below 5–10%, making Raman more sensitive than XRD for detecting incipient crystallisation. In practice, the ratio of the crystalline 356 cm^−1^ peak integrated area to the amorphous 340 cm^−1^ residual halo provides a semiquantitative crystallinity index routinely used to monitor annealing progress [54].

Realgar has a characteristic Raman signature dominated by a strong peak at 353 cm^−1^ (A_g_ symmetry, As-S cage stretching), accompanied by modes at 185 and 194 cm^−1^ (As-As stretching within the cage), 250 cm^−1^ (S-As-S bending), and a cluster of peaks between 371 and 403 cm^−1^ [54,55]. The As-As stretching modes at 185–194 cm^−1^ are diagnostic of the cage topology and clearly absent in the orpiment spectrum. Pararealgar, the photoproduct of realgar under visible-light illumination, has a distinctly different Raman spectrum despite having the same As_4_S_4_ stoichiometry. Trentelman et al. [26] reported the first Raman spectrum of pararealgar and of the intermediate χ phase. The pararealgar spectrum is characterised by a very strong doublet near 230 cm^−1^ and four resolved peaks around 330 cm^−1^, at 332, 345 and 353 cm^−1^, all very strong, and at 363 cm^−1^ of medium intensity [26]. Against realgar, pararealgar shows more bands of medium to strong intensity, consistent with its reduced molecular symmetry, a pseudo-mirror plane only and three inequivalent As environments [26]. The two isomers can be unambiguously discriminated in mixtures, enabling in situ tracking of the photoinduced conversion, and at 632.8 nm excitation the realgar spectrum shows a dominant 354 cm^−1^ peak, the pararealgar spectrum the 230/236 cm^−1^ doublet and 345/353 cm^−1^ group [26]. The χ phase intermediate in this transformation exhibits a distinctive Raman spectrum with a strong peak at 361 cm^−1^ and a weaker band at 186 cm^−1^, clearly different from both realgar (354 cm^−1^ dominant) and pararealgar, and can be identified in mixed spectra even when partially overlapped by pararealgar bands [26]. The χ phase also has an expanded monoclinic unit cell (a = 9.758, b = 9.522, c = 9.074 Å, β = 100.84°) [24].

The two polymorphs of dimorphite and paradimorphite are separated by Raman even though their cages are identical. β-dimorphite (the low-temperature polymorph) shows a distinctive band at 337 cm^−1^ that is absent in α-dimorphite, while α-dimorphite has a characteristic band near 224 cm^−1^ not present in β [13]. The dominant cage-mode cluster (336–375 cm^−1^ region in β and 349–368 cm^−1^ in α) differs in peak positions and relative intensities. In the infrared and Raman lattice-mode region below 100 cm^−1^, β-dimorphite shows a 47–56 cm^−1^ doublet while α-dimorphite shows a single band at 52 cm^−1^ [13]. These signatures are the candidate non-destructive polymorph diagnostics for deposited thin films, although their detection in films has not yet been tested (see below).

In thermally evaporated As-rich As-S films, as-deposited samples already contain molecular cage fragments, notably As_4_S_4_ and As_4_S_3_, even at stoichiometric As_40_S_60_ (As_2_S_3_) composition [56]. Their Raman spectra display unusually narrow peaks in the 100–400 cm^−1^ region compared to bulk glass, confirming the presence of distinct As-rich cage fragments coexisting with the disordered network [56]. A band near 271 cm^−1^, assigned to As_4_S_3_ cage-type stretching modes, is detectable in virgin thermally evaporated As-S films and its intensity is selectively reduced under superbandgap UV irradiation, interpreted as progressive photovaporisation of cage molecules from the film surface (see Section 4.7) [56]. In PECVD films spanning the As-rich composition range (As_35_S_65_ to As_55_S_45_), the 271 cm^−1^ As_4_S_3_ cage band grows systematically with increasing arsenic content while the AsS_3_ pyramidal peak at 340 cm^−1^ diminishes and the As-As band at 231 cm^−1^ intensifies, tracking the progressive transition from network to cage-dominated short-range order [6]. Near the dimorphite stoichiometry (As_55_S_45_), focused 473 nm laser illumination at low powers (photocrystallisation regime) produces a transition from the broad As_4_S_3_ cage amorphous signature to sharper cage modes indicative of crystalline dimorphite [6]. PECVD-deposited As-S films from the same group additionally exhibit a band near 650 cm^−1^ that is absent in thermally evaporated films. Quantum-chemical calculations assign this band to cycled two-dimensional As-S structural units formed in the plasma discharge [7]. A complete Raman mode assignment for crystalline dimorphite and paradimorphite in thin-film form has not yet been published. The mineral spectra of Campostrini et al. [13] supply the reference bands, but no film study has tested whether those bands remain resolvable at thin-film signal levels. Those reference spectra are reproduced in Figure 7c.

Table 6 lists the diagnostic Raman bands of each structural unit and phase found in As-S films, together with the surface arsenolite formed on photo-oxidation, and gives the deposition route in which each has been observed.

**Table 6:** Principal Raman bands in As-S thin films by structural unit and phase.

Structural Unit/Phase	Key Raman Bands (cm^−1^)	Assignment	Film Deposition	Refs.
a-As_2_S_3_ (amorphous network)	340 (broad, main, P); 308 (IR, strong); 231 * (P); 345 (IR, sh.); 390–395 (sh., P); 491 †	B2 layer-type S breathing (network, claudetite topology); S2−S3 IR mode; B3+B2 (As_4_S_6_-like topology) or As-As ‡; B2−B3; S-S †	Thermal evaporation; RF sputtering	[47,49]
As_2_S_3_ crystalline (orpiment)	290; 310; 356 (sharp, main)	AsS_3_ modes with crystal-field splitting	Annealed evaporation/sputter	[54]
As_4_S_4_ (realgar)	353 (strong); 185; 194; 250; 371–403	As-S cage stretch; As-As stretch; S-As-S bend	Sputtered; annealed	[54,55]
As_4_S_4_ (pararealgar)	230 (very strong), 236 (very strong); 332 (very strong), 345 (very strong), 353 (very strong), 363 (medium); χ-phase: 361 (very strong), 186 (medium)	Reduced symmetry cage (pseudo-mirror)	Phototransformed realgar	[24,26]
As-rich amorphous (~As_40_S_60_-As_55_S_45_)	~271 (As_4_S_3_ cage); 340 (residual); 231 (enhanced)	Cage-type units present even at stoichiometric composition; grow with As content	Thermal evaporation (cage fragments in as-deposited films); PECVD As-rich	[6,56]
As_2_O_3_ (arsenolite, photo-oxidation)	264; 366; 555	As_2_O_3_ molecular cage modes	UV-exposed a-As_2_S_3_ surface	[57,58]

* P = polarised Raman, IR = infrared active, sh. = shoulder, and band intensities are vs = very strong, s = strong, m = medium, w = weak. † The 491 cm^−1^ S-S stretch is weak in stoichiometric glass and is enhanced by sulphur excess or by photodarkening. ‡ Three assignments are in circulation for the band near 231 cm^−1^. Lucovsky et al. assign it to As_4_S_6_-like molecular topology and not to As-As bonds in stoichiometric glass [49]. The As-As assignment [18] is supported by photodarkening and by arsenic-rich compositions, where additional As-As features appear. Georgiev et al. assign the 227 to 232 cm^−1^ region to a polymerised arsenic-rich configuration and the 221 cm^−1^ band to a discrete As_4_S_4_ monomer, on the basis that the stoichiometric glass is phase separated at the nanometre scale [59]. Reference Raman spectra for the crystalline phases listed here are available in the RRUFF database [60].

Figure 7 gives the reference spectra behind these assignments. Figure 7a compares the target glasses As_40_S_60_, As_45_S_55_ and As_50_S_50_ with the films thermally evaporated from them, which shows how the Raman signature changes with composition and between a glass and the film grown from it. Figure 7b gives an As_2_S_3_ film before and after single-pulse femtosecond near-field patterning, where the 227 cm^−1^ band grows about fourfold against the 235 cm^−1^ feature while the 336 cm^−1^ network band is unchanged in position and shape, and a sulphur–sulphur band near 211 cm^−1^ persists. Figure 7c gives the two As_4_S_3_ polymorphs, where the 337 cm^−1^ band of dimorphite and the 224 cm^−1^ band of paradimorphite are the pair used for the non-destructive polymorph discrimination. These reference spectra were recorded on bulk crystals, and no Raman assignment of either polymorph in film form has been published. Both bands lie between 200 and 350 cm^−1^, where the cage modes are strong and a silicon substrate has no first-order band, so both are expected to be detectable in films of micrometre thickness. The detection limit for the minority polymorph in film form has not been established and requires Raman measurements on a series of films of known polymorph ratio.

### 4.3. X-Ray Diffraction and Grazing-Incidence Geometry

Conventional θ-2θ X-ray diffraction on thin As-S films is compromised by the mismatch between film thickness, typically 200 to 2000 nm, and the millimetre-scale X-ray penetration at standard incidence, which produces substrate-dominated patterns. Grazing-incidence geometry, with a fixed incident angle of 0.5 to 3° and a scanning detector, confines the beam path within the film and raises the film to substrate signal ratio, and it is the geometry of choice for thin-film phase analysis.

Amorphous As-S films of any composition give a broad diffuse halo, and the pattern carries more information than a single maximum. The first sharp diffraction peak, near 2θ = 16° for Cu Kα, is the diffraction signature of intermediate-range order, and in binary chalcogenide glasses its position and width track the length scale over which the network retains a memory of the layer or cage periodicity [62]. In evaporated As_2_S_3_ this peak is anomalously sharp, and de Neufville et al. analysed it as the dense random packing of As_4_S_6_ molecules of about 6.77 Å diameter, the molecular glass state of the as-deposited film [63]. Illumination or annealing halves its intensity, roughly doubles its width and shifts it to a higher angle, and the pattern converges on that of the annealed film and of the bulk glass [63]. The same peak has since been used to follow intermediate-range order in As-S-based amorphous films as a function of composition [64]. Diffraction alone therefore separates the as-deposited molecular glass from the polymerised network, a distinction that Raman spectroscopy reaches through the As_4_S_4_ fraction (Section 4.2) and that underlies the irreversible photostructural change of Section 5.6.

Every vacuum route gives an amorphous film at room temperature. Thermally evaporated and spin-coated As_2_S_3_ show no sharp reflections between 10 and 90° 2θ [65], and plasma-enhanced chemical vapour deposition gives amorphous films at every composition and thickness examined [7]. Magnetron-sputtered a-As_2_S_3_ is the exception in one respect, described in Section 3.3 [38]. The one route that deposits crystalline material directly is chemical bath deposition, where as-deposited As_2_S_3_ films are nanocrystalline and index as monoclinic orpiment with Scherrer crystallite sizes of 11 to 38 nm [43], in contrast to the fully amorphous state.

Crystallisation on annealing is confirmed by sharp Bragg peaks emerging on the amorphous halo. For the As_4_S_3_ composition no annealing study of a vacuum-deposited film has been published. The low melting point of the phase, 211 °C, and the two solid-state transitions at 149 and 159 °C (Table 1) confine any useful annealing window to below 149 °C if paradimorphite formation is to be avoided, and the competition with the neighbouring As_4_S_4_ phase across that window has never been mapped. Thermally evaporated As_4_S_3_ films are in any case already microcrystalline as deposited [14], so the question for this phase is polymorph selection and not amorphous-to-crystalline conversion, and the reference patterns for indexing it are the powder data of dimorphite and paradimorphite [13].

Photoinduced diffraction changes have been reported mainly for the As_4_S_4_ phases. Light exposure expands the realgar cell anisotropically before any new phase appears, and continued exposure produces the χ phase, whose powder pattern indexes on an expanded β-As_4_S_4_ cell and not on that of realgar [24]. The same transformation has been followed at atomic resolution by in situ single-crystal X-ray photodiffraction, which established the reaction sequence, the oxygen requirement and the consequences for the thin-film geometry described in Section 4.7 [11].

### 4.4. Surface Morphology by AFM and SEM

Thermally evaporated and RF-sputtered amorphous As-S films deposited at room temperature are among the smoothest thin-film materials available. Root-mean-square (rms) roughness values of 0.3–0.8 nm are routinely reported for as-deposited a-As_2_S_3_ films, reflecting the highly mobile amorphous network during deposition. This smoothness is a key advantage for photonic waveguide applications, where surface scattering losses scale with the square of roughness. In pulsed-laser-deposited amorphous As_2_S_3_ films, atomic force microscopy resolves both the photoexpansion hillocks written below the modification threshold and the rimmed craters produced above it, and the quantitative process window is given in Section 7.4 [41].

Crystallisation substantially degrades surface smoothness. Grain nucleation and growth during thermal annealing increase AFM roughness to 3–20 nm depending on annealing temperature and time, with higher temperatures producing larger grains and coarser morphology. Photo-oxidation of As_2_S_3_ films under sub-370 nm UV irradiation in air creates a visually distinct surface morphology. SEM reveals faceted crystallite particles at the film surface confirmed by EDX as As_2_O_3_, growing preferentially at surface defect sites and grain boundaries [57]. This morphological signature is a useful combined SEM+EDX diagnostic for photo-oxidative degradation distinct from photodarkening or photocrystallisation.

### 4.5. Thermal Analysis, Glass Transition and Crystallisation Kinetics

Differential scanning calorimetry provides the glass transition temperature T_g_, crystallisation onset temperature T_x_, and the thermal stability window ΔT = T_x_ − T_g_. For stoichiometric As_2_S_3_ glass, DSC measurements by Wang et al. on laser-ablated thick films and bulk glass established that T_g_(bulk) = 211 °C (sharp endothermic peak), as-grown PLD films show a broad, ill-defined transition, annealed films reach T_g_ = 185 °C (140 °C/15 h anneal) to 200 °C (180 °C/15 h anneal), consistently 10–25 °C below bulk, reflecting residual homopolar bonding that cannot be fully eliminated by annealing [66]. The thermal relaxation activation energy was 2.12 eV for films and 2.3 eV for bulk, comparable to As-S (2.0 eV) and S-S (2.2 eV) covalent bond dissociation energies [66]. Crystalline melting endotherms begin to appear in DSC scans of films annealed above 160 °C (15 h) or at 140 °C for >200 h, indicating a very slow crystallisation process. The optimal annealing window for structural relaxation without crystallisation is below 160 °C and below 100 h. Bulk As_2_S_3_ is much more resistant to crystallisation than films [66]. The spread of T_g_ values in the broader literature (down to ~175 °C in some reports [67]) reflects genuine sensitivity to quenching rate, stoichiometry, and DSC scan rate and not measurement error. DSC analysis of As_2_S_3_ by Černošek et al. places the Kauzmann temperature at T_K_ ~ 97 °C (370 K), approximately 78 °C below the low-temperature onset of the glass transition region (~175 °C) [68]. Unlike As_2_Se_3_, where T_K_ coincides with the T_g_ onset and the glass can be considered thermodynamically ideal, As_2_S_3_ undergoes a purely kinetic glass transition. The glass retains some excess entropy of fusion and preserves undercooled-melt character. Nevertheless, T_K_ >> room temperature confirms that amorphous As-S films are thermodynamically frozen at ambient conditions and will not spontaneously relax on any practical timescale.

For As-rich compositions approaching realgar and dimorphite stoichiometry, the DSC response is more complex. Films near the As_4_S_3_ stoichiometry show sharp exothermic crystallisation events in the 120–160 °C range, well below the 220 °C T_x_ of As_2_S_3_ glass, followed by a broad softening and melting response between about 200 and 320 °C reported for As-S films of this composition range [6], which is well above the 211 °C equilibrium melting point of bulk As_4_S_3_ (Table 1) and reflects the mixed-phase state of the film. This lower crystallisation temperature reflects the cage-molecule character of the crystalline dimorphite phase. The As_4_S_3_ cage units are structurally intact in the amorphous film and require only short-range translational ordering to crystallise, without breaking and reforming the As-S bonds that control the higher crystallisation barrier in the polymeric As_2_S_3_ network. The presence of two exothermic DSC events in some As-rich films signals the sequential crystallisation of paradimorphite at lower temperature followed by its transformation to dimorphite, a polymorphic transition sequence detectable by DSC.

**Figure 8:**
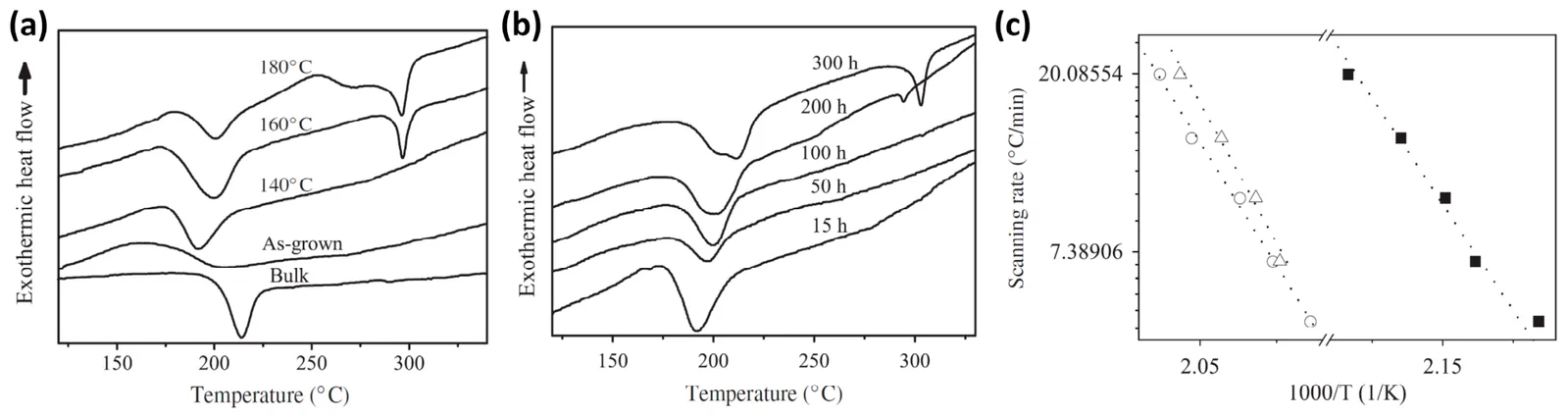
Glass transition in As_2_S_3_ films compared with the bulk glass. (**a**) Differential scanning calorimetry of the bulk glass, of as-grown films and of films annealed at several temperatures for 15 h. (**b**) The same measurement on films annealed at 140 °C for different times. (**c**) Logarithm of the heating rate against reciprocal glass transition temperature for bulk glass, for films annealed at 140 °C for 15 h and for bulk glass reheated at 240 °C for 2 h, at heating rates of 5, 7, 10, 14 and 20 K/min, the activation energy following from the slope. Reproduced from [66] with permission from John Wiley and Sons, © 2007.

The calorimetric evidence is shown in Figure 8. Figure 8a compares bulk glass, as-grown films and films annealed at a series of temperatures and separates the sharp bulk endotherm at 211 °C from the broad and displaced film transition. Figure 8b follows films annealed at 140 °C as a function of annealing time and shows the transition sharpening as structural relaxation proceeds. Figure 8c plots the logarithm of the heating rate against the reciprocal glass transition temperature over 5 to 20 K/min for bulk glass, for annealed film and for reheated bulk, from which the relaxation activation energies of 2.3 eV for the bulk and 2.12 eV for the film discussed above were obtained.

### 4.6. Medium-Range Order by EXAFS

While Raman spectroscopy characterises molecular units at the scale of 2 to 5 Å and X-ray diffraction detects periodic order at crystallite length scales above 2 to 3 nm, the intermediate range between them requires pair-correlation-sensitive probes. Extended X-ray absorption fine structure at the As K-edge, 11,867 eV, is the primary technique for the first coordination shell [69].

In amorphous As_x_S_1−x_ films the As-S coordination number decreases with arsenic content, from 3.0 at the stoichiometric composition to 2.8, 2.5 and 2.0 at x = 0.42, 0.45 and 0.50, while the As-S distance contracts from 2.28 to 2.24 Å and an As-As shell appears at 2.56 Å [70]. The AsS_3_ pyramid is therefore the short-range unit of the stoichiometric glass, and the departure from three-fold sulphur coordination is a direct measure of homopolar bonding in arsenic-rich films. The Debye–Waller factors of both shells pass through a maximum between x = 0.40 and x = 0.50, so the greatest bond mixing occurs in the same composition window [70].

Under applied pressure the As-S distance stays constant below about 15 GPa and then elongates by about 0.06 Å between 15 and 50 GPa, which is interpreted as an increase in the coordination around arsenic and the formation of a high-density amorphous state [69]. The As K absorption edge shifts by about −0.4 eV over the full range to 60 GPa, consistent with progressive metallisation, and both the structural and the electronic changes are fully reversible on decompression, the As-S distance returning to 2.275(15) Å [69]. The two changes occur in distinct pressure regimes and are independent of one another [69].

Illumination at ambient pressure does not change the coordination. Comparing annealed and light-soaked As_42_S_58_ and As_45_S_55_ films, the As-S and As-As coordination numbers and distances are identical within error, while the Debye–Waller factor of the As-As shell roughly doubles [70]. EXAFS therefore places photodarkening in increased static disorder of the existing network and not in the creation of new homopolar bonds.

The first sharp diffraction peak (FSDP) in the structure factor at about 1.1 Å^−1^ measures the medium-range layer periodicity, a memory of the layered crystalline structure, and differential anomalous X-ray scattering at the As edge shows that it is dominated by As-centred correlations extending to about 7 Å, and it therefore fills the intermediate range identified at the start of this section [71]. No EXAFS or neutron diffraction study of As_4_S_3_ thin films has been published, which is a medium-range characterisation gap for the dimorphite-composition amorphous phase.

### 4.7. Photoinduced Structural Transformations

Photoinduced structural transformations in As-S thin films are among their most technologically exploited phenomena and have been characterised by combining Raman, XRD, AFM and optical spectroscopy. The nature of the transformation depends strongly on illumination wavelength, intensity, and the film’s initial crystalline state.

In a-As_2_S_3_ under above-bandgap illumination (hν > E_g_ ≈ 2.4 eV, λ < 520 nm), the primary response is photodarkening, whose optical magnitudes are given in Section 5.6. The Raman signature of photodarkening is a strengthening of the 231 cm^−1^ (As-As) and 491 cm^−1^ (S-S) bands and a weakening of the 340 cm^−1^ AsS_3_ main peak, interpreted as photoinduced conversion of As-S bonds to homopolar bonds, a structural rearrangement that increases the disorder and redshifts the Urbach tail [50]. However, X-ray scattering, which shows the FSDP shifting to higher k with a reduced peak height at k ≈ 1.1 Å^−1^, and X-ray absorption fine structure (XAFS) data show that the As-As bond fraction increases only marginally, from 1.5% to 2.0%. The dominant structural change is therefore at the medium-range-order (MRO) level and not at the level of net bond breaking. Adjacent AsS_3_ pyramids reorient with respect to one another by rotating around the two As-S bonds of the bridging sulphur atom they share, so that the interpyramidal dihedral angle changes while every As-S bond is retained. Photodarkening is thermally reversible by annealing at ~180 °C ≈ T_g_ and partially reversible at room temperature over timescales of hours to days depending on the illumination dose [8].

In crystalline realgar (As_4_S_4_) films, the photoinduced transformation is structurally distinct and chemically irreversible. Visible-light exposure, fastest under green (532 nm), negligible under red light (633 nm) [32], drives the molecular cage rearrangement from realgar to pararealgar via an intermediate χ-phase, confirmed by the progressive evolution of the Raman spectrum from the 354 cm^−1^ dominated realgar fingerprint to the 361 cm^−1^ χ-phase signature and finally the 230/345 cm^−1^ pararealgar pattern [24,26]. In situ single-crystal X-ray photodiffraction [11] provides atomic-resolution evidence. The initiation requires oxygen (5As_4_S_4_ + 3O_2_ → 4As_4_S_5_ + 2As_2_O_3_), the intermediate As_4_S_5_ phase is detected as residual electron density in the structure, and chain reactions that need no further photoexcitation complete the transformation, yielding ~5% pararealgar in a single realgar crystal before lattice incompatibility stops the process. The thin-film geometry accelerates this process compared to the bulk crystals because diffusion path lengths for atmospheric oxygen are reduced from micrometres to nanometres, a device-stability consideration for any application employing realgar-stoichiometry films in ambient air. No lifetime measurement has been reported for a realgar-composition film under illumination in air. Encapsulation is a direct test, with a precedent in the same system, since the surface photodecomposition of ultrafast-laser-deposited As_2_S_3_ was mitigated by a PMMA overcoat (Figure 6a). Oxygen enters the transformation at the initiation step, so an encapsulant that limits oxygen access acts on that step. Suppression of the transformation under a barrier layer would show that the supply of atmospheric oxygen limits the rate in the film geometry, and an unchanged rate would show that the oxygen incorporated during deposition is sufficient to initiate it. Neither experiment has been reported.

**Figure 9:**
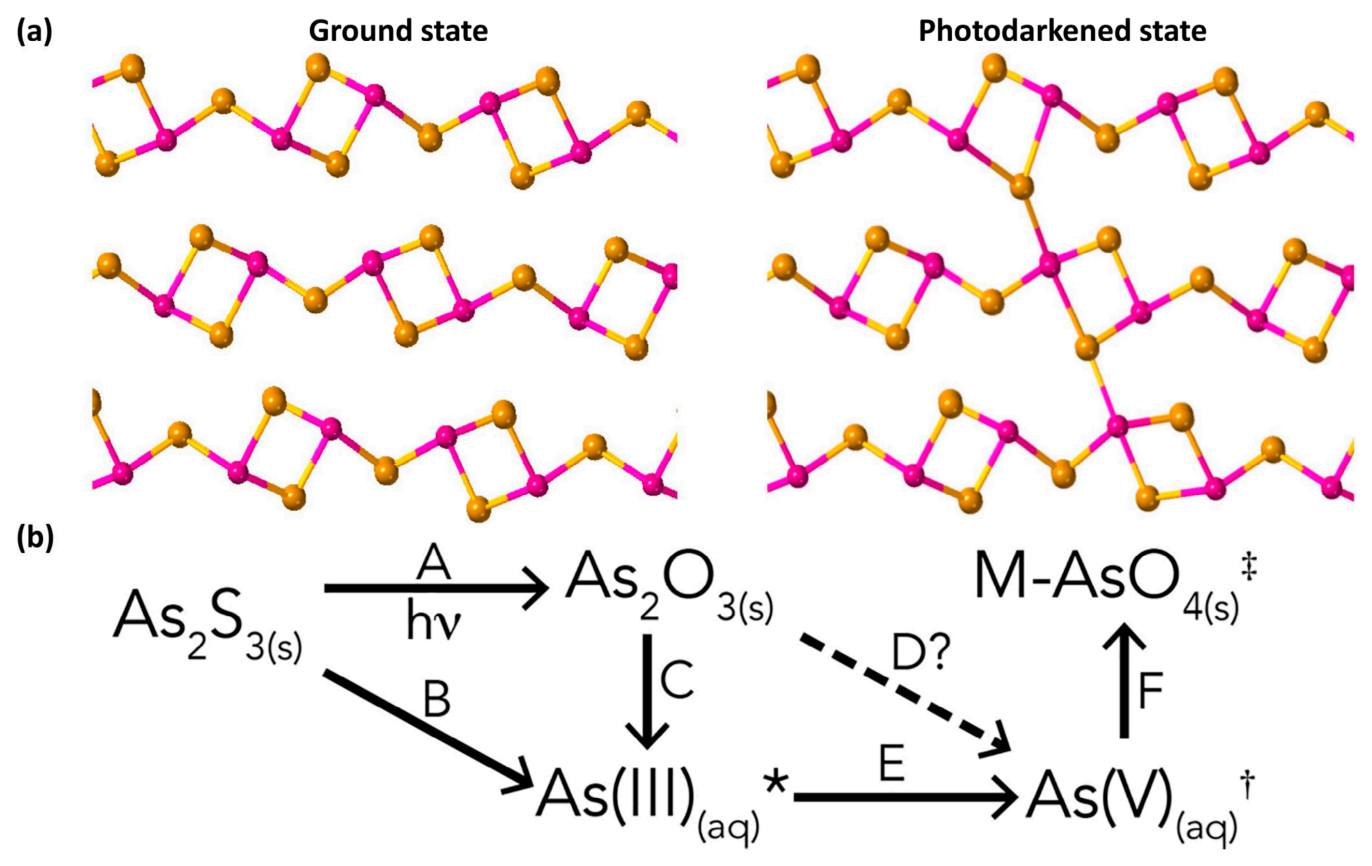
The two photoinduced routes in As-S films. (**a**) Lone-pair-mediated bond switching in As_2_S_3_ glass from density functional theory, the ground state on the left and the photodarkened state on the right, distinguished by the interlayer bond, which is the mechanism behind reversible photodarkening. Arsenic atoms are shown in violet and S atoms are yellow. (**b**) The degradation routes of orpiment, direct photo-oxidation to arsenolite As_2_O_3_ by step A and dissolution to As(III) species by step B, followed by oxidation to As(V) and precipitation as metal arsenates. Step D, drawn dashed, is the route the source argues does not occur. The asterisk marks arsenite species in solution, the dagger arsenate species in solution, and the double dagger metal arsenates such as PbHAsO_4_ or Pb_5_(AsO_4_)_3_Cl. Panel (**a**) reproduced from [72] under a Creative Commons Attribution 4.0 International Licence, © 2023 the authors. Panel (**b**) reproduced from [73] under a Creative Commons Attribution 4.0 International Licence, © 2023 the authors, published by the American Chemical Society.

Under high-intensity UV irradiation (λ ≤ 370 nm) in air, a distinct photo-oxidation pathway converts surface As_2_S_3_ to As_2_O_3_. Raman spectroscopy reveals the characteristic arsenolite peaks at 264, 366, and 555 cm^−1^ superimposed on the diminished As_2_S_3_ signal [57,58]. The surface morphology that accompanies this route is described in Section 4.5, and the same oxidation pathway has been followed on thermally evaporated films by combined surface and optical measurements [35]. A complementary surface-modification pathway under superbandgap UV (375–405 nm, ~100 mW/cm^2^) was characterised in thermally evaporated As-S films (x = 30–40 at. % As) using high-resolution X-ray photoelectron spectroscopy and low-energy ion scattering (LEIS) depth profiling [56]. UV irradiation creates a sulphur-rich surface layer. In stoichiometric As_40_S_60_ films exposed for one hour to 375 nm light in a pure argon atmosphere, the surface composition measured by XPS changes from 40 at. % As and 60 at. % S on the virgin film to 32 at. % As and 68 at. % S. The change occurs with no oxygen present, which demonstrates that oxidation is not the only mechanism. The proposed route is photovaporisation of As-rich cage molecules (As_4_S_3_ and As_4_S_4_) from the surface. Quantum-chemical calculations show that decomposition reactions producing As_4_S_3_ cages are energetically favourable (ΔE/particle < 0), and the selective decrease of the 271 cm^−1^ As_4_S_3_ Raman band under UV irradiation is consistent with UV-stimulated cage removal. LEIS profiling confirms the composition change is confined to the outermost surface layers with no bulk diffusion component. In contrast, bandgap light (525 nm) causes only photopolymerisation, partial restoration of chemical order (As-As bonds decrease from 20% to 5% at the surface and the composition is unchanged), without triggering photovaporisation or arsenolite formation. The two competing photoinduced routes, bond switching within the network and oxidation at the surface, are compared in Figure 9. Figure 9a gives the calculated ground state and photodarkened state of As_2_S_3_ glass, which differ by a single interlayer bond formed between neighbouring layers. Figure 9b places the competing surface route alongside it, in which orpiment converts either directly to arsenolite under light or by way of dissolved As(III) species, both ending in As(V) and in metal arsenates.

## 5. Optical Properties and Photoinduced Phenomena

The optical response of binary arsenic sulphide thin films is the property set on which most of the research on these materials rests. The combination of a moderate bandgap in the visible to near-infrared, a large refractive index, a broad multioctave transparency window spanning the mid-infrared, and large third-order optical non-linearity makes As-S films applicable to infrared waveguides and integrated photonic circuits to holographic data storage and all-optical signal processing. These properties, however, are not fixed, they depend sensitively on composition, phase, deposition method, and post-deposition treatment, including light exposure. The following subsections systematically review the optical bandgap, linear and dispersive refractive index, transparency and waveguide losses, thermo-optic response, non-linear optical coefficients, photodarkening optical signatures and photoinduced birefringence.

### 5.1. Optical Bandgap, Composition and Preparation Dependence

The optical bandgap of As-S thin films is not a single fixed quantity but a range determined by the interplay of composition, structural order, and preparation-induced defect concentrations, and the reported values are collected in Table 7. The absorption edge of amorphous As_2_S_3_ was mapped in detail by Kosek et al. who separated the exponential Urbach region from the quadratic Tauc region and placed the gap at 2.32 eV at room temperature and 2.43 eV at 80 K, with a temperature coefficient of −5.1 × 10^−4^ eV K^−1^ [34]. The same two regions were found in sulphur-rich amorphous As_2_S_x_ (x = 7 to 12), measured on polished bulk samples and on vacuum-deposited layers, where the edge is exponential up to α ≈ 10^3^ cm^−1^ and the optical gap increases from 2.57 eV for As_2_S_7_ to 2.59 eV for As_2_S_9_ and 2.62 eV for As_2_S_12_ [74]. Thermally evaporated films fall in the same range, 2.39 eV for an as-deposited film of 0.425 µm on glass measured by the same method [35]. The spread across the literature, roughly 2.3 to 2.4 eV, follows thermal history, measurement temperature and the absorption range over which the Tauc extrapolation is taken, and not a systematic difference between bulk and film.

Pulsed laser-deposited films grown in the pure mode-locked (ML) regime by Zakery et al. showed a further reduction to E_g_ = 2.26 ± 0.02 eV from Tauc plots measured across the 500–2400 nm spectral range using combined Swanepoel envelope and spectroscopic ellipsometry analysis [40]. The authors attributed this reduction not to a systematic structural difference between PLD and thermally evaporated films but to partial pre-exposure of the growing film surface to the ablation plume during deposition, an effect specific to the geometry of PLD that pre-induces a fraction of the photodarkening normally produced by post-deposition illumination. Films grown in the QSML regime showed a gap of 2.29 ± 0.02 eV, slightly closer to the bulk value and consistent with the lower pre-exposure fluence delivered by the 10 kHz pulse trains [40].

Quantum confinement significantly shifts the bandgap in nanostructured As-S materials. Mane et al. prepared nanocrystalline As_2_S_3_ thin films by chemical bath deposition and measured direct optical bandgaps ranging from 2.20 eV in the thickest films (520 nm, mean grain size 38 nm) to 2.58 eV in the thinnest (185 nm, mean grain size 11 nm), a shift of 0.38 eV consistent with quantum confinement once the grain size falls to the 10 nm scale [45]. These CBD films are among the very few examples where direct-gap As_2_S_3_ was confirmed (Tauc plot exponent n = 1/2), consistent with the direct character of the As_2_S_3_ band structure along certain high-symmetry directions in the Brillouin zone. Bulk melt-quenched glass is customarily treated as indirect-gap for the purposes of device modelling, though the amorphous state blurs this distinction.

As_4_S_4_ has a slightly larger gap, which is consistent with the cage-molecular electronic structure that places more of the bonding density in localised As-As and As-S cage bonds and not the delocalised network states that reduce the gap in As_2_S_3_. The wider gap of realgar correlates with its orange-red colour, against the yellow of orpiment. For As_4_S_3_, an absorption edge at approximately 2.5 eV has been reported for vacuum-sublimation deposited thin films of both the α and β polymorphs [14]. Unlike As_2_S_3_ and As_4_S_4_, dimorphite also shows a broad subbandgap absorption band at approximately 1.9 eV, and that band is polymorph-selective. It is present in slowly grown films rich in the α polymorph, paradimorphite, and absent from fast-grown films dominated by the β polymorph, which makes it an optical diagnostic of the polymorph ratio complementary to the diffraction and Raman signatures of Section 4.2 and Section 4.3 [14]. The dependence on growth velocity and substrate temperature was measured by Tsiulyanu et al. [14] and is shown with the photoresponse in Section 6.3. Its physical origin is not established, and the candidates are mid-gap states associated with stacking disorder specific to the α polymorph, transitions between bands split by the lower symmetry of the α structure, and defect states at α to β grain boundaries.

### 5.2. Refractive Index and Dispersion

The refractive index of As_2_S_3_ glass was first measured with high precision and over a broad spectral range by Rodney et al. using a commercial dark-red glass melt at three temperatures (19 °C, 25 °C, and 31 °C) across the wavelength range 0.577–11.82 µm [33]. Their measurements established the dispersion characteristics of As_2_S_3_, at 0.577 µm, n = 2.663, falling monotonically to n = 2.476 at 1.014 µm, n = 2.426 at 2.0 µm, n = 2.410 at 4.258 µm, and n = 2.383 at 9.724 µm, reaching n = 2.366 at 11.862 µm just before the onset of phonon absorption. The full dispersion was fitted to a five-term Sellmeier equation. This parameterisation describes the index to better than 0.001 across the entire transparency window and remains the most widely cited Sellmeier fit for As_2_S_3_ glass in the literature. Rodney et al. also measured a lighter-red sample (slightly lower As content) and found that its refractive index in the visible was approximately 0.09 lower than the previous sample, a significant difference driven by the lower polarisability of S-rich compositions in which excess sulphur bridges produce a less refringent network. The apparent paradox that As-rich melts appear more brilliantly red while S-rich melts appear more deeply coloured but have lower n is explained by the opposing effects of As content on electronic absorption (As-rich compositions absorb further into the visible) and on average coordination number and refractive index (As-rich compositions have higher refractive index) [33].

**Figure 10:**
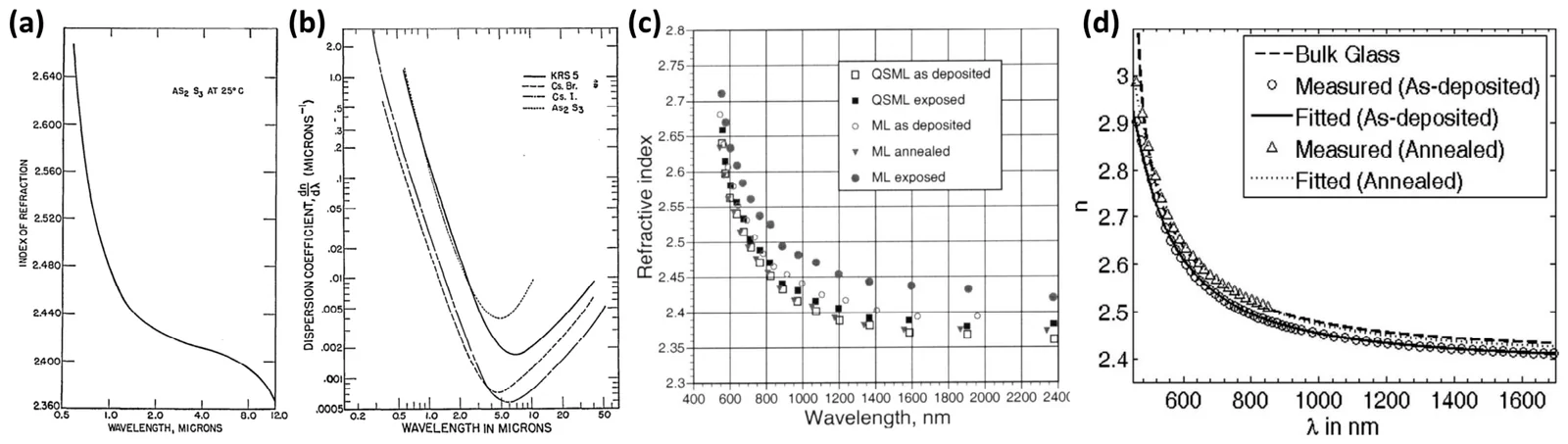
Refractive index of As_2_S_3_ in bulk and in film form. (**a**) Refractive index of the bulk glass against wavelength from 0.577 to 11.82 µm. (**b**) Dispersion of the bulk glass over the same range, with KRS-5, CsBr and CsI shown for comparison, against which As_2_S_3_ has much the higher dispersion beyond the dispersion minimum. (**c**) Refractive index of ultrafast-laser-deposited films prepared in the Q-switched mode-locked and mode-locked regimes, as deposited, annealed and after photodarkening. (**d**) Refractive index of a 377 nm magnetron-sputtered film as deposited and after annealing, measured and fitted, shown against the bulk glass curve of [33]. Panels (**a**,**b**) reprinted from [33] and panel (**c**) from [40] with permission from the Optical Society of America, © 1958 and © 2003. Panel (**d**) reproduced from [38], with the permission of AIP Publishing.

Thin-film values of the refractive index are consistently lower than bulk glass values at equivalent composition, reflecting the lower mass density, higher free volume, and larger concentration of coordination defects characteristic of as-deposited amorphous films. Zakery et al. measured n ≈ 2.46 at 800 nm by combined Swanepoel envelope analysis and spectroscopic ellipsometry for 4 µm PLD films, compared to the bulk Sellmeier value of approximately 2.49 at that wavelength [40]. Thermally evaporated films show the largest deficit of the vacuum routes, with n ≈ 2.45–2.51 at 633 nm against a bulk Sellmeier value of 2.606 at the same wavelength [2,8]. The deficit is consistent with the lower mass density and higher free volume of films condensed at room temperature without post-deposition annealing. RF-magnetron-sputtered films occupy an intermediate position. Popescu et al. obtained n = 2.852 − 0.019i at 514 nm by spectroscopic ellipsometry on stoichiometric As_2_S_3_ films deposited at optimised power and gas pressure [9], a value slightly exceeding the Rodney bulk measurement at that wavelength and suggesting that sputtered films can achieve packing densities comparable to or exceeding the bulk glass when deposition conditions favour surface adatom mobility. Solution processing gives the lowest index of any route. Prince et al. comparing spin-coated with thermally evaporated films prepared in the same laboratory, measuring n = 2.05 for the as-deposited solution film, rising to 2.3 after annealing at 150 °C for 2 h and matching the value of 2.3 obtained for their thermally evaporated film [65]. The rise tracks the loss of residual solvent and the densification that follows it. These indices come from Swanepoel envelope analysis of transmission spectra recorded between 190 and 1100 nm and are reported without a wavelength, so they establish the ordering between the two routes but cannot be placed on the dispersion curves of Figure 10. After photodarkening the refractive index increases further, which brings the PLD film values closer to the bulk glass. The magnitudes and their dependence on the deposition regime are given in Section 5.6 [40].

The dispersion data are compared in Figure 10. Figure 10a,b give the refractive index of the bulk glass and its dispersion over 0.577 to 11.82 µm from the prism measurements discussed above, with KRS-5, CsBr and CsI in Figure 10b placing the dispersion of As_2_S_3_ against the standard infrared window materials, and they remain the reference against which film values are compared. Figure 10c gives the corresponding film values for two pulsed-laser deposition regimes, as deposited, annealed and after photodarkening, and Figure 10d gives a 377 nm magnetron-sputtered film as deposited and after annealing, measured from 400 to 1700 nm against the same bulk reference. The two film routes together show that the film index lies below the bulk value across the near infrared, and that the deficit narrows as the deposition energy increases.

### 5.3. Transparency Window and Optical Waveguide Losses

Arsenic sulphide glass is one of the most studied chalcogenide infrared materials precisely because its transparency window, extending from approximately 0.6 µm (absorption edge) to approximately 11.8 µm (multiphonon cutoff at the As-S stretching phonon), covers the atmospheric transmission bands at 3–5 µm and 8–12 µm critical for thermal infrared sensing and imaging [33]. This multioctave window, representing more than four octaves of frequency, places As_2_S_3_ alongside Ge-Se and As-Se glasses as a foundation material for mid-infrared integrated photonics.

Optical waveguide losses in As_2_S_3_ thin films span more than two orders of magnitude depending on wavelength and deposition method. Near the absorption edge (633 nm), absorption in as-deposited PLD films reaches 10–20 dB/cm, rendering waveguide operation at visible wavelengths impractical except for very short interaction lengths [40]. Beyond 1000 nm the loss falls sharply. As-deposited films show less than 0.1 dB/cm across the telecommunications bands at 1300 and 1550 nm, and photodarkening raises the film loss to 0.3 dB/cm at 1000 nm and 0.2 dB/cm at 1600 nm [40]. Channel waveguides written directly into 2.5 µm mode-locked films by a scanned 514 nm or 532 nm beam, which defines the guide by photodarkening, show about 4 dB/cm at 780 nm, about 0.24 dB/cm at 1300 nm and 0.2 dB/cm at 1550 nm [40]. The telecom-band values are competitive with silica waveguides for centimetre-scale devices, and because the guide is written by photodarkening these are already the losses of the darkened material, so no further penalty follows from writing.

The state of the art for As_2_S_3_ integrated photonics is compiled in the roadmap review of Lai et al. [3]. Propagation losses as low as 0.05 dB/cm at 1550 nm have been reported for As_2_S_3_ waveguides with an effective mode area of 7 µm^2^, and 0.1 dB/cm for guides confined to 1 µm^2^ [3]. The difference is the confinement-against-loss trade-off, since the tighter mode interacts more strongly with the sidewalls. A record quality factor of Q = 1.44 × 10^7^ has been reported for an As_2_S_3_ microresonator built on an etchless trapezoidal substrate, which avoids the sidewall roughness of etched geometries [3]. Laser-induced damage thresholds measured with femtosecond pulses rise from 24.32 mJ/cm^2^ at 800 nm to 9138 mJ/cm^2^ at 1550 nm, because the higher multiphoton ionisation order required at the longer wavelength lowers the absorption. They fall again to 176.8 mJ/cm^2^ at 3 µm and 309.5 mJ/cm^2^ at 4 µm, which is attributed to O-H and S-H impurity absorption [3]. Magnetron-sputtered films fall between the two, supporting waveguide propagation losses of 0.20 ± 0.05 dB/cm at 1.5 µm [38]. By contrast, solution-processed As_2_S_3_ waveguides prepared from amine-based chalcogenide precursor solutions exhibit residual losses of approximately 9 dB/cm arising from incomplete removal of the solvent and associated carbon contamination [3], illustrating that solution deposition, while attractive for large-area coating, has not yet achieved the loss performance of vacuum-based methods for waveguide applications.

### 5.4. Thermo-Optic Effect

The temperature dependence of the refractive index of As_2_S_3_, the thermo-optic coefficient dn/dT, is strongly wavelength-dependent and falls from a large positive value in the visible to effectively zero in the mid-infrared. Rodney et al. measured dn/dT = 116 × 10^−6^ °C^−1^ at 577 nm for the dark-red bulk glass, decreasing rapidly to approximately 8 × 10^−6^ °C^−1^ at 1.7 µm and becoming effectively zero beyond approximately 4 µm [33]. This large positive thermo-optic coefficient in the visible and near-infrared arises from the temperature-induced redshift of the absorption edge, which increases the contribution of the electronic resonance to the refractive index via the Kramers–Kronig relations. At longer wavelengths, the thermal expansion of the glass reduces the mass density and hence the index, approximately cancelling the electronic contribution and driving dn/dT towards zero. This spectral behaviour makes As_2_S_3_ attractive for mid-infrared athermalised optics, where the near-zero dn/dT minimises thermal drift of the focal length, but it also implies significant thermally induced phase shifts in near-infrared integrated photonic circuits where even small temperature changes produce measurable resonance shifts.

The thermo-optic coefficient at telecom wavelengths is particularly relevant for photonic integration, where dn/dT governs both the thermal tuning efficiency of reconfigurable circuits and the thermally induced crosstalk between adjacent elements. A thermo-optic coefficient of dn/dT = 4.3 × 10^−6^ °C^−1^ at 1550 nm is tabulated for As_2_S_3_ [3], about half the bulk value of 9 × 10^−6^ °C^−1^ measured at 1.53 µm on the same glass [33]. This value is approximately forty times smaller than that of silicon (dn/dT ≈ 1.86 × 10^−4^ °C^−1^), making As_2_S_3_ substantially less sensitive to thermal fluctuations than silicon photonic platforms, an advantage for passive sensors and stable interferometric devices at the telecom band.

### 5.5. Non-Linear Optical Properties

The large third-order optical non-linearity of As_2_S_3_ is the property that has driven the bulk of photonic device research lately. The physical origin lies in the electronic structure. The As-S bond is highly polarisable, the lone-pair electrons on both As, a group 15 pnictogen, and S, a group 16 chalcogen, contribute to a high hyperpolarisability per unit cell, and the network-forming tendency of the material concentrates these high-polarisability bonds at a density comparable to or exceeding crystalline semiconductors without introducing the large two-photon absorption that limits direct-gap semiconductors at telecom wavelengths [1,75]. From the standpoint of constraint theory, As_2_S_3_ lies exactly at the mean coordination ⟨r⟩ = 2.4 that defines the rigidity percolation threshold of the chalcogenide network, the isostatic composition at which the network is neither floppy (under-constrained, too liquid-like) nor stressed-rigid (over-constrained, prone to phase separation), and at which the third-order susceptibility χ^(3)^ is maximised relative to the two-photon absorption coefficient β [3].

Bulk As_2_S_3_glass measured by Z-scan spectroscopy between 1200 and 1500 nm shows a non-linear refractive index n_2_ in the range of 100 to 200 times that of fused silica, with a maximum near 1300 nm where the ratio of the non-linear phase shift to two-photon absorption, the non-linear figure of merit T = 2βλ/n_2_, is minimised [40]. Open-aperture Z-scan traces at 1550 nm are effectively flat, indicating negligible two-photon absorption at the telecom C-band and allowing lossless non-linear phase accumulation over centimetre-scale interaction lengths. On 4 µm thick PLD As_2_S_3_ films, degenerate four-wave mixing (DFWM) measurements at 800 nm yielded |n_2_| = 2.7 × 10^−14^ cm^2^/W, approximately 90 times the silica reference value of n_2_, silica ≈ 3 × 10^−14^ cm^2^/W, confirming that the non-linearity of the thin-film material is comparable to that of bulk glass [40]. Fibre measurements on As_2_S_3_ single-mode drawn fibres consistently return n_2_ values in the range ~67–150 times silica. Asobe et al. reported n_2_ = 2.0 × 10^−14^ cm^2^/W at 1.55 μm from Kerr-shutter measurement, with χ^(3)^ = 1.0 × 10^−11^ esu, a Raman gain coefficient of 6.4 × 10^−12^ m/W and a TPA coefficient α_2_ = 6.2 × 10^−15^ m/W [1,75]. The figure of merit T = 2α_2_λ/n_2_ of 9.3 × 10^−3^ at 1.55 μm, taken from the earlier literature, indicates effectively lossless non-linear operation at the telecom C-band. The spread across the reported values follows the composition as drawn, the measurement technique, whether Z-scan, degenerate four-wave mixing or four-wave mixing spectroscopy, and whether bulk or thin-film samples were characterised.

An important caveat emerges from bulk Z-scan measurements. Cerqua-Richardson et al. performed closed- and open-aperture Z-scan at 1.55 µm on bulk As_40_S_60_ melt-quenched glass and obtained n_2_ = 4 × 10^−15^ cm^2^/W with a two-photon absorption coefficient of 0.03 cm/GW, giving a figure of merit T = 2α_2_λ/n_2_ of 2.3 [76]. Taken on its own that value would place bulk stoichiometric As_2_S_3_ above the T < 1 threshold required for net all-optical gain. It sits, however, a factor of five below the n_2_ obtained by Kerr-shutter measurement on As_2_S_3_ fibre at the same wavelength, and the corresponding T of 9.3 × 10^−3^ [75] is smaller by more than two orders of magnitude. The disagreement is carried by both inputs, the fivefold difference in n_2_ and a roughly fiftyfold difference in α_2_, and it is most plausibly a consequence of the measurement regime, since the Z-scan work used 20 ps pulses at peak intensities up to 50 GW/cm^2^ where accumulated thermal and free-carrier absorption inflate the apparent α_2_, while the fibre measurements used picosecond and femtosecond Kerr-shutter geometries. The two-photon absorption of bulk As_2_S_3_ at 1.55 µm should therefore be treated as bounded and not as determined, and the waveguide figures of merit quoted below, which are measured directly in the guiding geometry, are the more reliable basis for device comparison.

In integrated waveguide geometry, the relevant figure is not n_2_ alone but the non-linear parameter γ = n_2_ω/(cA_eff_), which also depends on mode confinement. Two conventions for the figure of merit appear in this literature. Bulk and fibre measurements report T = 2α_2_λ/n_2_, which has to fall below unity, while waveguide studies report its reciprocal FOM = n_2_/(α_2_λ), which has to exceed unity. The two are related by FOM = 2/T, so the fibre value T = 9.3 × 10^−3^ and the tabulated FOM of 208 describe the same material. The combination of the large n_2_ of As_2_S_3_ and the sub-µm^2^ mode areas achievable in their ultralow-loss waveguide platform produces a figure of merit FOM = n_2_/(βλ) exceeding 100 at 1550 nm, two orders of magnitude above the FOM > 1 threshold required for effective all-optical signal processing without two-photon loss limitations [3]. The group velocity dispersion profile of their waveguides was also suited for soliton propagation, which allows supercontinuum generation and frequency comb formation in the chalcogenide mid-infrared [3]. These results position As_2_S_3_ as the material of choice for integrated non-linear photonics in the 1–5 µm window, combining the highest non-linearity per unit loss of any established waveguide platform with CMOS-compatible lithographic processing. The comprehensive benchmark tabulation of Eggleton et al. confirms and contextualises these figures across device geometries. As_2_S_3_ fibres achieve FOM ≈ 208 with γ = 160 W^−1^km^−1^. As_2_S_3_ rib waveguides reach FOM ≈ 304 with γ = 1700 W^−1^km^−1^ and dispersion-engineered As_2_S_3_ rib waveguides with zero-dispersion wavelength near 1550 nm achieve γ = 9900 W^−1^km^−1^ with FOM ≈ 312 [1]. All As_2_S_3_ configurations produce no free-carrier effects, against silicon nanowires (FOM ≈ 0.77, significant free carriers) [1], which underscores the fundamental materials advantage of As_2_S_3_ for all-optical processing at ultrahigh bit rates. The device-level consequences of these figures, including the highest bit rate demonstrated on this platform, are treated in Section 7.1.

**Figure 11:**
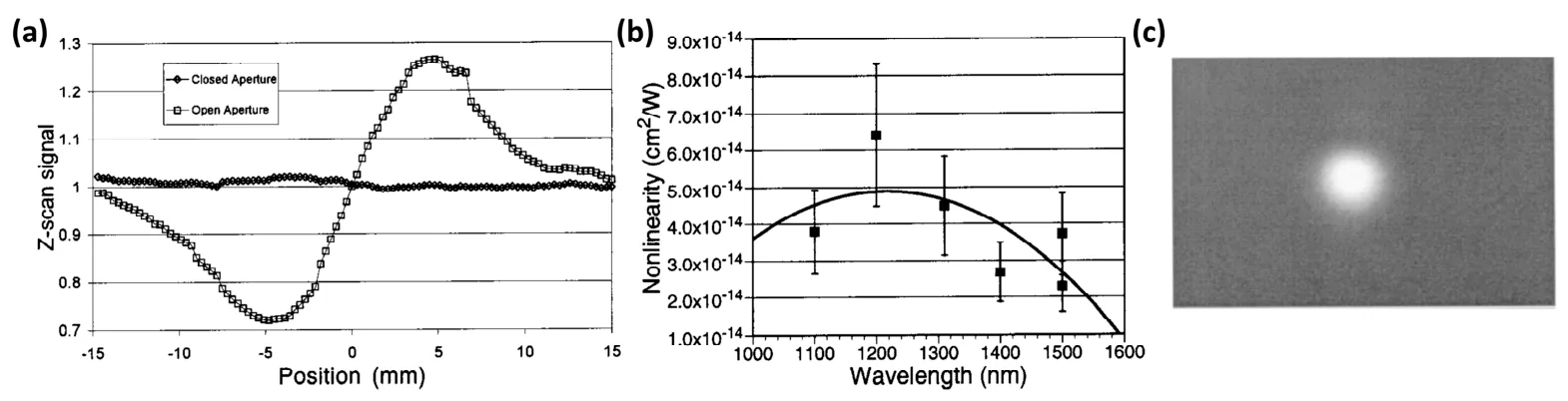
Non-linear optical response of As_2_S_3_. (**a**) Open- and closed-aperture Z-scan traces from a 2 mm bulk sample at 1550 nm, where the flat open-aperture trace indicates no measurable non-linear absorption. (**b**) Non-linearity against wavelength from Z-scan measurements over 1000 to 1600 nm. (**c**) Near-field image of the 800 nm single-mode output of a 3.5 × 2.5 µm photoinduced channel waveguide written in a laser-deposited film. Reprinted from [40] with permission from the Optical Society of America, © 2003.

The non-linear data are collected in Figure 11. The open-aperture Z-scan trace in Figure 11a shows no measurable non-linear absorption at 1550 nm in bulk As_2_S_3_, which places the two-photon coefficient at or below the detection limit of that experiment and accounts for part of the disagreement described above. Figure 11b gives the spectral dependence of the non-linearity from the same work and Figure 11c the single-mode output of a photoinduced channel waveguide of 3.5 × 2.5 µm cross-section.

### 5.6. Photodarkening, Optical Signatures, Magnitude and Kinetics

Photodarkening, the reversible or partially reversible decrease in optical transmission and increase in refractive index induced by above-bandgap illumination, is the most extensively studied photoinduced optical phenomenon in amorphous As_2_S_3_ films. The structural mechanisms underlying photodarkening were reviewed in Section 4.7. The present section focuses exclusively on the optical manifestations, namely the spectral form of the absorption edge change, the magnitude of the refractive index shift, and the dependence of both quantities on illumination fluence, intensity, and deposition history.

The foundational distinction in photoinduced optical response of a-As_2_S_3_ was established by de Neufville et al. who identified two qualitatively different photoresponse modes [63]. In as-deposited slowly evaporated films, the dominant effect is an irreversible photostructural transformation. As-deposited films consist of a dense random packing of As_4_S_6_ molecular cage units, a molecular glass retaining the cage structure of the vapour phase, and bandgap illumination or annealing near T_g_ polymerises these molecules into the covalent network glass characteristic of bulk As_2_S_3_ or sputtered films. This polymerisation produces a large irreversible refractive index increase of Δn ≈ +0.10 (n at 1.5 eV rises from 2.42 ± 0.05 as deposited to 2.51 ± 0.05 after annealing and 2.53 ± 0.05 after illumination, two treatments that leave the film at the same index within error and close to the bulk value of 2.528, although their absorption edges differ) and a corresponding absorption edge redshift [63]. The diffraction signature of this molecular glass state, and its change on polymerisation, are given in Section 4.3 [63]. Once polymerisation is complete, either by annealing or by prolonged illumination, only the second, smaller and reversible effect persists, namely photodarkening, characterised by an absorption edge shift of approximately 0.03 eV for As_2_S_3_ without measurable refractive index change (Δn < 0.01) [63]. The direct consequence for deposition method comparison is that sputtered films, which are structurally equivalent to annealed bulk glass from the moment of deposition, exhibit very weak photodarkening, the diffraction efficiency of a stored plane grating in sputtered As_2_Se_3_ is more than two orders of magnitude below that in evaporated films [63]. The same contrast should hold across the binary As-S system, so a sputtered arsenic-rich film would be expected to carry less photostructural potential than an evaporated film of the same composition.

Spectrally, photodarkening in annealed a-As_2_S_3_ manifests as a rigid parallel shift of the Urbach absorption edge towards lower photon energies, with the Urbach slope parameter Γ remaining unchanged under illumination [8]. This parallel shift, as opposed to a change in slope, indicates that photodarkening modifies the average disordered structural configuration and does not introduce a qualitatively new class of defect states with a different energy distribution. The rigid edge shift is accompanied by an increase in refractive index throughout the transparency window. Pfeiffer et al. measured Δn = 0.029 at 633 nm in annealed films after prolonged subbandgap illumination, with the index change following Kramers–Kronig consistency with the measured edge shift [8]. Pfeiffer et al. also established that the saturation of the edge shift is set by the total absorbed dose and not by the photon flux, since measurements at several fluxes fall on a common curve when the shift is plotted against the number of absorbed photons [8]. The photodarkened state is thermally reversible by annealing near the glass transition temperature (T_g_ ≈ 180 °C for As_2_S_3_). A 2 h anneal at 150 °C substantially recovers the original transmission and index [40]. In unannealed, as-deposited films a portion of the photodarkening is irreversible even after annealing, because the pre-illumination structural state of as-deposited films contains excess cage molecules and homopolar bonds that are photopolymerised into the network irreversibly [8].

Photodarkening in As-S films is strongly composition-dependent. Zakery et al. showed that, in thermally evaporated As-S films, photodarkening increases progressively with As concentration and becomes negligible at the composition As_30_S_70_ [77]. At illumination saturation (514.5 nm, Ar^+^ laser), the photoinduced refractive index change is Δn ≈ 0.11 for As_40_S_60_ (n_0_ = 2.53), decreasing to Δn ≈ 0.10 for As_33_S_67_ (n_0_ = 2.50) and zero for As_28_S_72_, consistent with a mechanism involving photoinduced formation of As-As bonds that are abundant only in As-rich compositions [77]. Tanaka et al. separated the same composition dependence into its two components across 15 to 45 at. % As and found the irreversible change to peak at As_40_S_60_ while the reversible change follows the arsenic content [78]. The peak of the irreversible component in the stoichiometric composition is consistent with the photopolymerisation of As_4_S_6_ molecules identified by de Neufville et al. [63]. These Δn values are large enough for holographic recording and grating fabrication.

The magnitude of Δn varies substantially with deposition method and illumination regime. In ML-regime PLD films, Zakery et al. measured Δn = +0.04 at 514.5 nm after 100 s of illumination at 1 W/cm^2^ (514.5 nm Ar^+^ laser), while QSML-regime films showed Δn = +0.01 under identical conditions, reflecting the lower photosensitivity of QSML films that were pre-exposed to smaller plume fluences during deposition [40]. The kinetics of photodarkening as a function of fluence and intensity define three regimes. Below a threshold fluence, no measurable Δn is produced, at intermediate fluence, photodarkening is fully reversible by annealing while at high fluence, a permanent component of Δn persists after annealing, indicating structural changes that cannot be thermally reversed within the glass transition kinetics [40]. PLD films are 3–10 times less photosensitive than thermally evaporated films of nominally identical composition, consistent with the partial pre-exposure effect during PLD deposition discussed in Section 5.1. In holographic recording geometries using thermally evaporated films, far larger refractive index modulations have been reported, with values up to Δn ≈ 0.12 reported for chalcogenide films in the saturated high-fluence regime [10]. The grating performance that follows from it is described in Section 7.2. Reaching the saturation Δn required exposure fluences of several kJ/cm^2^, orders of magnitude beyond the weak-illumination regime explored in waveguide writing experiments, highlighting that photodarkening is not a linear process and that the photosensitivity spectrum cannot be characterised by a single number. Pulsed illumination at high peak intensities can achieve equivalent or larger Δn at substantially lower time-averaged fluences, as discussed in the comprehensive review of pulsed light effects by Tanaka et al. [2], which cites optimised pulsed-illumination Δn up to 0.12 in highly controlled configurations.

The connection between the optical Δn and the structural changes described in Section 4.7 deserves explicit comment. As shown in Section 4.7, the As-As homopolar bond fraction increases by only about half a percentage point on photodarkening, a very small structural change [8]. Yet this small bond-fraction change produces a measurable Δn because the dielectric function of As_2_S_3_ in the visible and near-UV is strongly sensitive to MRO and the photoinduced densification. The photoinduced densification of the FSDP in the structure factor, reflecting compaction of the MRO periodicity (the layer-to-layer correlation at approximately 5 Å), shifts the electronic oscillator strength towards lower energies through a redistribution of the density of states at the valence band edge [8]. The MRO change, amplified through the dielectric response, produces the optically observable photodarkening and not the atomic-bond change.

Direct Raman evidence for As-As bond formation as the operative photodarkening mechanism was synthesised in the broader photoinduced effects framework [79]. Raman spectra measured with 647.1 nm excitation from annealed evaporated a-As_2_S_3_ films, before and after exposure to 488 nm Ar-ion laser light (2.54 eV; E_g_ ≈ 2.4 eV), showed a reproducible increase in the relative intensity of the band at 231 cm^−1^, which earlier studies had assigned to As-As stretching modes in the chalcogenide network [79]. From the magnitude of this intensity increase, Owen et al. estimated that about 6 percent of As-S bonds are converted at saturation [79]. The XAFS estimate of Pfeiffer et al. is an order of magnitude smaller, an increase in the As-As bond fraction of about 0.5 percentage points [8]. The two techniques weight the network differently, since Raman intensity responds to the polarisability of the newly formed bonds while XAFS counts coordination, and the discrepancy between the two estimates remains unresolved. A third determination, by X-ray photoelectron spectroscopy of the film surface, shows that the sign of the change depends on composition. In As_40_S_60_ and As_50_S_50_ films, near-bandgap illumination reduces both the As-As and the S-S homopolar components, whereas in As_45_S_55_ the As-As_3_ component doubles from 5.1 to 10.1 percent and the As-SAs_2_ component rises from 6.6 to 27.6 percent, an effect the authors attribute to As_4_S_3_ structural units [37]. Cycling between the annealed and photodarkened states was fully reproducible over multiple cycles, confirming that the bond conversion is both optically driven and thermally reversible at T_g_, with no irreversible bond-fraction drift detectable in the Raman signatures [79]. Owen et al. also situate photodarkening within a taxonomy of photoinduced effects in amorphous chalcogenides, including reversible effects (local atomic structure changes, photodarkening/photobleaching, photodecomposition) and irreversible effects (photocrystallisation, photopolymerisation, morphological changes, photodissolution of metals, photovaporisation) [79]. This taxonomy clarifies that the large irreversible polymerisation of as-deposited As_4_S_6_ molecular cages belongs to the photopolymerisation category, while the reversible photodarkening of annealed films belongs to the category of local atomic structure changes. In the same work, compositional analysis of photodissolution of silver into As-S films showed that only the As_30_S_70_ composition yields a homogeneous photodoped product (the tie-line to Ag passes through the glass-forming region in the Ag-As-S phase diagram), while As_2_S_3_, As_38_S_62_, and As_20_S_80_ all produce phase-separated structures, a composition sensitivity that is governed by the phase diagram and not by the photodarkening mechanism, since As_30_S_70_ is the composition at which photodarkening itself becomes negligible [79].

**Figure 12:**
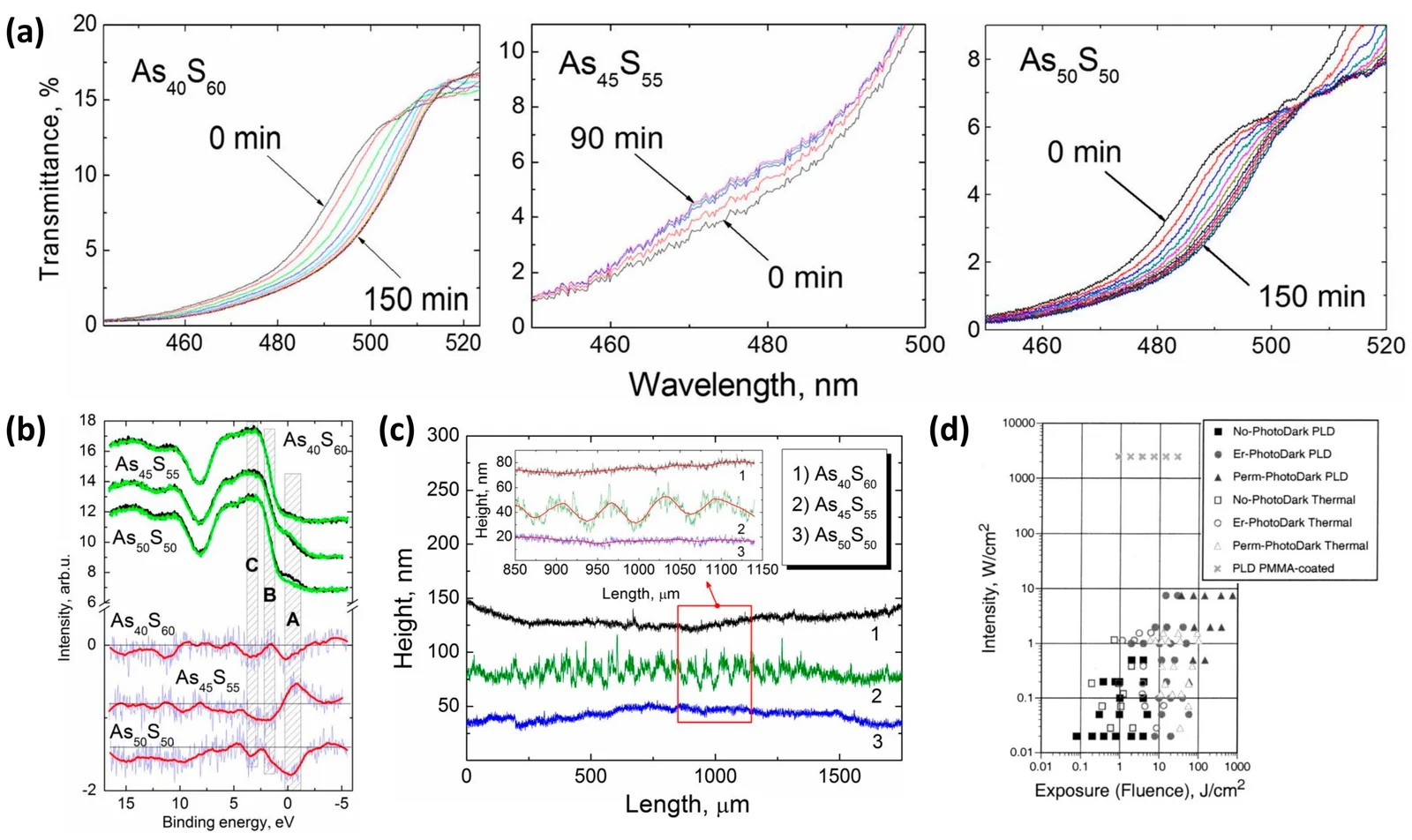
Photodarkening in amorphous As_x_S_100−x_ films and the changes that accompany it. (**a**) Shift of the optical absorption edge of As_40_S_60_, As_45_S_55_ and As_50_S_50_ films under 532 nm illumination. (**b**) Valence band spectra of the top nanolayers of the same films before and after illumination, with the difference spectra. (**c**) Surface relief written by illuminating through a copper grid of 60 µm period, showing the accompanying mass transport. (**d**) Photodarkening regimes in laser-deposited and thermally evaporated films, where the symbols distinguish no photodarkening, erasable photodarkening and permanent photodarkening. Panels (**a**–**c**) reproduced from [37] under a Creative Commons Attribution 4.0 International Licence, © 2017 the authors. Panel (**d**) reprinted from [40] with permission from the Optical Society of America, © 2003.

The optical and structural signatures of photodarkening are collected in Figure 12. Figure 12a gives the shift of the absorption edge of As_40_S_60_, As_45_S_55_ and As_50_S_50_ films under 532 nm illumination, which places the edge shift described above across the composition range. Figure 12b gives the valence band spectra of the top nanolayers of the same films before and after illumination, together with the difference spectra, which locate the electronic change at the illuminated surface. Figure 12c gives the surface relief written by illuminating through a copper grid of 60 µm period, which is the mass transport that accompanies the optical change. Figure 12d separates the fluence and intensity conditions that produce no photodarkening, erasable photodarkening and permanent photodarkening, for laser-deposited and for thermally evaporated films.

### 5.7. Photoinduced Anisotropy

When amorphous As_2_S_3_ is illuminated with linearly polarised above-bandgap light, a photoinduced optical birefringence develops in the plane perpendicular to the light propagation direction, a phenomenon termed photoinduced anisotropy (PIA). The anisotropy saturates under prolonged exposure at constant intensity and is removed by subsequent illumination with unpolarised light of the same photon energy or by thermal annealing below T_g_ [2,8]. PIA is distinctly different from photodarkening. Photodarkening produces an isotropic index increase (Δn is the same for all polarisation directions of probe light), while PIA produces a polarisation-dependent index shift (different Δn values for probe polarisations parallel and perpendicular to the pump polarisation). Both phenomena can coexist and partially superpose in exposed films, complicating their separation unless probe polarimetry is performed at multiple angles.

The mechanism of PIA is not settled. Fritzsche attributed the anisotropy to the anisotropic optical excitation of structural units whose transition dipoles are aligned with the field, with the resulting bond rearrangement depleting the population aligned along the pump polarisation [80]. Tikhomirov and Elliott developed a model based on light-stimulated reorientation of charged defect pairs, which also accounts for the dark relaxation of the induced anisotropy [51,81]. Tanaka et al. found a change in the X-ray structure factor near 1 Å^−1^ under polarised illumination and a photoinduced birefringence that peaks at a temperature dependent on the photon energy and concluded that the anisotropy arises from the orientation of quasicrystalline clusters [59]. Kolobov et al., using reflectance-difference spectroscopy, found that the anisotropy is induced at photon energies well above that of the inducing light and that its sign reverses between the low-energy and high-energy parts of the spectrum, which they interpreted as a redistribution between bonding and lone-pair electrons [82]. What the models share is a reorientation of anisotropic structural units under polarised excitation, and they differ in which unit is held responsible.

The magnitude of PIA and its ratio to the isotropic photodarkening component are both strongly dependent on the degree of structural freedom in the glass network. As_2_S_3_ sits at the isostatic composition ⟨r⟩ = 2.4 described in Section 5.5, where the network is exactly constrained and can accommodate bond reorientation without the stress build-up of an over-constrained network. PIA offers a grating mechanism independent of photodarkening, which is the basis of proposals for polarisation holography. No published data exist on photoinduced anisotropy in As_4_S_4_ or As_4_S_3_ thin films. Each of the models above requires structural units that can reorient under polarised excitation within a disordered network, whether these are transition dipoles, charged defect pairs or quasicrystalline clusters. In a crystalline film the As_4_S_4_ and As_4_S_3_ cages occupy lattice sites and cannot reorient in this way, so PIA is expected to be absent in crystalline films. In amorphous films of the same compositions the absence of data is a measurement gap, and in mixed amorphous-crystalline films birefringence averaging may further reduce the measured anisotropy. The two cases can be compared directly on crystalline As_4_S_3_ films and on the amorphous dimorphite-stoichiometry films obtained by PECVD (Section 3.5).

## 6. Electrical and Optoelectronic Properties

The electrical properties of binary arsenic sulphide materials span an unusually wide range, over more than twelve orders of magnitude, from single-crystal realgar at one extreme to nanocrystalline chemical-bath-deposited As_2_S_3_ at the other, driven primarily by phase, morphology and carrier type and not by composition alone. The transport mechanisms are equally diverse. Band conduction limited by lattice scattering in one carrier type within the same crystal can coexist with thermally activated intermolecular hopping in the complementary carrier, while in dimorphite thin films the dominant conduction channel is space-charge-limited current (SCLC) governed by an exponential trap-energy distribution. This section reviews these transport mechanisms systematically and examines the optoelectronic consequences, photoresponse spectra, bipolar photoconductivity, chemical modulation of SCLC by adsorbed analyte gases, and laser-induced photoluminescence, that make As-S thin films candidates for UV sensors, chemical sensing elements, and luminescent micro/nanodevice components.

### 6.1. Dark Conductivity and Resistivity

The dark conductivity of As-S thin films is among the most phase-sensitive properties of this material family, and understanding it requires distinguishing clearly between amorphous As_2_S_3_, nanocrystalline As_2_S_3_, and the crystalline As_4_S_4_ and As_4_S_3_. The resistivity, carrier type and activation energy of each phase are collected in Table 8.

Amorphous As_2_S_3_ prepared by thermal evaporation under standard vacuum conditions is a prototypical chalcogenide insulator. Its room-temperature resistivity is typically reported in the range of 10^9^–10^12^ Ω·cm, placing it among the more resistive materials accessible in thin-film form [83]. Conduction in amorphous As_2_S_3_ is p-type, as is typical for chalcogenide glasses where the valence-band tail states, derived primarily from the lone-pair sulphur orbitals, are more accessible to thermally activated hole carriers than the conduction-band tail states are to electrons [84]. The assignment is based on the sign of the thermoelectric power and not on the Hall effect, because the chalcogenide glasses display the sign anomaly in which the Hall coefficient carries the opposite sign to the thermopower, so that a Hall measurement on these materials does not identify the majority carrier [84,85]. The thermally activated conductivity of a-As_2_S_3_ films is governed by hopping and band-tail conduction, established for this material by combined a.c. and d.c. conductivity measurements on evaporated films [86]. Reported d.c. activation energies for amorphous As_2_S_3_ scatter over a wide range depending on preparation and on the temperature interval fitted, with values near 0.20 eV for evaporated films, well below the half-gap value that a Fermi level pinned at mid-gap would require in a material with E_g_ ≈ 2.39 eV for evaporated films (Section 5.1).

A deeper insight into the electronic structure of amorphous As_2_S_3_ was provided by Van den Keybus et al. through non-resonant interband Faraday rotation (FR) measurements at 480–850 nm and fields up to 4 T, over the temperature range 4–300 K [83]. Faraday rotation in the non-resonant regime probes the effective optical gap experienced by the oscillator sum that governs magneto-optical response, a quantity distinct from, and typically larger than, the absorption edge seen in transmission. Analysis of the FR spectra using three classical dispersion models converged on E_g_^FR^ ≈ 3.1 eV (range 2.97–3.31 eV across models, with asymmetry parameter z ≈ 0.2) [83], lying approximately 0.74 eV above the optical absorption edge of 2.36 eV. This elevated energy is fully consistent with the Wemple single-oscillator dispersion fit for a-As_2_S_3_ (E_g_^direct^ ≈ 3.1 eV), indicating that Faraday rotation reports on a direct-like transition at an effective high-symmetry point of the amorphous electronic structure and not the indirect optical threshold. No free-carrier Faraday rotation contribution was resolved in intrinsic a-As_2_S_3_, confirming that the signal is entirely interband in origin and corroborating the characteristically high dark resistivity of evaporated films. The FR magnitude was essentially temperature-independent below 50 K and decreased by approximately 0.1 eV between 50 K and room temperature, a modest shift that parallels the thermal broadening of the optical gap in chalcogenide glasses [83].

The nanocrystalline As_2_S_3_ thin films deposited by CBD by Mane et al. exhibit substantially lower resistivities and n-type conduction and not p-type, as confirmed by thermoelectric power (Seebeck) measurements in which the hot probe was always the positive terminal [45]. In that study, films grown from an aqueous bath containing arsenic trioxide, sodium EDTA, and thioacetamide at pH 2.5 and 6 °C substrate temperature had grain sizes (Scherrer broadening from the (311) XRD reflection) ranging from 11 nm in the thinnest films (185 nm) to 38 nm in the thickest (520 nm). The room-temperature dark resistivity decreased from approximately 5.1 × 10^5^ Ω·cm for the thinnest films to approximately 3.5 × 10^4^ Ω·cm for the thickest, a factor of ~15 reduction attributed to a corresponding decrease in grain-boundary scattering and surface-state density as the crystallite dimensions grow. The activation energies extracted from Arrhenius plots of log ρ vs. 1/T decreased in parallel, from E_a_ = 0.15 eV for 185 nm films to E_a_ = 0.07 eV for 520 nm films, indicating that the dominant conduction pathway in all CBD samples is shallow-trap thermally activated transport through grain-boundary potential barriers and not bulk bandgap controlled intrinsic conduction [45]. The log ρ against 1/T curves show two distinct linear regimes at every film thickness, which both original reports attribute to an α to β phase transition in As_2_S_3_ [43,45], an assignment that the transport data alone do not establish. The n-type polarity of CBD films stands in contrast to the p-type conductivity of thermally evaporated amorphous As_2_S_3_ and likely reflects the incorporation of electron-donor species from the thioacetamide decomposition pathway during chemical bath synthesis. Thioacetamide hydrolysis can introduce sulphur vacancies acting as shallow donors in the As_2_S_3_ lattice, a mechanism that would simultaneously account for the anomalously low E_a_ values relative to the bandgap [45].

Prince et al. provided a quantitative comparison of electrical and optical properties of thermally evaporated versus solution spin-coated amorphous As_2_S_3_ films using indium (ohmic) electrodes [65]. The thermally deposited films (1–2 µm, deposited at 10 nm/s, ~10^−6^ Torr) showed room-temperature DC conductivity σ_dc_ = 7.53 × 10^−9^ Ω^−1^cm^−1^ (ρ ≈ 1.3 × 10^8^ Ω·cm) with activation energy ΔE = 0.20 eV and pre-exponential factor σ_0_ = 1.63 × 10^−5^ Ω^−1^cm^−1^ [65]. The σ_0_ value, well below the threshold of 10^4^ Ω^−1^cm^−1^ for extended-state transport established by Mott, confirms that conduction occurs in localised band-tail states near the Fermi level and not by mobile carriers above the mobility edge. Solution spin-coated films prepared from ethanolamine solutions initially showed lower conductivity (4.6 × 10^−9^ Ω^−1^cm^−1^) due to residual solvent, but on annealing at 150 °C their conductivity increased to 1.30 × 10^−8^ Ω^−1^cm^−1^ with a reduced activation energy of ΔE = 0.12 eV, slightly exceeding the thermally deposited value, attributed to partial defect annealing and bond rearrangement [65]. The structural origin of this difference is visible in the Raman spectra. Solution-deposited films carry cage and homopolar features that are absent from thermally deposited films of the same composition, and annealing removes them progressively as the network relaxes towards the ordinary glass structure [65]. The band positions and their competing assignments are given in Section 4.2 and Table 6. The optical gaps and refractive indices of the same two film sets, before and after annealing, are given in Section 5.1 and Section 5.2 [65].

At the other extreme of resistivity, single-crystal realgar (As_4_S_4_) prepared by vapour phase growth at 10^−6^ Torr reaches dark resistivities exceeding 10^16^ Ω·cm, approaching the intrinsic insulator limit, when kept in the dark for several days prior to measurement to allow complete relaxation of any photogenerated or injection-induced excess carriers [30]. This resistivity is consistent with the molecular crystal character of realgar. The As_4_S_4_ cage molecules are held together by van der Waals forces in the crystal lattice, which severely suppresses intermolecular orbital overlap and enforces near-complete carrier localisation in the dark ground state. The dielectric constant of this single crystal was measured to be ε = 6.5, comparable to other molecular chalcogenide crystals and appreciably lower than the values near 8–10 characteristic of more covalent or ionic wide-gap semiconductors [30].

### 6.2. Charge Transport Mechanisms in Crystalline Phases

The most detailed mechanistic picture of charge transport in a crystalline As-S phase comes from the classic study of Street et al. on single-crystal realgar, which resolved two distinct transport mechanisms in one crystal. Within the same crystal, holes and electrons travel by fundamentally different physical processes [30]. This bimodal transport, rarely observed with such clarity in a single material, arises directly from the molecular crystal structure of As_4_S_4_ and has important implications for understanding charge mobility limitations in any As-S crystalline system where intermolecular distances are large.

Street et al. determined carrier mobilities using transient space-charge-limited current measurements, in which the transit time of an injected carrier pulse is extracted from the shape of the current transient under conditions of trap-free space-charge-limited flow. For hole transport, the room-temperature mobility was µ_h_ = 12 cm^2^/Vs, a value consistent with band conduction in a wide-gap semiconductor, and the temperature dependence followed a T^−5/2^ power law [30]. Street et al. attribute the hole transport to band conduction limited by lattice scattering [30]. The exponent is steeper than the T^−3/2^ expected from acoustic deformation-potential scattering alone, which points to a polar optical phonon contribution, as in the III-V compound semiconductors. The physical interpretation is that holes occupy delocalised valence-band states formed by the overlap of sulphur lone-pair orbitals across the cage-molecule network, and the primary resistance to their motion is the destruction of phase coherence by thermally excited lattice vibrations. In a molecular crystal, this is a non-trivial finding. The As_4_S_4_ cage connectivity provides sufficient intercage orbital overlap in the valence-band manifold to support band conduction for holes, despite the large intermolecular van der Waals gap.

The situation for electrons differs. The room-temperature electron mobility was µ_e_ = 2 × 10^−2^ cm^2^/Vs, almost three orders of magnitude smaller than µ_h_, and the transport mechanism was identified as intermolecular hopping [30]. Above approximately 40 °C, the electron mobility follows an activated Arrhenius behaviour with a polaron-like hopping activation energy Ep = 0.14 eV, corresponding to the energy required to distort the local molecular cage environment and enable electron transfer to a neighbouring site. The extracted hopping distance was α = 5.17 Å and the hopping attempt frequency P = 1.4 × 10^11^ s^−1^ at 300 K, values consistent with nearest-neighbour As–As intercage distances in the realgar crystal structure, confirming that electrons hop between adjacent As atoms of neighbouring As_4_S_4_ cages via the van der Waals contact. The conduction-band states of realgar, derived primarily from the empty As 4p anti-bonding orbitals of the cage, are far more localised than the valence states because the anti-bonding character reduces orbital overlap across the intercage gap. The result is that electrons find themselves in narrow, strongly localised conduction-band states where polaron formation is energetically favourable and band transport is suppressed.

Below 40 °C, the electron mobility departs from simple activated hopping and exhibits a higher apparent activation energy, E_a_ = 0.4 eV [30]. Street et al. analysed this low-temperature regime using the trap-modulated transport theory, where the effective drift mobility is reduced from the band (or hopping) mobility by the fraction of time carriers spend immobilised in deep traps. They extracted a trap depth E_T_ = 0.26 eV below the conduction-band edge and a trap density N_T_ = 3 × 10^16^ cm^−3^, a density high enough for the traps to control the drift mobility below 40 °C while leaving the material undoped in the conventional sense. The trap depth follows directly from the two activation energies, since the low-temperature behaviour is governed by E_a_ = E_p_ + E_T_, so that E_T_ = 0.40 − 0.14 = 0.26 eV [30]. This detailed mechanistic decomposition, band holes, hopping electrons, shallow traps, and the crossover between hopping-dominated and trap-dominated regimes, shows how the molecular crystal architecture of realgar simultaneously enables two very different transport pathways in the same sample.

**Figure 13:**
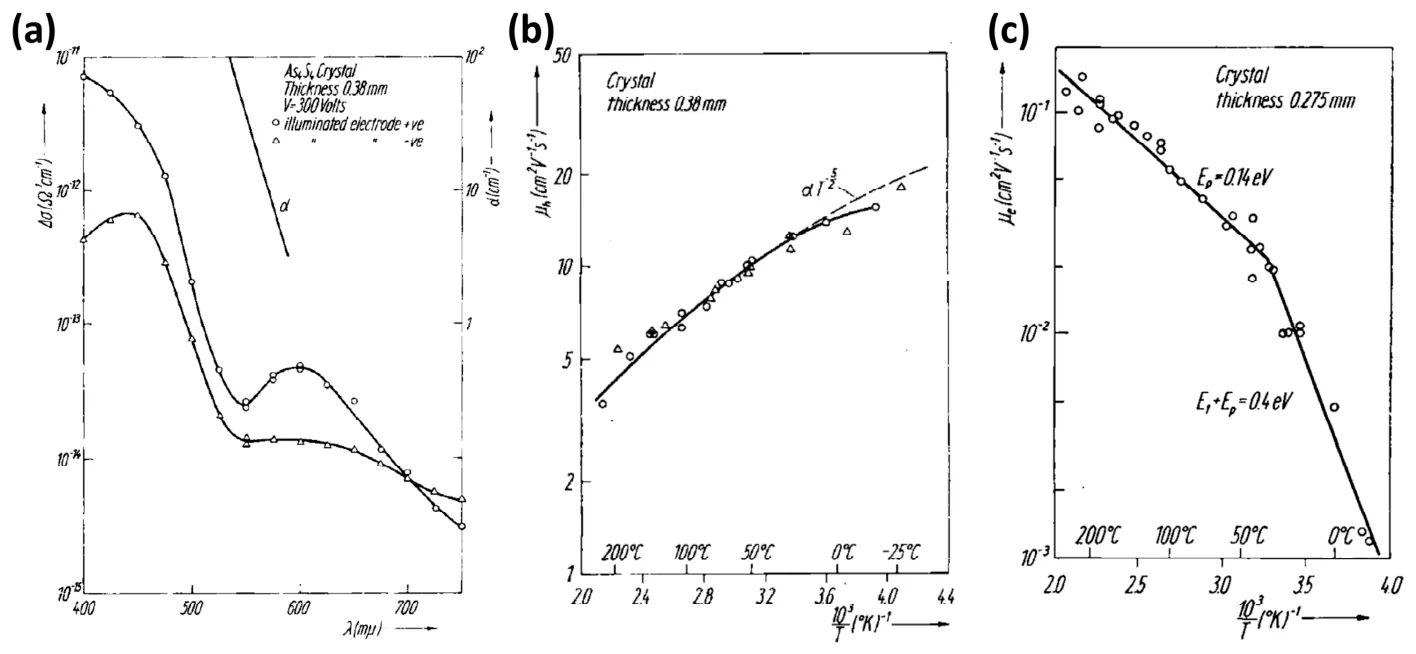
Carrier transport in single-crystal realgar. (**a**) Photoresponse curve between 400 and 700 nm with the optical absorption coefficient on the right-hand axis, recorded with the illuminated electrode biased positive and negative. (**b**) Temperature dependence of the hole mobility, following a T^−5/2^ power law. (**c**) Temperature dependence of the electron mobility, falling on two straight-line segments with the change of slope at 40 °C, the activation energy being 0.14 eV above and 0.4 eV below. Reproduced from [30] with permission from John Wiley and Sons, © 1966.

The transport data are shown in Figure 13. Figure 13a gives the photoresponse of the crystal with the optical absorption coefficient on the same axis, which locates the photoresponse with respect to the absorption edge and is discussed in Section 6.3. Figure 13b,c give the temperature dependence of the two mobilities over the same interval and place the T^−5/2^ hole behaviour and the activated electron behaviour showing the near-thousandfold separation between the two carriers in one crystal.

The situation in dimorphite differs markedly. No direct mobility measurement has been reported for dimorphite thin films, and the current–voltage characteristics measured by Marian et al. show that the film response is not governed by band transport at all but by space-charge-limited conduction [15]. SCLC arises when the density of carriers injected from a contact exceeds the equilibrium free carrier concentration in the bulk, so that the injected charge accumulates near the injection electrode and reshapes the field distribution across the device. Where traps are present, the progressive filling of those traps as the voltage rises makes the current–voltage relationship a direct probe of the trap energy distribution, and Marian et al. found As_4_S_3_ films in a metal–semiconductor–metal structure with aluminium electrodes to be in exactly this regime [15].

The devices studied by Marian et al. consisted of Al-As_4_S_3_-Al sandwich structures on glass substrates, with film thicknesses of 7–8 µm deposited by vacuum thermal evaporation. A central finding was that film morphology, controlled by deposition rate, has a decisive influence on transport behaviour. Films deposited at 0.2 µm/h grew in a fibrillar, porous morphology dominated by the α polymorph of dimorphite (para dimorphite), while films deposited at 5.0 µm/h produced smooth, compact layers dominated by the β polymorph (dimorphite). The fibrillar films were so resistive that measurement noise precluded reliable characterisation, and only the smooth β-dominant films were usable for transport studies. The same morphology dependence has been reported in other vapour-deposited chalcogenide systems where fast deposition promotes kinetically trapped dense phases and slow deposition allows the formation of open fibrillar networks with a larger surface area and higher defect density [15].

In the smooth β-dominant films measured in air, the current–voltage relationship followed a power law I ∝ U^m^ with m = 3.2, clearly above the m = 2 value expected for the classical trap-free Mott–Gurney SCLC regime and the m = 1 value expected for ohmic conduction [15]. The exponent m > 2 is the fingerprint of SCLC in a material where traps are distributed exponentially in energy below the band edge, following N(E) = N_0_ exp[(E_v_ − E)/Δ], where Δ is the characteristic energy width of the trap distribution and E_v_ is the valence-band edge. Marian et al. extracted Δ = 0.055 eV from the air-ambient measurement. The thickness exponent, the power-law dependence of current on electrode separation at constant voltage, was approximately 5, which is consistent with SCLC in an exponential trap distribution (where theory predicts an exponent of 2m − 1 = 5.4 for m = 3.2) and provides independent confirmation of the SCLC interpretation and not bulk-limited ohmic or Schottky-barrier transport [15]. The physical picture is therefore of majority carriers injected from the aluminium contacts into a trap-rich dimorphite layer, with the injected charge progressively occupying trap states as the bias is increased. The steep U^3.2^ dependence reflects the rapid emptying of shallow traps and the approach towards the trap-filled-limit transition at higher voltage.

**Figure 14:**
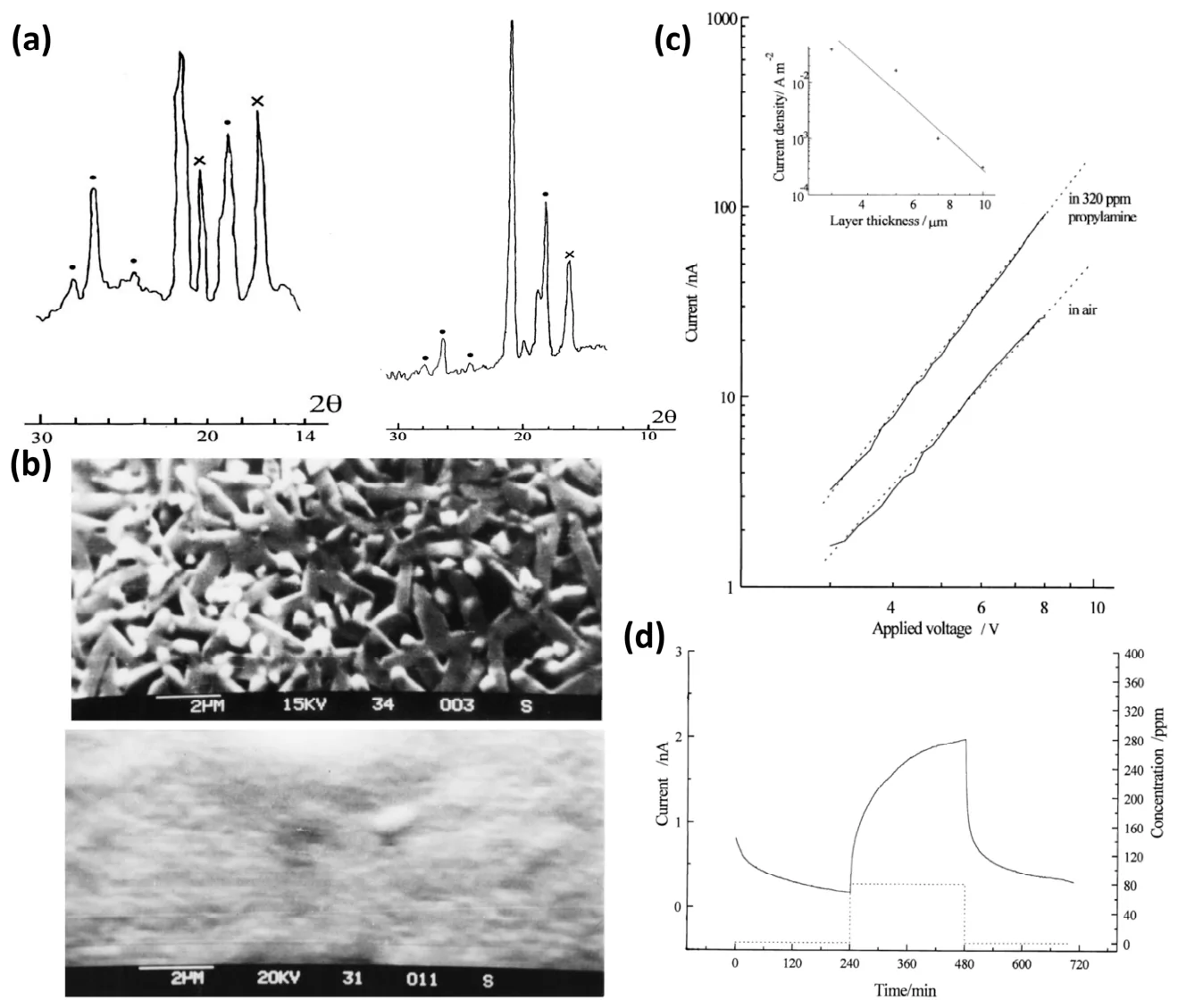
Growth velocity sets the polymorph ratio in thermally evaporated As_4_S_3_ films and with it the transport. (**a**) X-ray diffraction patterns of films grown at 0.2 µm/h and at 5.0 µm/h, with the α (•) and β (×) dimorphite reflections marked. Both polymorphs are present at both velocities, and the β proportion increases with growth velocity. (**b**) Scanning electron micrographs of films grown at the same two velocities, fibrillar and porous at 0.2 µm/h and smooth and compact at 5.0 µm/h. (**c**) Current–voltage characteristic above 3 V in ambient air and in 320 ppm propylamine vapour, plotted logarithmically, showing space-charge-limited conduction through an exponential trap distribution. The inset shows current density against layer thickness. (**d**) Transient of the gas-induced current on exposure to 80 ppm propylamine, following the concentration profile shown beneath it. Reproduced from [15], with permission from Elsevier, © 2000 Elsevier.

The predilection of metal electrodes to form blocking Schottky–Mott barriers on chalcogenide glasses, and hence to impose bipolar saturation in symmetric M-ChG-M structures, was characterised in detail by Tsiulyanu et al. for As_2_Se_3_ [87], whose study used asymmetric Te-As_2_Se_3_-Al structures in which the tellurium contact behaves ohmically (established by internal photoemission) and the aluminium contact forms a Schottky–Mott barrier. The I-U analysis revealed two distinct transport regimes. At high temperatures (T > 60 °C), thermionic emission over the barrier dominated with barrier height φ_b_ ≈ 1.1 eV (consistent with internal photoemission data). At low temperatures, a competing tunnel mechanism to vacant trap states in the chalcogenide glass is observed, with activation energy φ_t_ ≈ 0.2 eV and ideality factor n ≈ 5 [87]. The room-temperature resistivity of the As_2_Se_3_ neutral region extracted from the ohmic series resistance was ρ = 1.6 × 10^12^ Ω·cm. The paper notes that most metallic electrodes, including Al, Mg, Sb, Cr, and Ni, form blocking barriers on both As_2_S_3_ and As_2_Se_3_ chalcogenide glasses, such that symmetric Al-As_2_S_3_-Al and Al-As_4_S_3_-Al structures exhibit bipolar saturation as their primary I-U signature [87]. Marian et al. cite the same analysis but report the opposite behaviour for their own films, whose current–voltage characteristics were asymmetric and showed no tendency to saturate, which they read as the absence of a depletion region at both contacts and as evidence that the current is set by injection from the aluminium electrodes together with transport through the dimorphite film [15]. They assign the asymmetry to the two contacts being formed differently, As_4_S_3_ deposited onto Al for the lower one and Al deposited onto As_4_S_3_ for the upper. Bipolar saturation was in fact reported for Al-As_4_S_3_-Al structures by Tsiulyanu et al., whose upper electrode was semitransparent and not a full aluminium layer [14], so the two studies of the same structure disagree on this point and the difference in the top contact is the most likely reason. Taken together, the results indicate that any electrical characterisation of chalcogenide films with symmetric aluminium contacts must account for Schottky barrier contributions at low voltage before attributing power-law I-U behaviour to bulk transport mechanisms. The film morphology that makes this measurement possible and the space-charge-limited regime itself are shown in Figure 14, together with the diffraction evidence that the polymorph ratio follows the growth velocity.

### 6.3. Photoconductivity and Photoresponse Spectra

The generation and transport of photoinduced carriers underlies both the sensing and the optoelectronic use of As-S materials. The photoresponse of realgar single crystals was measured by Street et al. on the same crystals and in the same semitransparent gold electrode geometry used for the drift mobility work of Section 6.2, so that the photoresponse and the dark transport refer to one and the same material [30].

The realgar photoresponse spectrum, given in Figure 13a, was recorded between 400 and 700 nm and has two features. The dominant one is a sharp rise at short wavelengths, which follows the optical absorption coefficient measured on the same crystal and reaches a maximum near 400 nm, the short-wavelength limit of the measurement [30]. This rise is the intrinsic interband response, and the gap of 2.40 ± 0.05 eV discussed in Section 5.1 was extracted from it [30]. The second feature is a weak peak at 600 nm, that is 2.07 eV, 0.33 eV below the gap. It is well-defined when the illuminated electrode is biased positive and much less prominent under the opposite polarity, which identifies the mobile species as holes, and Street et al. assigned it to free hole formation at defect or impurity sites [30]. A subgap response carried by holes and not by electrons follows the dark transport of Section 6.2, where the two drift mobilities differ by almost three orders of magnitude. Street et al. report no test of photostability, and each point of the response curve was taken with a 1/50 s pulse of low-level illumination after the sample had been depolarised, so the measurement carries no information on the realgar to pararealgar conversion.

**Figure 15:**
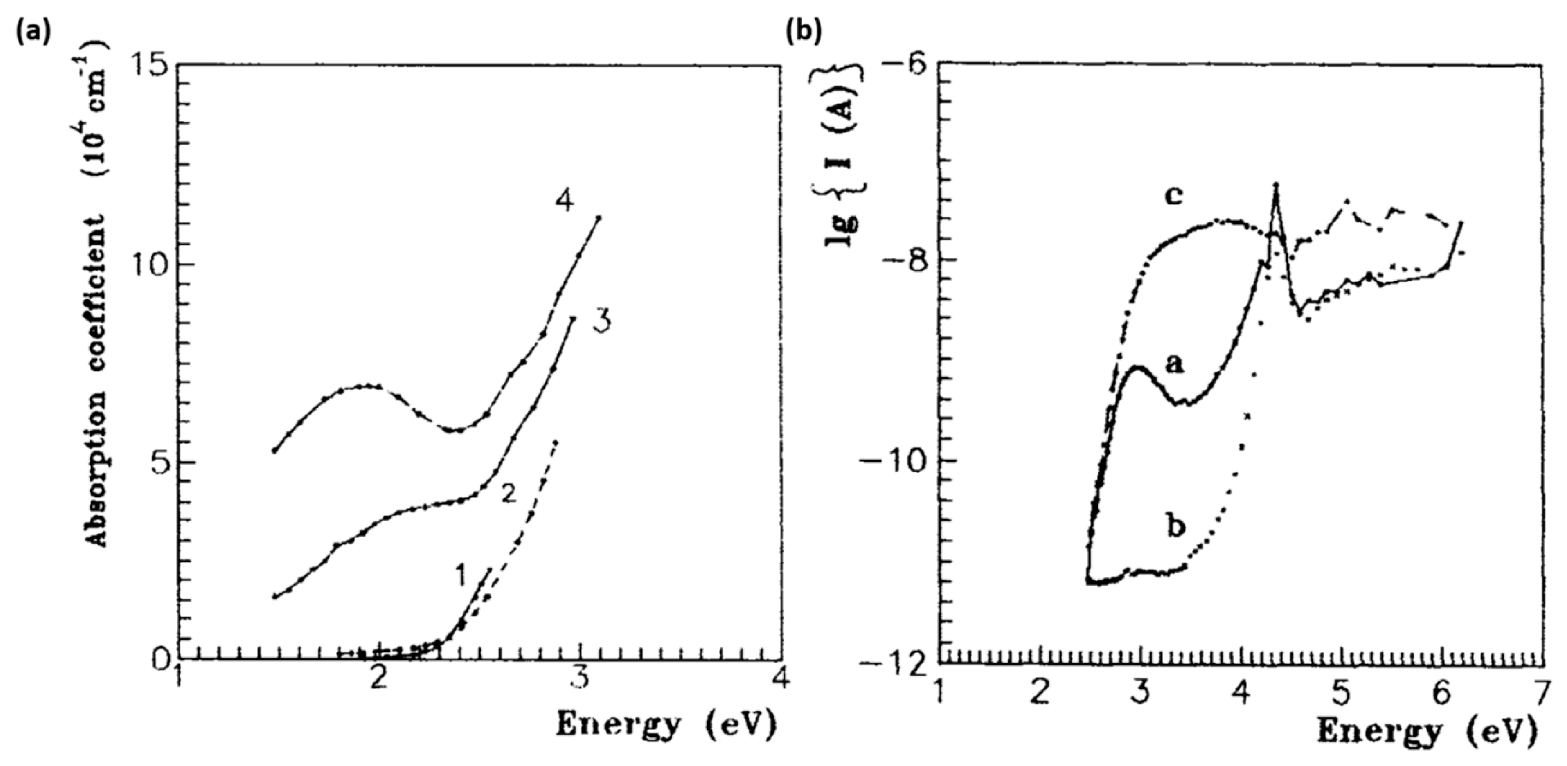
Optical and photoelectric response of thermally evaporated As_4_S_3_ films, and its dependence on the deposition conditions. (**a**) Absorption coefficient against photon energy for films grown at 7.5, 5, 4 and 0.2 µm/h on substrates held at 60, 20, 20 and 60 °C respectively. The broad band at 1.9 eV appears only in the slowest-grown, paradimorphite-rich film. (**b**) Photocurrent against photon energy for films grown at 4, 8.5 and 0.2 µm/h on substrates held at 20, 20 and 60 °C respectively. The 2.9 eV maximum tracks the 1.9 eV absorption band, while the 4.28 eV maximum is present in every film. Reproduced from [14] with permission from John Wiley and Sons, © 1996.

Dimorphite thin films show a photoresponse whose spectral shape is set by the polymorph ratio rather than by the composition. Tsiulyanu et al. measured the photocurrent of Al-As_4_S_3_-Al sandwich structures with semitransparent upper electrodes, the configuration which they report to show bipolar saturation from back-to-back Schottky barriers, the contact behaviour treated in Section 6.2 [14]. Films grown at 4 µm/h show two maxima, one in the violet at about 2.9 eV and the principal one in the ultraviolet at about 4.28 eV, while films grown at 8.5 µm/h show only the 4.28 eV maximum and no photoresponse at all between 2.5 and 3.5 eV [14]. The visible maximum therefore belongs to the α polymorph, paradimorphite, and it appears together with the 1.9 eV absorption band that Section 5.1 identifies as the optical diagnostic of the same polymorph. At the lowest growth velocity of 0.2 µm/h, where the α content is highest, the visible maximum rises to the height of the principal one and the two merge into an almost unselective response covering 3.1 to 6.2 eV [14]. The 4.28 eV maximum, at a wavelength of 290 nm, is present at every growth velocity and is therefore common to both polymorphs, as expected for a transition of the As_4_S_3_ cage, which is the same molecular unit in the two structures (Section 2.2.3). Tsiulyanu et al. offer no assignment for it, and transitions from deeper valence states to higher conduction bands remain the plausible origin. The growth velocity therefore sets the spectral selectivity of the photoresponse together with the polymorph ratio and the absorption edge structure of Section 5.1, giving an ultraviolet-selective response in the fast-grown β films and a broad response from 3.1 to 6.2 eV in the slow-grown films rich in the α polymorph, both of which are collected in Figure 15. Marian et al. later cited these spectra as the basis for selective and non-selective ultraviolet sensors made by varying the preparation technology [15], and the consequences for ultraviolet detection are discussed in Section 7.5.

### 6.4. Chemical Sensing by Chalcogenide Conductivity Modulation

The sensitivity of As-S electrical transport to the surrounding chemical environment was used by Marian et al. in the first demonstration that dimorphite thin films are gas-sensitive at room temperature [15]. The operating principle follows from the space-charge-limited transport of Section 6.2. Where the injected charge is distributed over an exponential trap distribution, the current at a given bias is governed by the energy width of that distribution, because the traps set the fraction of the injected charge that remains mobile. A species that is absorbed into the film and alters the trap distribution therefore produces a large relative change in current, an amplification that an ohmic conductor of the same resistivity does not provide.

Marian et al. exposed the smooth β-dominant films grown at 5.0 µm/h, the same films used for the transport measurements of Section 6.2, to propylamine vapour at 40 to 320 ppm carried in ambient air of 38 to 42% relative humidity and recorded both the current–voltage and the transient characteristics at room temperature [15]. The gas-induced current, defined as the difference between the current in propylamine and the current in air, is independent of the sign of the bias. The sensitivity, expressed as the gas-induced current per ppm, appears only above a bias of about 3 V, and above that threshold it increases with the applied voltage and with the vapour concentration, the concentration dependence becoming linear above 80 ppm at high bias [15]. The power law of Section 6.2 holds over almost two orders of magnitude on the positive branch above 3 V, and it is that branch which carries the analysis. Propylamine raises the exponent from the m = 3.2 measured in air to m = 3.5, and with it the characteristic energy width of the trap distribution from 0.055 to 0.062 eV, both shown in Figure 14c [15]. Marian et al. are explicit that this broadening is not the whole effect, because the vapour shifts the characteristic to higher currents as well as changing its slope, so the change in the trap distribution is neither the only nor the principal origin of the gas-induced current [15]. The two further contributions they identify are an increase of the drift mobility, which in a material where the mobility is set by multiple trapping follows from any reduction in trapping, and a decrease in the trap concentration [15]. For both they propose the same mechanism, the absorption of propylamine molecules and their trapping at charged centres, which neutralises localised states in the gap and acts as a gas-induced doping effect [15].

The transient response of the smooth films is given in Figure 14d. It follows the concentration profile applied to the chamber, but saturation of the gas-induced current is reached only after approximately 5 h [15]. Marian et al. attribute this to the high packing density of the smooth films, which leaves too few cavities for rapid uptake, so that diffusion of propylamine into the bulk of the 7 to 8 µm layer, and not adsorption at the surface, is the rate-determining step [15]. The current falls faster on switching back to air than it rises on admission of the vapour, so desorption is quicker than absorption, an asymmetry that Marian et al. describe as uncommon and leave unexplained [15]. One possibility is that uptake is limited by diffusion into a nearly cavity-free bulk while release is assisted by the concentration gradient established during exposure, but nothing in [15] separates the two. In the wider context of chalcogenide gas sensing, the dimorphite work is notable for using a stoichiometry other than the amorphous As_2_S_3_ and As_2_Se_3_ layers more commonly studied in this family and for tying the sensing response directly to a specific transport mechanism [15].

### 6.5. Photoluminescence in As-S Films

Photoluminescence in amorphous chalcogenide films is weak or absent at room temperature, where non-radiative recombination at the coordination defects of the disordered network dominates the decay of photoexcited pairs. Nezhdanov et al. found no photoluminescence at all from as-deposited PECVD As-S films across the 500 to 1100 nm range they examined and showed that localised laser irradiation crystallises micro- and nanoscale inclusions within the film and that these inclusions emit [6].

Five PECVD compositions, from As_35_S_65_ to As_55_S_45_, were irradiated at room temperature with a 473 nm continuous-wave laser for 60 s at 0.7 mW, the lowest power at which emission appeared and one chosen to avoid evaporating the film [6]. The photon energy of 2.62 eV falls within the intrinsic absorption of every composition, which is the condition under which photoinduced effects in chalcogenides are strongest [6]. After irradiation all five films emitted a broad band with a full width at half maximum of about 0.4 eV, whose maximum moves from approximately 1.8 eV in the sulphur-rich As_35_S_65_ film to approximately 2.05 eV in the arsenic-rich As_55_S_45_ film, monotonically through the three intermediate compositions [6]. That width is essentially the 0.36 eV of the bound-exciton band of bulk orpiment discussed below, so it calls for no broadening mechanism specific to the inclusions.

The phase of the photogenerated inclusions was determined by micro-Raman spectroscopy at the same irradiated spots. In the As_55_S_45_ film the spectrum narrowed between 280 and 420 cm^−1^ and gained intensity at 171, 202 and 273 cm^−1^ after irradiation, which identifies crystalline dimorphite [6]. In the As_52_S_48_ film the 273 cm^−1^ band also grew, which the authors read as a rise in the As_4_S_3_ fraction and not as pararealgar formation, since the As_4_S_4_ units in these films are already in the pararealgar form [6]. For the three sulphur-richer compositions no phase could be assigned, because the luminescent background and the drop in signal intensity after irradiation left the Raman spectra unable to distinguish orpiment, realgar, pararealgar, dimorphite and uzonite [6].

**Figure 16:**
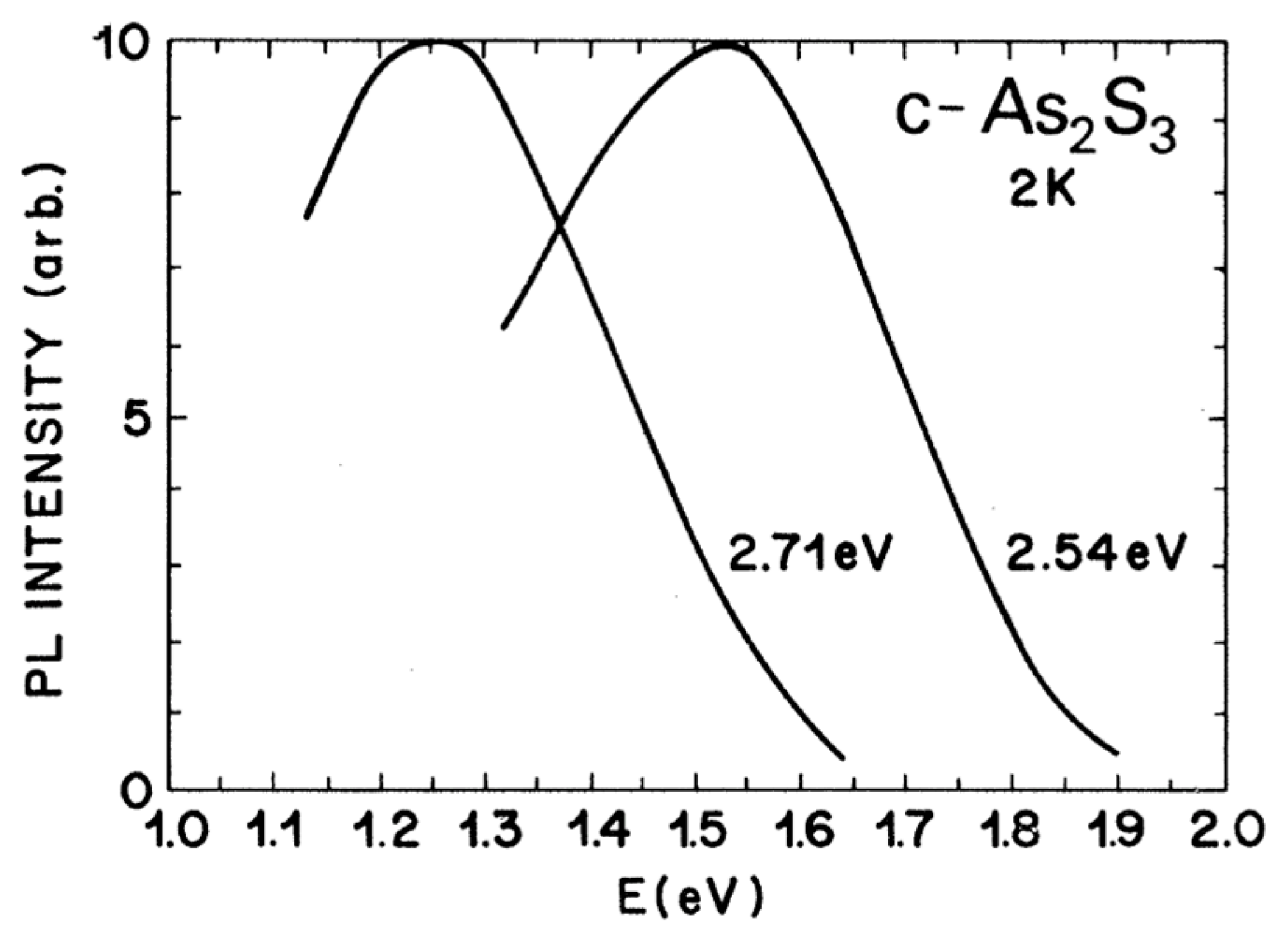
Photoluminescence spectra of crystalline orpiment at 2 K for two excitation energies, 2.54 eV exciting the defect-related bound-exciton band and 2.71 eV exciting the self-trapped-exciton band, with the luminescence plotted in photons per square centimetre per electronvolt. Reprinted figure with permission from [88]. Copyright 1981 by the American Physical Society.

The film emission can be set against the two photoluminescence bands of bulk crystalline orpiment resolved by Murayama et al. in natural crystals at 2 K [88]. A defect-related bound-exciton band peaks at 1.53 eV under 2.54 eV excitation, with a Gaussian width of 0.36 eV and a Stokes shift of about 1.05 eV from the impurity band that excites it, and a second band at 1.25 eV is induced by band-to-band excitation at 2.71 eV and is assigned to a self-trapped exciton [88]. Separating the two resolved a conflict in the earlier literature, where some studies had reported a band near 1.26 eV at 77 K and others a main band at 1.6 eV, differences that Murayama et al. attributed to the two distinct processes and not to variation in crystal quality [88]. The comparison with the films is indicative and not quantitative, since the bulk bands were measured at 2 K while the film spectra were recorded at room temperature, and both bulk bands lie at or below the low-energy limit of the range plotted in [6]. Both bands are shown in Figure 16, the 1.53 eV band under 2.54 eV excitation and the 1.25 eV band under 2.71 eV excitation. The discussed peak positions hold for luminescence plotted in photons per square centimetre per electronvolt, the units of the figure, the high-energy band lying at 1.59 eV when the same data are plotted per unit power.

Nezhdanov et al. note that the photoluminescence of bulk arsenic sulphide crystals is generally assigned to radiative recombination of bound excitons localised at defects, assume the same nature for the film emission, and attribute the difference in peak position to the crystalline phase being present as micro- and nanoscale inclusions, whose confinement should alter the energy structure of the bound excitons [6]. The measured peak positions place a constraint on any such assignment that the paper does not draw. The emission maximum moves in the opposite sense to the optical gap of the host film, which decreases from 2.417 eV in As_35_S_65_ to 1.875 eV in As_55_S_45_ (Section 5.1), so that the emission lies roughly 0.6 eV below the gap in the sulphur-rich film and slightly above it in the arsenic-rich one. Emission above the gap of the surrounding matrix cannot originate in that matrix, which is direct support for assigning the luminescence to the crystalline inclusions and not to the amorphous host.

The crystallisation itself is attributed to the combined photoinduced and thermal action of the focused beam. A one-dimensional heat-equation estimate places the temperature at the exposure point at the crystallisation temperature of these films for powers between 0.7 and 4 mW, so photoinduced and thermal effects operate together at the power used [6]. The phase that forms is set by the structural units already present in the as-deposited film, dimorphite appearing where As_4_S_3_ cages dominate [6]. The Urbach energy before irradiation, 0.071 to 0.087 eV [6], varies little across the composition series, so the disorder of the starting network is not what distinguishes one composition from another here.

The tunability of the PL energy between 1.8 and 2.05 eV by compositional adjustment, combined with the ability to define luminescent microzones by controlled laser writing, allows spatially addressed photoluminescent elements in As-S thin-film platforms. Integration with the waveguide and non-linear optical properties reviewed in Section 5.3 and Section 5.5 suggests that As-S films could serve as multifunctional photonic layers in which waveguiding, non-linear processing, and locally addressable luminescent markers coexist in a single deposited film, adjustable by composition and processing history.

## 7. Device Applications

The combination of properties reviewed in Section 4, Section 5 and Section 6, wide transparency window, the highest third-order optical non-linearity of any established waveguide material, large and reversible photoinduced refractive index change, high dark resistivity, phase-tunable photoconductivity, and SCLC-governed gas sensitivity, makes binary As-S thin films applicable to several device families. This section reviews the principal device demonstrations and applications that have been realised to date, organised by application class, covering integrated non-linear photonic circuits, holographic recording and wavefront manipulation, surface plasmon resonance sensors, laser-written micro-optical elements, and optoelectronic sensing devices.

### 7.1. Integrated Non-Linear Photonic Circuits

The long-recognised potential of chalcogenide glasses for all-optical signal processing was first translated into integrated device demonstrations using As_2_S_3_ thin-film waveguides. The critical enabling developments, ultralow-loss waveguide fabrication, dispersion engineering, and CMOS-compatible lithographic processing, were reviewed in Section 5.3 and Section 5.5. Here the resulting device-level capabilities are summarised. The non-linear parameter and the figure of merit of the several waveguide geometries are compiled in Section 5.5. The device-relevant consequence is that every As_2_S_3_ configuration supports net non-linear phase accumulation and does so without the free-carrier effects that hold silicon nanowires below the threshold [1].

The most important demonstration of this advantage was the 1.28 Tbit/s optical time-division multiplexing (OTDM) demultiplexing experiment performed using a 7 cm As_2_S_3_ rib waveguide with γ ≈ 9900 W^−1^km^−1^ [1]. In this experiment, four-wave mixing within the As_2_S_3_ waveguide, driven by a continuous-wave pump co-propagating with a 1.28 Tbit/s 800 fs return-to-zero signal train, generated an idler at the difference frequency that accurately reproduced a single 10 Gbit/s tributary, demonstrating error-free 128:1 demultiplexing. The peak power loadings reached 10–20 MW cm^−2^ without measurable photodamage or photosensitivity-induced index drift, a practical confirmation that As_2_S_3_ withstands the high optical intensities intrinsic to non-linear signal processing at telecom data rates [1]. The propagation losses and the loaded resonator quality factors reached on this platform are given in Section 5.3 [3]. At this loss level, sufficient circulating intensity is accessible with milliwatt-class pump lasers to drive stimulated Raman scattering, parametric amplification, and supercontinuum generation at threshold powers compatible with chip-scale integration. The laser-induced damage thresholds quoted in Section 5.3 leave a wide handling margin for both ultrafast and continuous-wave non-linear operation [3].

**Figure 17:**
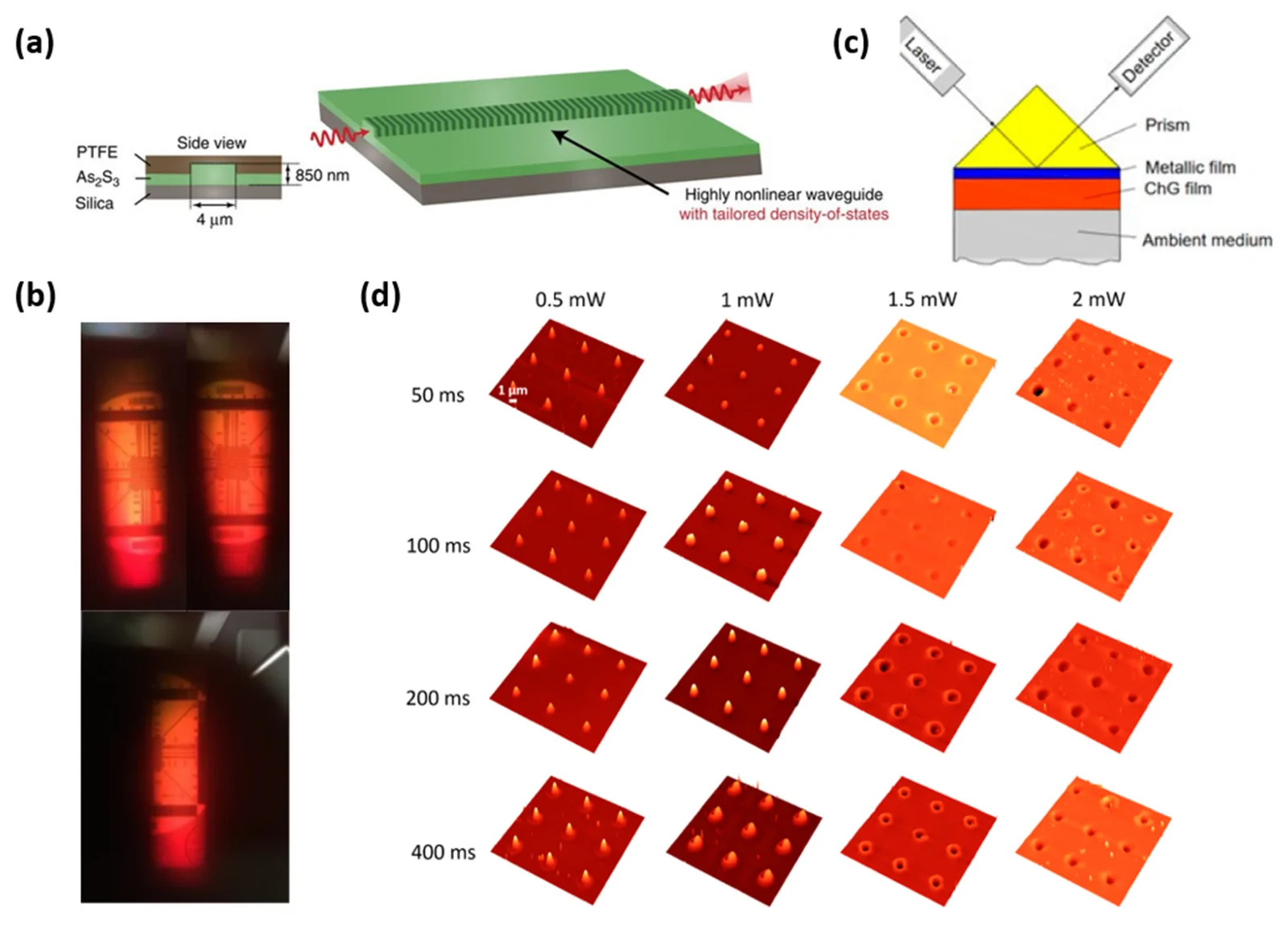
Devices realised in As-S thin films. (**a**) As_2_S_3_ rib waveguide used for stimulated Brillouin scattering, 850 nm by 4 µm on a silica substrate with a polytetrafluoroethylene over-cladding, shown in cross-section and as the Bragg-grating structure whose tailored density of states enhances or inhibits the interaction. (**b**) Red test image transmitted through a periscope formed by an in-coupling surface relief hologram and an out-coupling volume hologram recorded in As_2_S_3_ films, shown as three sub-images of the field of view. (**c**) Concept of the four-layer surface plasmon resonance structure, in which the chalcogenide film, amorphous As_2_S_3_ in [9], acts as the plasmonic waveguide. (**d**) Atomic force micrographs of femtosecond-laser written relief on a bare As_2_S_3_ film as a function of laser power and exposure time, showing photoexpansion below the modification threshold and photoevaporation above it. Panel (**a**) reproduced from [89], panel (**b**) from [10], panel (**c**) from [9] and panel (**d**) from [41], all under Creative Commons Attribution 4.0 International Licences, © 2015, © 2022, © 2023 and © 2024 the authors.

Stimulated Brillouin scattering is the other non-linear process for which As_2_S_3_ waveguides have become the reference on-chip platform, the Brillouin shift in this glass being about 7.7 GHz [89]. Merklein et al. used a 6.8 cm As_2_S_3_ rib waveguide of 850 nm by 4 µm cross-section on silica, over-clad with polytetrafluoroethylene, in which an effective mode area of 2.3 µm^2^ and the Brillouin gain of the glass give a measured threshold of 0.6 W [89]. Writing a Bragg grating along the waveguide places the Stokes wave either at the slow-light band edge of the stopband or inside it, which turns one structure into either an enhancer or a suppressor of the interaction. In the first case the enhancement cascades into a Brillouin frequency comb of fifteen lines, and in the second the Stokes wave is suppressed and the waveguide operates free of stimulated Brillouin scattering at twice the threshold power [89]. Control in both directions is what the platform needs, since Brillouin gain is the resource in microwave photonics and the same gain is the power ceiling on every other non-linear function sharing the circuit. The waveguide is shown in Figure 17a.

One important practical consideration for photonic integration is the sensitivity of As_2_S_3_ refractive index to temperature. The thermo-optic coefficient and its ratio to that of silicon are given in Section 5.4, and the consequence for device design is that As_2_S_3_ passive elements drift far less with temperature than their silicon counterparts [3]. The dispersion profile of As_2_S_3_ waveguides in the normal or anomalous regime can be tuned by waveguide geometry to provide zero-dispersion wavelengths anywhere in the 1–3 µm range, enabling soliton propagation and mid-infrared supercontinuum generation with octave-spanning output from compact on-chip sources [3]. Taken together, these results establish As_2_S_3_ as the preferred chalcogenide platform for passive and non-linear photonic integration from the near-infrared through the mid-wave infrared, combining the largest FOM of any established waveguide material with a mature fabrication technology and molecular-scale uniformity achievable by optimised physical vapour deposition.

### 7.2. Holographic Recording and Wavefront Manipulation

The large and reversible photoinduced refractive index change of amorphous As-S films, whose magnitude is given in Section 5.6, offers a route to hologram recording without the photopolymers, silver halides or photoresists that conventional holographic media require. Borisov et al. assessed thermally evaporated As-S and Ge-S films of 0.7 to 1 µm thickness on glass substrates as recording media for augmented reality waveguide combiners, combining rigorous coupled-wave modelling of what the waveguide demands of the material with holographic recording experiments [10]. The modelling fixes the targets. A single-exposure holographic optical element needs a film of refractive index above 1.8, an index modulation above 0.1 and a thickness between 0.5 and 1 µm, and a two-exposure element needs 2.1, 0.13 and 1.65 to 2 µm respectively [10]. A field of view beyond 50 degrees is reachable with a suitable substrate index, and amorphous As_2_S_3_ meets the index requirement with room to spare [10]. The index modulation is the binding constraint, since Δn above 0.1 is reached only at the saturation fluences described in Section 5.6.

Recording on four As_2_S_3_ films of 1 µm thickness was performed by two-beam interference at 457 nm, with the diffraction efficiency monitored during exposure by a 650 nm laser diode, and the polarisation of the writing beams selected which of two gratings of 430 nm period formed [10]. Linearly polarised beams at 20 mW/cm^2^ wrote a volume phase grating through the photodarkening index modulation and reached a diffraction efficiency of 1% in three minutes. Circularly polarised beams at 120 mW/cm^2^ wrote a surface relief grating through photoinduced mass transfer and reached 10% only after twelve hours [10]. The relief route therefore buys a factor of ten in efficiency at about 5.2 kJ/cm^2^ against 3.6 J/cm^2^, a factor of roughly 1400 in dose. The relief diffracts as a phase grating and not an amplitude one, its modulation depth being the relief height multiplied by the index step between the film, n = 2.53 for As_40_S_60_ [77], and air. Assembled into a periscope, an in-coupling relief hologram of 10% efficiency and an out-coupling volume hologram of 1% efficiency transmitted a red test image through the waveguide with good resolution and brightness, shown in Figure 17b, although only the red and green parts of the field passed, the blue being lost to the absorption edge of these films [10]. Diffraction efficiencies of about 1% or less except by relief recording, doses of several kilojoules per square centimetre for the relief route, and the need to place the recording wavelength precisely at the absorption edge of each composition led them to conclude that holographic recording in chalcogenide films is at present ineffective for producing such elements and that their results are a basis for further development rather than a working technology [10].

The reversibility of photodarkening, recoverable by annealing without chemical treatment, is at once the advantage and the limitation of these films for holography. It is an advantage because a photodarkened film can be thermally erased and re-exposed, which a photopolymer hologram fixed by a post-exposure flood cure cannot be. It is a limitation because only the reversible component is available for rewriting, and Section 5.6 shows that in as-deposited evaporated As_2_S_3_ that component is the smaller one, the polymerisation of the molecular cage units of the as-deposited film contributing an irreversible Δn of about 0.10. A rewritable medium must therefore be pre-annealed, which spends the irreversible part of the index change before any recording begins. The thermal budget of the reversible component in As-S films has not been reported, and neither the hologram lifetime nor its dependence on storage temperature was measured in [10].

The composition dependence is better established. Zakery et al. measured the photodarkening of thermally evaporated As-S films across the glass-forming range under 514.5 nm illumination at 200 mW/cm^2^ and found that it grows with arsenic content, becoming negligible around As_30_S_70_ and undetectable at As_28_S_72_ [77]. The induced index change rises from 0.095 at As_35_S_65_ and 0.1 at As_33_S_67_ to 0.11 at As_40_S_60_, the arsenic-richest composition measured, where the film index is 2.53 [77]. They attribute the trend to the photoinduced formation of As-As bonds at the expense of As-S bonds, which is favoured where As-S bonds are most numerous [77]. Set against the requirement of Δn above 0.1 from the modelling in [10], this confines the useful composition window for holography to the arsenic-rich end of the As-S glass-forming range, at or near the stoichiometric As_40_S_60_. Whether Δn continues to rise beyond that composition was not measured, so As_40_S_60_ is the upper limit of the measured composition range and an optimum has not been established. Reversibility across that window is treated in Section 5.6 [8]. The comparison with the phase-change media is instructive. At 405 nm, crystallisation of Ge_2_Sb_2_Te_5_ changes the refractive index by −1.20 and the absorption coefficient by +1.05 [4], so the index change is an order of magnitude larger than anything photodarkening produces in As-S, but it is accompanied by an absorption change of nearly the same size. The As-S index change is small and close to purely refractive, since it is induced below the absorption edge. Whether that trades favourably in a thin recording layer is untested, as no comparison of the two materials in a holographic geometry has been reported.

### 7.3. Surface Plasmon Resonance Sensors

Surface plasmon resonance sensors conventionally use the three-layer Kretschmann configuration of a prism, a gold film of 40 to 50 nm and the analyte, in which the position and the width of the resonance are fixed by the optical constants of the metal and the index of the prism, and only the position responds to the analyte. Popescu et al. reviewed a four-layer variant in which a transparent high-index film is deposited on the outer face of the gold, so that the stack runs prism, gold, chalcogenide film, analyte, and the chalcogenide becomes a planar plasmonic waveguide in contact with the medium being sensed [9]. Incident light couples into a waveguide mode of that film through the evanescent field penetrating the semitransparent gold layer. The high index of amorphous As_2_S_3_, taken as 2.45 through most of the calculations in [9] and measured by ellipsometry as 2.852 − 0.019i at 514 nm for the films used experimentally, confines the field near the outer surface, and the degree of confinement is set by placing the waveguide mode relative to its cut-off, which is done through the film thickness [9]. The gain over the three-layer configuration is therefore adjustability rather than a fixed improvement, since the resonance angle, the resonance width, the penetration depth and the sensitivity all become design variables through the film thickness and the choice of prism, where the three-layer structure offers no such freedom [9]. What limits the sharpness of the resonance is absorption in the chalcogenide, an absorption coefficient of 100 cm^−1^, equivalent to only 1% loss in a 1 µm film, being enough to broaden the resonance contour appreciably [9]. The four-layer structure is shown in Figure 17c. The results collected in [9] are transfer-matrix simulations throughout, with the exception of the hysteresis measurement described below.

The sensitivity depends strongly and non-monotonically on the film thickness. For alcohol identification at a working wavelength of 1550 nm the calculated shift of the resonance angle per refractive index unit was 4.90°/RIU for an 800 nm As_2_S_3_ film, 13.30°/RIU at 1000 nm and 8.40°/RIU at 1100 nm, so a 300 nm change of thickness moves the sensitivity by a factor of about 2.7 [9]. From these figures the authors estimate that refractive index changes below 10^−4^ can be distinguished, a limit set by the precision of the angle measurement and the stability of the source rather than by the structure itself [9]. The corresponding biosensing calculation targets β-galactosidase, the marker enzyme produced by live Escherichia coli, which is measured in place of the bacteria themselves. For a BK7 prism, 45 nm of gold and a 1000 nm As_2_S_3_ film of index 2.4693 against an aqueous solution, the resonance angle moves from 61.94° for pure water to 62.04° for a 1% enzyme solution [9]. Two further properties of the four-layer geometry follow from the same calculations. The optimum gold thickness is near 50 nm in the visible but 40 to 45 nm in the near-infrared, and resonance can be excited in both p and s polarisation, where the three-layer configuration supports only p [9]. A prism of index higher than the film, such as gallium phosphide, gives resonance over a continuous range of film thicknesses, while a BK7 prism, whose index lies far below that of the film, still couples but only for film thicknesses within a restricted window [9].

The one experimental result in [9] is an optical hysteresis. A structure of BK7, gold and a thermally evaporated As_2_S_3_ film, with air as the fourth medium, was illuminated through the prism by a continuous-wave argon laser at 514 nm polarised in the plane of incidence, with the incident power varied between 1 and 20 mW [9]. The resonance angle at low power was 41.13°. With the angle then held fixed at 40.8°, on the low-angle branch of the dip, the output power traced a hysteresis loop between 10 and 11 mW as the incident power was ramped up and back down, and no loop appeared when the fixed angle was set on the high-angle branch instead [9]. The effect is self-induced, the illumination changing the refractive index of the As_2_S_3_ film and the changed index in turn altering the coupling, and the authors present it as a route to bistable optical devices operating at low intensity. For sensing it is a constraint, since it places an upper bound on the interrogation power before the film begins to drift.

The practical limitation of the geometry follows from its own design, since the chalcogenide rather than the gold is the layer exposed to the analyte. Ref. [9] reports that the film surface deteriorates in use and handles this by carrying the gold and chalcogenide layers on a separate substrate coupled to the prism with immersion oil, so that the chipset can be replaced while the prism is retained, noting that such chipsets wear out after several applications. The As_2_S_3_ films were prepared by thermal evaporation at 6.6 × 10^−4^ Pa in a setup modified to suppress droplet formation and surface roughness, both of which would broaden the resonance [9]. Chemical durability of the exposed chalcogenide surface in aqueous media, and its resistance to biofouling, are not addressed in [9] and remain the open questions for this configuration as a biosensor platform.

### 7.4. Laser-Written Micro-Optical Elements

The localised photoexpansion of amorphous As_2_S_3_ films under a focused laser beam provides a maskless direct-write route to micro-optical elements, principally lenslets and diffractive structures, with no chemical processing and no material removal. Two studies by Velea et al. set the process window for 800 nm femtosecond writing on As_2_S_3_ films grown by pulsed laser deposition. In the first, films of about 2 µm thickness were deposited by a KrF excimer laser at 1.5 J/cm^2^ and irradiated with 15 fs pulses at 800 nm from an 80 MHz oscillator delivering 5 nJ per pulse, focused by a 0.5 numerical aperture objective to a spot of about 3 µm in diameter, each site being exposed for 300 ms [90]. Below 6 mW the surface was unaltered. Between 8 and 20 mW the irradiated sites rose into hillocks, and above that the beam opened holes which deepened and widened with power until ablation dominated at 50 mW [90]. Photoexpansion is reported here as the relative volume change ΔV/V_0_, the volume of material standing above the original surface divided by the volume of an irradiated cylinder whose height is the film thickness, and it reached 5.11% at 18 mW, where the hillock height was 210 nm, before falling back to 4.86% at 20 mW [90]. The radius of the irradiated area grew over the same range, from 0.64 µm at 8 mW to 0.84 µm at 18 mW [90]. The hillocks have an asymmetric double sigmoidal cross-section, which the authors relate within the cluster model to a larger expansion along the beam direction and to photoinduced optical anisotropy, and they propose them as lenslets for the planar geometry of optical circuits on the precedent of earlier lenslets written by the same effect on the ends of optical fibres, which were active in the red and near infrared with focal lengths near 10 µm [90].

The second study reduced the film thickness to 1 µm, added a 100 nm gold underlayer in glass/Au/As_2_S_3_ heterostructures, and treated the exposure time as an independent variable [41]. With the same 15 fs source, the laser power was varied from 0.125 to 2 mW, corresponding to fluences of 88.5 to 353.9 µJ/cm^2^ and intensities of 5.9 to 23.6 MW/cm^2^, with about 4 × 10^6^ pulses delivered per spot in 50 ms and 32 × 10^6^ in 400 ms [41]. The transition from photoexpansion to photoevaporation falls between 1 and 1.5 mW and does not move with exposure time, and at 1 mW with 400 ms the two regimes coexist in a single feature that is half hillock and half hole [41]. The maximum photoexpansion on bare As_2_S_3_ was 5.2%, reached either at 1 mW with 50 ms exposure or at 0.5 mW with 100 ms, the two settings delivering an equivalent cumulative dose [41]. This is comparable to the 2.45% maximum reported for amorphous Ga_2_S_3_ films, but it is obtained at 1 mW against the 100 mW required for Ga_2_S_3_, two orders of magnitude lower [41]. The greater low-power sensitivity of As_2_S_3_ is attributed to its as-deposited structure of As_4_S_6_ hard-sphere molecules, which polymerise and cross-link under heating or illumination towards the network structure of the bulk glass, and to the permanent component of the photoexpansion being both larger in magnitude and slower in kinetics than the transient response [41]. The structural mechanism proposed for this expansion is a redistribution of electrical charge onto the sulphur atoms of each molecular cluster under femtosecond illumination. The resulting increase in electrostatic repulsion between sulphur atoms expands the cluster by network reconfiguration without breaking any bonds, and the van der Waals separation between neighbouring clusters increases in parallel, which accounts for an expansion of about 5% that persists after the beam is switched off [91]. Across the photoexpansion regime the hillocks were 0.50 to 0.77 µm wide and 52 to 112 nm high [41].

The gold underlayer changes the response in both directions. The onset of modification arrives sooner, at 0.125 mW after 100 ms against 0.125 mW after 400 ms on the bare film, so what falls is the exposure time and not the power, while the transition between photoexpansion and photoevaporation is barely affected [41]. The maximum expansion, on the other hand, drops to 0.8%, obtained at 0.25 to 0.5 mW with 100 ms, so femtosecond writing at 1 mW and 400 ms is more than four times as effective on the bare film as on the heterostructure [41]. The earlier onset is ascribed to enhancement of the electromagnetic field at the interface by localised surface plasmons, while for the reduced maximum two explanations are offered without a decision between them, an alteration of the optical and electronic properties of the As_2_S_3_ by the adjacent metal, and mechanical restriction of the deformation by a gold layer stiffer than the chalcogenide [41]. A parallel comparison with continuous-wave writing in the same films confirms that the femtosecond regime is the more efficient one for photoexpansion [41]. Above the transition, irradiation at 1.5 mW for 400 ms leaves a volcano-shaped feature, a cone 41 nm high around a hole 49 nm deep, and at 2 mW the cone shrinks while the hole deepens, reaching 133 nm at 100 ms [41]. At the much higher powers of the earlier study the composition changed as well, sulphur being partly released and realgar, As_4_S_4_, appearing in the X-ray diffraction pattern of the irradiated film [90]. The relief written on bare As_2_S_3_ across the power and exposure range is shown in Figure 17d.

These results define a practical process window for laser-written As_2_S_3_ micro-optics. Modification begins at 0.125 mW, and up to 1 mW the response is pure photoexpansion, giving smooth convex lenslets 0.5 to 0.8 µm wide and up to about 110 nm high, which suits coupling and beam shaping. Between 1 and 1.5 mW the two regimes overlap and the features become mixed. Above 1.5 mW photoevaporation dominates and produces the holes and craters needed for diffractive elements, at the cost of sulphur loss and partial crystallisation at the highest powers. The absence of any wet-chemical processing, the compatibility of femtosecond direct writing with pre-patterned substrates, and feature widths of half a micrometre from a focal spot six times larger, which [41] attributes to two-photon absorption confining the modification to the centre of the intensity distribution, make As_2_S_3_ photoexpansion a workable maskless route for multielement planar photonic systems.

### 7.5. Optoelectronic Sensing Devices

Two optoelectronic applications have been proposed for dimorphite films, ultraviolet detection and chemical sensing. Both rest on the measurements reviewed in Section 6.3 and Section 6.4, which are not repeated here, and the two differ in how far they have been taken.

Ultraviolet detection follows from the photoconductivity spectra of Section 6.3. A fast-grown β-dominant film responds in a single ultraviolet band near 4.28 eV and not at all between 2.5 and 3.5 eV, a film containing the α polymorph gains a second maximum in the violet near 2.9 eV, and the slowest-grown films lose the distinction altogether and respond across 3.1 to 6.2 eV. The growth velocity therefore selects between a spectrally selective ultraviolet response and a broadband one without any change of composition, which is the basis on which Marian et al. state that selective and non-selective ultraviolet sensors can be fabricated from these films [15]. No such detector has been built or characterised, and the device remains a proposal. Chemical sensing is the one application that has been demonstrated. The space-charge-limited transport of Section 6.2 gives the film an internal amplification above the injection threshold, so that a change in the trap distribution caused by absorbed propylamine appears as a large relative change in current, and Section 6.4 gives the measured response [15]. Neither application has an equivalent anywhere else in the As-S literature and no dimorphite device has been made with modern lithography or in a geometry suited to sensor array integration.

## 8. Conclusions and Outlook

Binary arsenic sulphide offers three thermodynamically stable compounds in a single binary system, and each of the three behaves differently as a thin film. Orpiment (As_2_S_3_, 40 at. % As) is a network former that deposits amorphous by every vacuum method and dominates the mature and integrated-device literature of the system, while the devices demonstrated in the arsenic-rich compositions are the As_4_S_3_ photodetectors and amine sensors described in Section 6 and Section 7. Realgar (As_4_S_4_, 50 at. % As) is a molecular crystal whose thin films are chemically unstable under visible light in air. Dimorphite (As_4_S_3_, 57.1 at. % As) is the arsenic-richest stable phase, it sublimes congruently [5], it deposits microcrystalline and not amorphous by thermal sublimation [14], and it is the only phase in this system for which a deposition parameter, the growth velocity, has been shown to set the polymorph ratio. The organising principle that emerges from Section 2 to Section 7 is the competition between network and cage, and the position of a given film in that competition is fixed first by composition and then by the kinetics of the deposition method.

The deposition method analysis in Section 3 supports a phase-specific choice of method and not a single preferred technique. Congruent sublimation makes thermal evaporation the most direct method for As_4_S_3_ stoichiometry [5,14]. As_2_S_3_ evaporates incongruently from the melt, and for that composition pulsed laser deposition in the mode-locked regime gives near-stoichiometric transfer, with a sulphur deficiency of about 1.5 at. % and an rms roughness of 0.42 nm (Table 5) [40]. Radio-frequency magnetron sputtering loses sulphur preferentially, an effect that follows from the general behaviour of multicomponent targets [36] and for which no As-S-specific calibration has been published, so film composition has to be verified against the target for every parameter set. Plasma-enhanced chemical vapour deposition is the only method that covers the full binary continuously in one system, and the bandgap moves from about 2.42 eV at As_35_S_65_ to about 1.87 eV at As_55_S_45_ [6,7]. Solution processing reaches large areas at low cost but leaves residual solvent, and waveguide losses remain near 9 dB/cm, two orders of magnitude above the vacuum-deposited films [3].

Three results carry across the whole composition range. First, Raman spectroscopy is the working phase diagnostic for these films, since it separates network, cage and polymorph at volume fractions well below the detection limit of grazing-incidence diffraction, and the diagnostic bands are now assigned for every phase [13,26]. Second, photodarkening in annealed a-As_2_S_3_ is an intermediate-range-order effect and not a bond-breaking effect, since the homopolar bond fraction changes by only about half a percentage point while the refractive index moves by up to 0.03 at 633 nm [8]. Third, the transport mechanism changes with the phase and not only with the disorder, from band-like hole conduction with 12 cm^2^V^−1^s^−1^ in realgar single crystals [30] to space-charge-limited conduction through an exponential trap distribution in dimorphite films [15].

Regarding the gaps, the entire published thin-film record of As_4_S_3_ is based on two experimental papers [14,15]. No refractive index has been published for either polymorph in thin-film form, and without it no waveguide design, ellipsometric model or interference analysis is possible for this phase. Every crystalline As_4_S_3_ film reported to date is microcrystalline and contains both polymorphs, with a morphology that ranges from fibrillar and porous at a growth velocity of 0.2 µm/h to compact at 5.0 µm/h [15]. Spectroscopic ellipsometry on such a film gives an effective medium response that combines the indices of the two polymorphs, the void fraction and the surface roughness, and the index of one polymorph can be extracted only with an independent determination of the polymorph ratio and the porosity. A reliable measurement therefore requires a dense film of a single polymorph, and no such film has been reported. No non-linear coefficient has been measured, although the cage carries three As-As bonds against two in realgar and none in As_2_S_3_, which is the structural argument for expecting a larger third-order response. No photodarkening study has been run on a single-polymorph film, no medium-range-order probe has been applied to the amorphous dimorphite-composition film, and no peer-reviewed Raman assignment exists for either polymorph as a film. The dimorphite to paradimorphite transition has been measured only on bulk material, between 131 and 149 °C [18], and not in a film of any thickness. The two polymorphs contain the same As_4_S_3_ cage in different packing orientations (Figure 2f,g), and the transition enthalpy is small, 1.44 kJ/mol [18]. In a film, the difference in surface energy between the two packings and the strain imposed by the substrate can be of comparable magnitude, so a shift of the transition temperature is expected. The measurement requires calorimetry or temperature-resolved diffraction on films of controlled thickness, with the starting polymorph ratio determined for each film, since that ratio depends on the growth velocity. Beyond As_4_S_3_, three further gaps are worth naming. The competition between crystalline phases during annealing of arsenic-rich films has not been mapped systematically as a function of temperature, time and atmosphere. The stability of realgar-composition films in ambient air is governed by an oxygen-initiated autocatalytic transformation whose rate in the thin-film geometry has never been quantified [11]. And the preferential sulphur sputtering coefficient for As-S targets, on which every sputtering campaign depends, has no dedicated published calibration. Table 9 sets out which properties have been measured for each phase and which have not.

No sputtering study of As_4_S_3_ has been published and no annealing study of a vacuum-deposited film of this composition exists, so whether a sputtered and annealed layer can reach phase-pure dimorphite and reproduce the photoconductivity and gas response established for vacuum-sublimed films [14,15] is an open question. The measurements that would close the largest gaps are spectroscopic ellipsometry for the dispersion of both polymorphs, Raman on annealed films against the 337 cm^−1^ and 224 cm^−1^ diagnostic bands [13], and photoconductivity spectra on films of controlled α to β ratio. Each of these is accessible with standard laboratory instrumentation and none requires a synchrotron. Regarding the applications, the dual-band photoresponse at 2.9 eV in the violet and 4.28 eV in the ultraviolet and the amine sensitivity of the space-charge-limited regime have been reported only for dimorphite within the As-S family, and both have been demonstrated only in micrometre-thick evaporated films that predate modern thin-film patterning. Reproducing them in sputtered submicrometre films on patterned substrates is the concrete step that would establish As_4_S_3_ as a device material and not only a mineral phase.

## Figures and Tables

**Table 1:** Thermal properties of the stable binary As-S compounds [5].

Compound	Formula	As (at. %)	Melting Point	Notable Transitions
Orpiment	As_2_S_3_	40.0	~307 °C (580 K); sublimes congruently at equilibrium	Glass transition 211 °C (bulk glass); 185–200 °C (annealed films) [5]
Realgar	As_4_S_4_	50.0	~309 °C (582 K); sublimes congruently at equilibrium	Realgar to pararealgar (photoinduced, RT)
Dimorphite	As_4_S_3_	57.1	~211 °C (484 K); melts and sublimes congruently	Dimorphite to paradimorphite at 149 °C, paradimorphite to plastic phase at 159 °C

**Table 2:** Crystallographic data for As_4_S_3_ polymorphs.

Polymorph	IMA Mineral Name	Stability	a (Å)	b (Å)	c (Å)	Z	Ref.
Low-T (β)	Dimorphite	Stable at room temperature, converts to paradimorphite at 404 to 422 K (131 to 149 °C)	11.21	9.90	6.58	4	[22]
High-T (α)	Paradimorphite	Stable between 422 and 432 K (149 to 159 °C), retained at room temperature by quenching	9.1577(7)	8.0332(6)	10.2005(8)	4	[13,28]

**Table 3:** Crystallographic summary of all stable binary As-S phases.

Phase	Formula	Space Group	a (Å)	b (Å)	c (Å)	β (°)	Z	Ref.
Orpiment	As_2_S_3_	P2_1_/n (No. 14)	11.475	9.577	4.256	90.68	4	[20]
Anorpiment	As_2_S_3_	$P\bar{1}$ (No. 2)	5.7577	8.7169	10.2682	α = 78.15, β = 75.82, γ = 89.86	4	[21]
Realgar	As_4_S_4_	P2_1_/n (No. 14)	9.325	13.571	6.587	106.38	4	[20]
Pararealgar	As_4_S_4_	P2_1_/c (No. 14)	9.909	9.655	8.502	97.29	4	[12]
Alacranite	As_8_S_9_	P2/c	9.942	9.601	9.178	101.94	2	[25]
Dimorphite (low-T)	As_4_S_3_	Pnma (No. 62)	11.21	9.90	6.58	90	4	[22]
Paradimorphite (high-T)	As_4_S_3_	Pnma (No. 62)	9.1577	8.0332	10.2005	90	4	[13,28]

**Table 7:** Optical bandgap of As-S thin films by preparation method. All values from Tauc plots unless stated.

Material/Phase	E_g_ (eV)	Deposition Method	Film Thickness	Ref.
As_2_S_3_ bulk glass	~2.32 (293 K)	Melt-quenched	Bulk	[34]
As_2_S_3_ thermally evaporated	2.39	Thermal evaporation	~0.425 µm	[35]
As_2_S_3_ PLD (ML regime)	2.26 ± 0.02	Pulsed laser deposition	~4 µm	[40]
As_2_S_3_ PLD (QSML regime)	2.29 ± 0.02	Pulsed laser deposition	~4 µm	[40]
As_2_S_3_ nanocrystalline CBD	~2.20–2.58	Chemical bath deposition	~200–500 nm	[45]
As_4_S_4_ single crystal	2.40 ± 0.05	n.a.	Bulk	[30]
As_4_S_3_ thin film	~2.5	Vacuum sublimation	Not specified	[14]

**Table 8:** Electrical properties of the As-S phases, resistivity, carrier type and transport activation.

Phase	Form	Resistivity (Ω·cm)	Carrier Type	Mobility (cm^2^/Vs)	Transport Mechanism	Ref.
As_2_S_3_ amorphous	Thermally evaporated thin film	~10^9^–10^12^	p-type	n.a.	Hopping/band-tail activation	[83]
As_2_S_3_ amorphous	Thermally evaporated, In electrodes	~1.3 × 10^8^	n.a.	n.a.	Activated, E_a_ = 0.20 eV; localised states near E_F_	[65]
As_2_S_3_ amorphous	Solution spin-coated, annealed 150 °C	~7.7 × 10^7^	n.a.	n.a.	Activated, E_a_ = 0.12 eV; localised states	[65]
As_2_S_3_ nanocrystalline CBD	185 nm film, grain 11 nm	~5.1 × 10^5^	n-type	n.a.	Thermally activated, E_a_ = 0.15 eV	[45]
As_2_S_3_ nanocrystalline CBD	520 nm film, grain 38 nm	~3.5 × 10^4^	n-type	n.a.	Thermally activated, E_a_ = 0.07 eV	[45]
As_4_S_4_	Single crystal	>10^16^	Holes dominant	µ_h_ = 12; µ_e_ = 0.02	Holes: band/lattice scattering; electrons: hopping + trap-controlled	[30]
As_4_S_3_	Thin film, β-dominant (5.0 µm/h)	n.a.	n.a.	n.a.	SCLC, I ∝ U^3.2^, Δ = 0.055 eV	[15]

**Table 9:** Comparative summary of the optical, electrical and optoelectronic properties of the As-S thin-film phases.

Property	a-As_2_S_3_	c-As_4_S_4_	β-As_4_S_3_	α-As_4_S_3_	Source
Optical gap (eV)	2.3–2.4	2.40 ± 0.05	~2.5	~2.5	Section 5.1 [14,30,76]
Subgap band	not reported	not reported	none detected	~1.9 eV	Section 5.1 [14]
Refractive index at 633 nm	~2.5	not reported	not reported	not reported	Section 5.2
Dark resistivity (Ω·cm)	~10^9^–10^12^	>10^16^	not reported	not reported	Section 6.1
Carrier type	p-type	holes dominant	not reported	not reported	Section 6.1
Transport mechanism	hopping/band-tail	band holes; hopping electrons	SCLC I∝U^3.2^	too resistive to measure	Section 6.2 and Section 6.3 [15,30]
Trap energy width Δ (eV)	not reported	not applicable (discrete trap, 0.26 eV)	0.055 (air); 0.062 (propylamine)	not reported	Section 6.2 [15,30]
Photoconductivity maxima	not reported	rises to the 400 nm measurement limit; 2.07 eV subgap peak	4.28 eV	2.9 + 4.28 eV	Section 6.3 [14,30]
n_2_/FOM	100–150× silica; FOM~208	not reported	not reported	not reported	Section 5.5
Photodarkening	reversible, Δn~0.1–0.12	not applicable (photoconverts)	not reported	not reported	Section 4.7 and Section 5.6
PL emission	none detected	not reported	~2.05 eV (laser-induced)	not reported	Section 6.5 [6]
Gas sensing	not reported	not reported	propylamine 40–320 ppm	tested, too resistive	Section 6.4 [15]
UV photodetection	not reported	not reported	selective, 4.28 eV	dual-band, 2.9 and 4.28 eV	Section 6.3 [14]

## Data Availability

No new data were created or analyzed in this study. Data sharing is not applicable to this article.
